# A Chaos-Enhanced Binary Newton–Raphson Optimizer for High-Dimensional Sensor Data Feature Selection

**DOI:** 10.3390/s26123887

**Published:** 2026-06-18

**Authors:** Abdelmonem M. Ibrahim, Doaa A. Fakhry, Fares Al-Shargie

**Affiliations:** 1 Department of Mathematics, Faculty of Science, Al-Azhar University, Assiut Branch, Assiut 71524, Egypt; abdelmonem@azhar.edu.eg; 2 Computer Science Branch, Department of Mathematics, Faculty of Science, Assiut University, Assiut 71516, Egypt; doaaahmedfakhry@gmail.com; 3 Department of Information Technology, Faculty of Industry and Energy Technology, New Assiut Technological University (N.A.T.U.), Assiut 71684, Egypt; 4 Department of Rehabilitation and Movement Sciences, Rutgers University, Newark, NJ 07102, USA

**Keywords:** metaheuristics, chaotic maps, feature selection, wrapper approach, classification, optimization algorithms, neuroimaging

## Abstract

Feature selection is crucial for high-dimensional sensor and biomedical data because it reduces redundancy, improves generalization, and supports interpretable biomarker discovery. In this study, we propose a Binary Chaos-Enhanced Newton–Raphson-Based Optimizer (BCNRBO) for wrapper-based feature selection. The method integrates chaotic search dynamics, a Hamming-distance-based Dynamic Potential mechanism, and a new binary transfer function to enhance exploration and prevent premature convergence. BCNRBO was evaluated on 26 benchmark datasets using a variety of classifiers, including K-nearest neighbor (KNN), decision tree (DT), Naive Bayes (NB), and Support Vector Machine (SVM) classifiers. The proposed method consistently achieved competitive or superior classification performance while selecting fewer features than competing binary metaheuristic methods. In particular, BCNRBO consistently achieved the best feature reduction performance across all classifiers and secured the top Friedman rankings for DT, NB, and SVM, demonstrating its overall effectiveness. Statistical tests confirmed significant improvements over competing methods in most pairwise comparisons. These results suggest that BCNRBO is a promising feature selection strategy for sensor-derived biomedical and neurorehabilitation data, where compact and reliable digital biomarkers are needed.

## 1. Introduction

One of the pre-processing steps in machine learning is feature selection (FS). For complex or huge datasets, selecting the most relevant feature subset is one of the most difficult challenges [1,2]. The search for hidden patterns or important information in massive amounts of data has become a critical issue. It has been demonstrated that feature selection efficiently eliminates superfluous and irrelevant features. FS is a pre-processing method that selects relevant features to reduce overfitting, enhance model accuracy and interpretability, speed up learning, and reduce dataset memory needs [3]. It can also decrease the amount of storage needed, lower the computational cost, and enhance classifier performance. Both the number of attributes/features and the number of instances of data have increased in recent years [4]. Computational procedures known as feature selection algorithms are used to choose a collection of characteristics that maximizes an assessment metric that represents the quality of the characteristics [5]. There are two important factors to consider when choosing the optimum feature subsets, according to the literature: maximizing classification accuracy and minimizing the attributes found in the datasets [6,7].

An important consideration in dimension reduction is the evaluation metrics, which establish the quality of the chosen features. Dimension reduction techniques are typically separated into two major classes based on the evaluation measures: wrapper approaches and filter approaches, where a learning/classification method is used in wrapper approaches to determine the selected properties [8].

As a result, wrappers typically outperform filter approaches in classification. However, they suffer from high processing costs and a loss of generality, which makes them specific to a single classification algorithm [9]. Any learning algorithm has no bearing on filter methods. Therefore, appropriate evaluation metrics are typically required for filter approaches. Wrappers typically outperform filter approaches in terms of classification performance but may incur significant processing costs and a loss of generality due to algorithm specificity [10].

The size of the search space in dimension reduction issues increases exponentially with the number of attributes. In most situations, a thorough search is not necessary. Heuristic search techniques for dimension reduction have limitations, such as lengthy computation times and being stuck in local optima. To handle dimension reduction challenges, it is recommended to use the cheapest global search technique [11,12].

FS is typically performed as a pre-processing stage before model construction, utilizing specialized algorithms [13,14]. Data mining and machine learning researchers are actively researching the creation of feature selection algorithms (FSAs). FSAs are computational methods that pick a set of features to optimize an assessment measure of feature quality [15,16].

In recent years, various new metaheuristic algorithms have been introduced to overcome the disadvantages of exact algorithms and to improve the performance of other metaheuristics [17]. Exact algorithms are not able to solve complex high-dimensional problems in a reasonable time because the search space increases exponentially with the problem size. Some of the metaheuristic algorithms have provided outstanding performance in solving the problems. Most of them have been implemented for real search spaces [18].

Hence, different techniques have been proposed to use metaheuristic algorithms in the binary search, such as the Whale Optimization Algorithm (WOA) [19], Ant Colony Optimization (ACO) [20], the Bat Algorithm (BA) [21], the Artificial Bee Colony (ABC) [22], Particle Swarm Optimization (PSO) [23], Biogeography-Based Optimization (BBO) [24], the Genetic Algorithm (GA) [25], the Harmony Search Algorithm (HSA) [26], the Flower Pollination (FP) algorithm [27], the Grasshopper Optimization Algorithm (GOA) [28], and the Binary Dragonfly (BDF) algorithm [29].

Chaotic maps have been increasingly integrated into metaheuristic algorithms to improve exploration, maintain diversity, and avoid premature convergence. By replacing random components with deterministic chaotic sequences, these algorithms can better navigate complex search spaces and escape local optima. Recent studies have shown the effectiveness of chaos-enhanced metaheuristics: a chaotic transient search optimization algorithm improves global search performance [30], the Binary Chaotic Crow Search Algorithm (BCCSA) [31], while a chaotic dwarf mongoose optimizer enhances feature selection accuracy [32].

For continuous optimization problems, the Newton–Raphson-Based Optimizer is among the most recently used metaheuristic algorithms [33]. It is straightforward and simple to put into practice. It also has a few control parameters that need to be modified. It has been widely applied to resolve a variety of optimization issues in the real world. Recent studies have focused on enhancing the NRBO algorithm across a range of application domains. For example, a multi-strategy improved version of NRBO has been proposed for engineering optimization problems [34], while a population-based variant has been developed for continuous optimization tasks [35]. In another application, an enhanced NRBO-XGBoost model was introduced for the remote sensing of shallow water depth in the nearshore regions of the Beibu Gulf [36]. NRBO has also been applied to multi-level threshold image segmentation for tomato plant disease detection [37]. Given that neuroimaging and wearable rehabilitation data are typically high-dimensional, noisy, and redundant, feature selection becomes a crucial step for extracting stable digital biomarkers, reducing computational complexity, and improving clinical interpretability.

The main problem addressed in this study is the limitation of existing binary optimization algorithms in handling high-dimensional feature selection tasks, particularly in avoiding premature convergence. Therefore, this work aims to propose an enhanced binary optimizer (BCNRBO) that improves search diversity and solution quality.

In this work, a novel binary chaotic variant of the Newton–Raphson-Based Optimizer, referred to as BCNRBO, is introduced. Different chaotic maps are examined to identify the most effective one for integration into the proposed algorithm, with the goal of improving search efficiency and enhancing feature selection performance. The selected chaotic map is utilized through a Chaotic Enforcement operator to dynamically adjust and improve the step size during the optimization process. Moreover, BCNRBO incorporates a new mechanism called Dynamic Potential, which is formulated as a time-varying potential function that adapts throughout the search process to regulate the algorithm’s behavior. The DP mechanism is based on the Lennard-Jones potential [38], in which the Hamming distance [39] is embedded to quantify the interaction force between candidate solutions and promote population diversity. In addition, a novel transfer function is proposed to transform continuous solutions into binary representations. This transfer function is driven by chaotic values and follows an S-shaped form, enabling an effective balance between exploration and exploitation in the binary search space.

In order to maximize classification accuracy, choose the most advantageous features that minimize the dataset size and enhance algorithm stability; the performance of the proposed BCNRBO method was evaluated using 26 different benchmark datasets, including medical datasets with different sizes (low, medium, and large scale). The results show that the proposed BCNRBO achieves better results than other similar algorithms in the literature, such as: the Binary Arithmetic Optimization Algorithm (BAOA) [40], the Binary Bat Algorithm (BBA) [41], the Binary Flower Pollination Algorithm (BFPA) [42], Binary Particle Swarm Optimization (BPSO) [43], the Binary Chaotic Crow Search Algorithm (BCCSA) [31], Binary Atom Search Optimization (jBASO) [44], and Binary Dwarf Mongoose (BDA) [32].

The rest of this paper is organized as follows: Section 2 introduces the preliminaries, where Section 2.1 provides the background information, and Section 2.2 gives an outline of the standard Newton–Raphson-Based Optimizer, chaotic maps, and the potential technique. Section 3 presents the proposed binary chaotic Newton–Raphson-Based Optimizer. Section 4 describes the computational experiment design for the feature selection problem. Section 5 presents the experimental results and discussions. Section 6 concludes this study and provides insight into future trends.

## 2. Preliminaries

### 2.1. Background

Recent advances in metaheuristic optimization algorithms have demonstrated their effectiveness in solving feature selection and classification problems, particularly in high-dimensional search spaces. For example, human-inspired and nature-inspired optimization techniques have attracted increasing attention due to their strong exploration and exploitation capabilities [45]. The cultural history optimization algorithm has been proposed as an effective human-inspired metaheuristic for engineering optimization tasks [8]. In addition, several hybrid frameworks integrating swarm intelligence with machine learning models, such as PSO-LSTM and the Whale Optimization Algorithm combined with LSTM, have shown improved convergence behavior and classification accuracy.

Furthermore, comprehensive studies on Elephant Optimization Algorithms [46] and Mountain Gazelle Optimizers [45] have provided valuable insights into algorithm classification, application domains, and future research directions. Recently, adaptive optimization techniques combined with deep learning have also been successfully applied to classification and cyber security problems, such as phishing email detection. These studies highlight the importance of population diversity enhancement, adaptive search mechanisms, and hybridization strategies, which motivate the development of the proposed BCNRBO for feature selection.

Recent advances in learning-based feature selection and optimization techniques have shown promising results across various domains. Zhou et al. [47] proposed an attention-based interaction-aware spatio-temporal graph neural network (AST-GNN) for trajectory prediction, demonstrating the effectiveness of modeling complex dependencies through deep neural architectures. Fan et al. [48] introduced an adaptive data structure regularized multiclass discriminative feature selection method, which effectively captures the underlying structure of high-dimensional datasets for improved classification performance. Furthermore, Wu et al. [49] presented ReffaceNet, a reference-based framework for face image generation from line art drawings, highlighting recent developments in neural network-based representation learning.

In contrast, metaheuristic optimization methods, such as the proposed BCNRBO, provide flexible and computationally efficient alternatives capable of handling high-dimensional binary search spaces while maintaining competitive classification accuracy. This motivates the integration of chaotic dynamics into NRBO to enhance the exploration–exploitation balance in binary feature selection tasks.

On the other hand, chaotic maps have been widely adopted in metaheuristic optimization to improve exploration capabilities, enhance diversity, and mitigate premature convergence by replacing or augmenting traditional random components with deterministic yet unpredictable sequences. Instead of relying solely on pseudo-random number generators, sequences produced by chaotic maps allow algorithms to explore the search space more thoroughly and avoid stagnation in local optima, leading to better overall search performance in many applications. Recent studies have systematically analyzed the role of chaos in metaheuristics, showing significant improvements for variants of many well-known algorithms, such as chaotic Particle Swarm Optimization, chaotic whale and sine cosine algorithms, and other chaos-enhanced population-based methods across benchmark and engineering problems [30,50,51]. Moreover, chaos-based feature selection approaches have demonstrated enhanced convergence and accuracy compared with their non-chaotic counterparts by integrating chaotic maps directly into initialization, parameter control, and transfer functions [31,52]. These results highlight the increasing effectiveness of chaos integration as a strategy for improving modern metaheuristic algorithms.

To provide a clear overview of the existing research and highlight the key differences among prior studies, a summary table of the reviewed papers is presented below (Table 1). This table compares the algorithms, datasets, and main contributions, which helps to identify the remaining research gaps and justify the proposed approach.

### 2.2. Newton–Raphson-Based Optimizer

**Figure 1:**
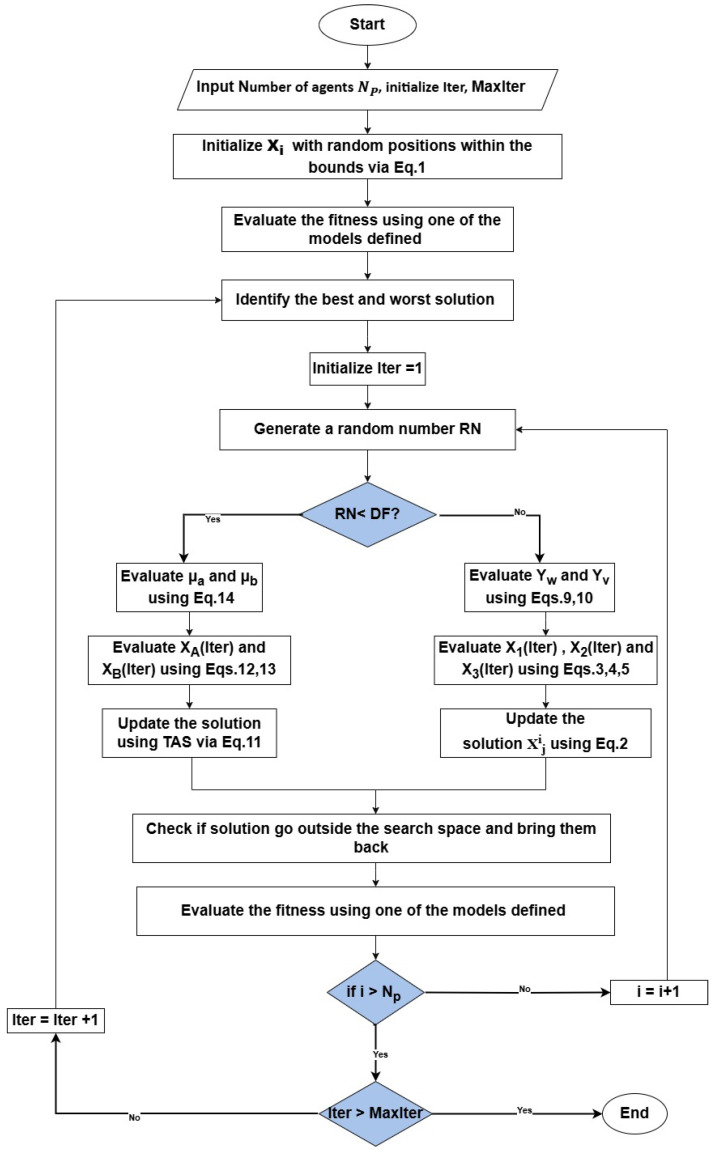
NRBO flowchart.

The Newton–Raphson-Based Optimizer is an advanced optimization algorithm inspired by the Newton–Raphson method, which is commonly used to find solutions to non-linear equations. NRBO leverages iterative approximation to efficiently converge to the optimal solution by adjusting the search parameters based on the gradient and curvature of the objective function [62]. Newton’s approach uses gradient information to iteratively improve initial estimates and guide the search for solutions [33]. NRBO introduces three key principles for exploring solution spaces: using gradients to reach optimal points, iterative adjustments with random variations to maintain diversity and avoid premature convergence, and using a trap avoidance strategy to navigate around local optimum points. Further details are provided in the flowchart shown in Figure 1.

#### 2.2.1. Initialization and Fitness Evaluation

NRBO is a population optimization system that produces and updates several agents $N_{P}$ during each iteration (Iter) until the maximum number of iterations (MaxIter) is reached. Each agent is addressed by a decision vector $X_{n}^{j}$ of dimensionality Dim. The solution vectors for agents are randomly assigned within the specified boundaries ($Upper^{j}\;\mathrm{and}\;Lower^{j}$) and are calculated as follows:

(1) $$X_{i}^{j}\left(Iter=1\right)=Lower^{j}+\left([Upper^{j}-Lower^{j}]\times rand\right),\;i=1:N_{P},\;j=1:Dim,$$

where *rand* denotes the random number. For each agent $X_{i}^{j}$ in the population, the objective function is evaluated. The agents are ranked based on their fitness values.

The parameter Draft Factor (DF) is responsible for determining the strategy compared between the following two phases. The value of DF is set to be equal to 0.6 as shown in the original paper.

#### 2.2.2. Newton–Raphson Search Rule (NRSR)

The NRSR determines the agents, allowing for improved exploration of the feasible zone and better positioning. The NRSR is used to update each agent’s position in the population using gradient-based information for optimization. This rule uses a formula to update agents’ locations based on relative fitness values iteratively. The update method incorporates a random perturbation factor (ΔX) that is proportionate to the difference between the best-performing and current agents. This increases the diversity of solutions. The NRSR formula calculates a weighted difference (ΔX) between two solution vectors. This method changes agents’ positions instead of their fitness values, resulting in more efficient computation and fewer recalculations in the following steps.

Solutions are generated by combining three components, each perturbed randomly to encourage diverse exploration. The primary components ($X_{1},X_{2},$ and $X_{3}$) for each solution are calculated iteratively using factors that account for previous best solutions and random elements. At each iteration, the location of the agent ($X_{i}^{j}$) is updated based on weighted averages of ($X_{1},X_{2},$ and $X_{3}$), as shown below:

(2) $$\begin{array}{cc} X_{i}^{j}\left(Iter+1\right)=rd_{1}^{j}\times \left(rd_{1}^{j}\times X_{1i}^{j}\left(Iter\right)+\left(1-rd_{1}^{j}\right)\times X_{2i}^{j}\left(Iter\right)\right) & +\left(\left(1-rd_{1}^{j}\right)\times X_{3i}^{j}\left(Iter\right)\right),\\ & \;i=1:N_{p},\;j=1:Dim, \end{array}$$

where $X_{1i}^{j}\left(Iter\right)$, $X_{2i}^{j}\left(Iter\right)$, and $X_{3i}^{j}\left(Iter\right)$ are formulated with additional random variations and factors that incorporate differences between the best and worst agents, as well as the mean of current positions as follows:

(3) $$X_{1i}^{j}\left(Iter\right)=X_{i}\left(Iter\right)-\left(randn\times \left(\frac{(Y_{W}-Y_{V})\times \Delta X}{2\times (Y_{W}+Y_{V}-2\times X_{i})}\right)\right))+\rho ,$$

(4) $$X_{2i}^{j}\left(Iter\right)=X_{best}^{j}-randn\times \left(\frac{(Y_{W}-Y_{V})\times \Delta X}{2\times (Y_{W}+Y_{V}-2\times X_{i})}\right)+\rho ,$$

(5) $$X_{3i}^{j}\left(Iter\right)=X_{i}\left(Iter\right)+\delta \times \left(X_{2i}^{j}\left(Iter\right)-X_{1i}^{j}\left(\mathrm{Iter}\right)\right),$$

where randn denotes the normal-distributed random number with a mean of zero and a variance of one, and $rd_{1}^{j}$ is a random value between 0 and 1. The parameter δ is calculated from the following equation:

(6) $$\delta ={\left(1-\frac{2\cdot Iter}{MaxIter}\right)}^{5}.$$

The exploitation of NRBO is improved by including another parameter called ρ, which directs the population in the right direction. The expression for ρ is given as follows:

(7) $$\rho =a\times \left(X_{best}-X_{i}\left(Iter\right)\right)+b\times \left(X_{r1}\left(Iter\right)-X_{r2}\left(Iter\right)\right),$$

where $X_{r1}\left(Iter\right)$ and $X_{r2}\left(Iter\right)$ correspond to two different randomly chosen vector agents among the population, and *a* and *b* are arbitrary values that range from 0 to 1. The components of $Y_{V}$ and $Y_{W}$ can be derived using the following equations:

(8) $$Y_{V}=rd_{2}^{j}\times \left(Mean(X_{i}-randn\times \left(\frac{(X_{worst}-X_{best})\times \Delta X}{2\times (X_{worst}+X_{best}-2\times X_{i})}\right),X_{i})\right)-rd_{2}^{j}\times \Delta X,$$

(9) $$Y_{W}=rd_{2}^{j}\times \left(Mean(X_{i}-randn\times \left(\frac{(X_{worst}-X_{best})\times \Delta X}{2\times (X_{worst}+X_{best}-2\times X_{i})}\right),X_{i})\right)+rd_{2}^{j}\times \Delta X,$$

where $X_{best}$ denotes the best position found so far, $X_{worst}$ represents the worst position, $rd_{2}^{j}$ is a random value between 0 and 1, and ΔX indicates its weighted differences described as follows:

(10) $$\Delta X=rand\;\times \;|X_{best}-X_{i}|,\;rand\in \left[0,1\right].$$

#### 2.2.3. Trap Avoidance Strategy (TAS)

In NRBO, the TAS enhances the suggested NRBO capability to address real-world problems and is used to help agents escape local optima and avoid premature convergence.

This technique creates new positions by using the best-performing agent ($X_{best}$) and the current agent’s position ($X_{i}\left(Iter\right)$).

(11) $$X_{TAS}\left(Iter\right)=\left\{\begin{array}{cc} X_{i}\left(Iter+1\right)+X_{A}\left(Iter\right)+X_{B}\left(Iter\right), & \mathrm{if}\;\;\mu_{a}<0.5\\ X_{best}+X_{A}\left(Iter\right)+X_{B}\left(Iter\right), & otherwise, \end{array}\right.$$

(12) $$X_{A}\left(Iter\right)=\theta_{1}\times \left(\left(\mu_{a}\times X_{best}\right)-\left(\mu_{b}\times X_{i}\left(Iter\right)\right)\right),$$

(13) $$X_{B}\left(Iter\right)=\left(\theta_{2}\times \delta \right)\times \left(\left(\mu_{a}\times Mean\left(X_{i}\right)\right)-\left(\mu_{b}\times X_{i}\left(Iter\right)\right)\right),$$

where $\theta_{1}$ and $\theta_{2}$ are uniform random numbers between (−1,1) and (−0.5,0.5), respectively, *DF* denotes the deciding factor that controls NRBO performance, and $\mu_{a}$ and $\mu_{b}$ are random numbers, which are generated using Equation (14) and Equation (15), respectively.

(14) $$\mu_{a}=\left(1-\beta \right)+3\times \beta \times rand,\;\mu_{b}=\left(1-\beta \right)+\beta \times rand,$$

(15) $$\beta =\left\{\begin{array}{cc} 0, & \mathrm{if}\;rand>0.5\\ 1, & \mathrm{otherwise}, \end{array}\right.$$

where *rand* denotes the uniform random number between (0,1) and β denotes a binary number, either one or zero. If the value of rand is greater than or equal to 0.5, then the value of β is zero; otherwise, the value is one. Because of the randomness in the selection of the parameters $\mu_{a}$ and $\mu_{b}$, the population becomes more diverse and escapes from the local optimum solutions, which helps to improve its diversification.

For each agent $X_{i}$ in the population, the objective function is evaluated. The agents are ranked according to their fitness values to identify the best agent $X_{best}$ with the lowest fitness value and the worst agent $X_{worst}$ with the highest fitness value.

The following steps summarize the NRBO procedure:

Step 1: Specify the NRBO parameters: $Dim,\;Lower,\;Upper,\;N_{p},\;MaxIter,\;a,\;b$.
Step 2: Initialize the solution vectors using Equation (1).
Step 3: For each solution vector, evaluate the fitness function to assess the performance of each solution.
Step 4: Rank the solutions based on their fitness values. Identify the agent with the lowest fitness as the best and the one with the highest fitness as the worst.
Step 5: Generate a random number RN to determine the strategy compared with the Draft Factor (DF = 0.6) to:
  - Apply the NRSR using Equation (2) to update agent positions using gradient-based information for local refinement.
  - Apply the TAS using Equation (11) to enhance exploration and help agents escape local optima.
Step 6: Re-evaluate the fitness function for the updated design variables.
Step 7: Repeat steps 3–6 until the maximum number of iterations is reached. Once complete, the final position of the best agent is considered the optimal solution.

### 2.3. Chaotic Maps

Chaotic maps are deterministic systems that respond to initial conditions and exhibit disorganized or random behavior. Chaos theory investigates both deterministic and dynamic system behaviors. Depending on the initial conditions, chaotic variables can cycle through all states in specific periods without repetition. Creating chaotic number sequences is quick and easy. Chaos search outperforms random search when it comes to escaping the local optimum due to its unique tendencies [50,63].

Chaos refers to dynamical processes that appear random but are actually generated by regular systems. This behavior is typical of stochastic processes, such as the movement of molecules in a gas-filled vessel. However, chaotic systems are inherently non-linear; typical statistical approaches that are frequently linear are insufficient for their analysis. Chaotic system characteristics are similar to random-like systems, making them readily confused with random noise.

In this study, the chaotic maps will be incorporated into the binary NRBO in order to enhance its performance.

**Figure 2:**
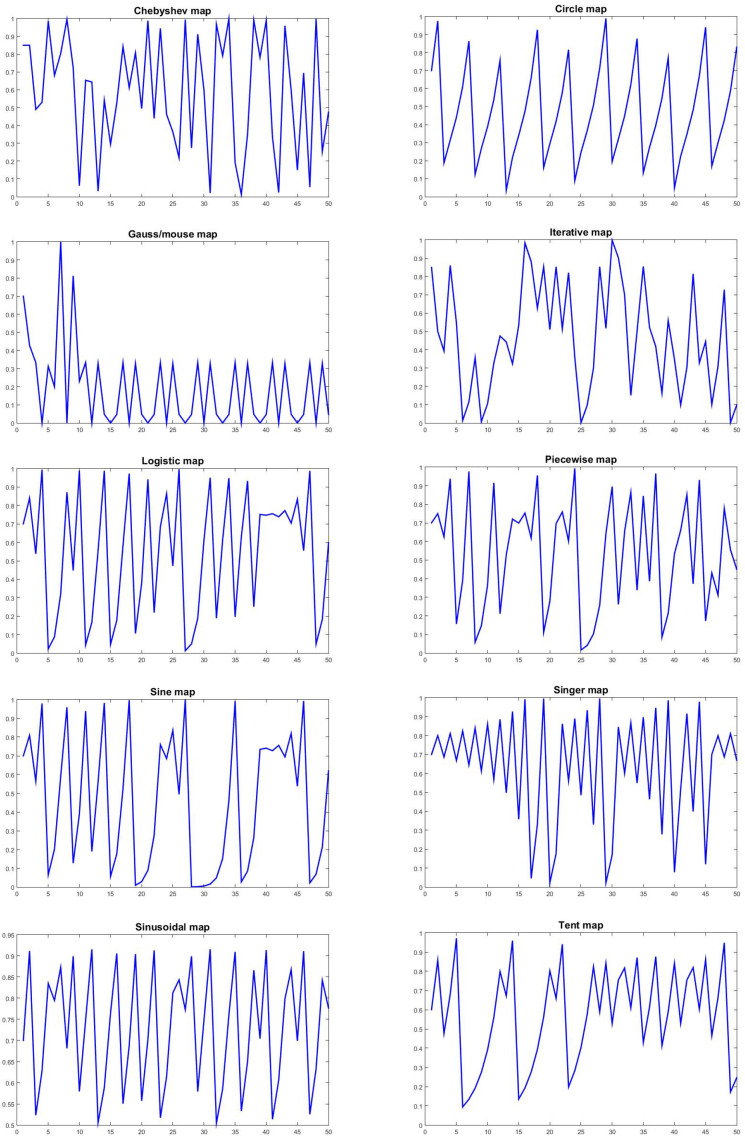
Visualization of chaotic maps.

Ten chaotic maps are used in this study, namely the Chebyshev, Circle, Gauss/Mouse, iterative, Logistic, piecewise, sine, Singer, Sinusoidal and Tent maps. Table A1 contains additional specific information about the common mathematical equations of the shared chaotic maps. These chaotic maps are visualized in Figure 2 for 10 different chaotic map values. Most chaotic maps used in integrating chaos theory into metaheuristic algorithms are either one- or two-dimensional. In the proposed algorithm, one-dimensional chaotic maps are used as random number generators at the beginning of the search process to improve the performance of BCNRBO, find effective solutions in the search space, and increase the stability of the algorithm for solving FS problems. The starting value was assigned randomly within the range of [0, 1] for all chaotic maps used in the proposed algorithm.

As shown in Figure 2, Map10 exhibits significantly different chaotic behavior compared to the other maps. While several maps demonstrate periodic tendencies or limited oscillation ranges, Map10 maintains a highly irregular and non-periodic pattern throughout the iterations. Moreover, the amplitude of Map10 varies dynamically, indicating stronger chaotic properties and better randomness, which is particularly beneficial for binary optimization and feature selection problems [64].

### 2.4. Lennard Potential

The Lennard potential is a mathematical model that describes how atoms or molecules interact with each other based on their distance [38,65]. In this technique, the solution maintains a relative distance that varies within a certain range at all times due to repulsion or attraction. The change in amplitude of the repulsion relative to the equilibrium distance is significantly greater than that of the attraction.

The Lennard-Jones potential is used as the interaction force acting on the current atom from the other atom in the *j*th dimension at Iter time, which can be written as:

(16) $$P=n\left(Iter\right)\times \left(12{\left(-h_{i}^{j}\right)}^{-13}-6{\left(-h_{i}^{j}\right)}^{-7}\right).$$

The function behaviors of *P* with different values of n(Iter) correspond to the values of $h_{i}^{j}$ ranging from 0.9 to 2. The n(Iter) is the depth function of the current iteration to adjust the repulsion region or the attraction region based on the maximum number of iterations (MaxIter), which can be defined as:

(17) $$n\left(Iter\right)={\left(1-\frac{Iter-1}{MaxIter}\right)}^{3}.$$

The behavior of the function n(Iter) varies with different values of *h*, as defined in Equation (16), within the range of 0.9 to 2. For varying n(Iter) values, repulsion occurs when $h_{i}^{j}\in \left[0.9,1.12\right)$, while attraction takes place when $h_{i}^{j}\in \left(1.12,2\right]$. Equilibration is observed precisely at $h_{i}^{j}=1.12$. Notably, the attraction effect diminishes to nearly zero when $h_{i}^{j}\geq 2$. To enhance exploration capabilities, $h_{i}^{j}$ is defined as:

(18) $$h_{i}^{j}\left(Iter\right)=\left\{\begin{array}{cc} h_{\min} & \frac{r_{i}^{j}\left(Iter\right)}{\sigma (Iter)}<h_{\min}\\ \frac{r_{i}^{j}\left(Iter\right)}{\sigma (Iter)} & h_{\min}\leq \frac{r_{i}^{j}\left(Iter\right)}{\sigma (Iter)}\leq h_{\max}\\ h_{\max} & \frac{r_{i}^{j}\left(Iter\right)}{\sigma (Iter)}>h_{\max}, \end{array}\right.$$

where $h_{\min}$ and $h_{\max}$ are the lower and upper limits of $h_{i}^{j}$, respectively. The main equations of the function help maintain diversity among candidate solutions while refining their positions over iterations.

The amplitude of the repulsion change with respect to the equilibration distance is significantly larger than the attraction change, which is equal to r=1.12σ, where the length scale σ(Iter) is defined as

(19) $$\sigma \left(Iter\right)={∥x_{y}\left(Iter\right),\frac{1}{\left|K(Iter)\right|}\sum_{j\in K_{\mathrm{best}}}x_{y}\left(Iter\right)∥}_{2},$$

where $K_{\mathrm{best}}$ is a subset of an agent population, consisting of the first *K* neighbor agents with the best fitness values. The system boundaries are defined as [65]:

(20) $$\left\{\begin{array}{c} h_{\min}=g_{0}+g\left(Iter\right)\\ h_{\max}=u, \end{array}\right.$$

where $g_{0}$ and *u* are values of *h* corresponding to values between the minimum value and maximum value of the attraction. The drift factor g(Iter), which transitions the algorithm from exploration to exploitation, is given by

(21) $$g\left(Iter\right)=0.1\times \sin\left(\frac{\pi }{2}\times \frac{Iter}{MaxIter}\right).$$

## 3. BCNRBO: Binary Chaotic Newton–Raphson-Based Optimizer

Metaheuristics were often created to address ongoing optimization problems. But a lot of optimization issues (like the FS problem) have a binary search space. It is necessary to modify an algorithm in order to solve binary problems. So, in this study, we introduce the binary version of the improved NRBO.

In order to help agents avoid early convergence and escape local optima, the following steps improve the proposed BCNRBO capability to handle real-world problems such as feature selection. The main contributions of this study are summarized as follows:

First, the original trap avoidance strategy (TAS) utilized in the continuous NRBO was deliberately omitted in the proposed BCNRBO framework. While the TAS is essential in the continuous version to mitigate stagnation in local optima, its integration into the binary design introduces redundant computational overhead and behavioral conflicts. In BCNRBO, the exploration–exploitation balance and population diversity are already robustly maintained by the synergy between chaotic maps and the Hamming-distance-based Dynamic Potential (DP) mechanism. The DP mechanism adaptively applies repulsive forces when binary solutions cluster too closely, inherently preventing premature convergence. Preliminary empirical testing confirmed that omitting the TAS not only preserves execution time but also maintains or slightly enhances classification accuracy by eliminating unnecessary algorithmic redundancy.

Second, we improve the new population in the NRSR phase by changing the random numbers (*a* and *b*) into a new parameter called “Dynamic Potential”.

Third, since chaotic maps have been shown to increase the effectiveness of optimization algorithms, we incorporate them in certain places throughout the proposed method.

Lastly, we utilize a modification of NRBO’s parameter δ to propose a new transfer function that depends on both the s-shape and v-shape functions, thereby transforming the continuous solutions into binary ones.

In the beginning, the initial positions are initialized in binary form as follows:

(22) $$X_{i}^{j}\left(Iter=1\right)=\left\{\begin{array}{cc} 0, & \;\mathrm{if}\;rand\leq 0.5,\\ 1, & \;\mathrm{if}\;rand>0.5, \end{array}\right.$$

where $X_{i}^{j}$ is the *j*th dimension of the *i*th agent, Iter is the current iteration, and rand is a random number from the distributions uniformly distributed over the interval [0,1]. The objective function is assessed, and the fitness values of the agents are stored for every initial binary agent in the population.

The next subsections will provide a detailed explanation of all the proposed main steps, which are utilized to enhance the quality of the updated solutions.

### 3.1. Dynamic Potential (DP)

Dynamic Potential is a time-dependent potential function that evolves during the search process to adaptively influence algorithm behavior, rather than remaining fixed. Dynamic Potential adapts according to the iteration or time step, allowing the algorithm to guide the search process more effectively while preserving optimal behavior [66].

In this study, to enhance the exploration of the proposed algorithm in the first stage of iterations, each agent needs to interact with as many agents as possible with a better fitness value than its $K_{best}$ neighbors. The $K_{best}$ is calculated from the following equation:

(23) $$K_{best}=\left\lceil N_{p}-\left(N_{p}-2\right)\times \sqrt{\frac{Iter}{MaxIter}}\right\rceil ,$$

where “⌈·⌉” is the ceil value, $N_{p}$ is the total number of agents (population size), Iter is the current iteration, and MaxIter is the maximum number of iterations. It is worth mentioning that $K_{best}$ is an integer number that starts close to $N_{p}$ and then gradually decreases to approximately the value of two during the search progress (Equation (23)).

To update the new guiding position, the following steps describe how to compute the probability $P_{i}$ for each agent based on the new parameter called mass ($M_{i}$) of the *i*th agent, which is described as a probability distribution or fitness-based ranking of agents. It can be calculated as follows:

(24) $$P_{i}=\frac{M}{\sum M_{i}},\;M_{i}=\exp\left(\frac{{\mathrm{Fitness}}_{best}-{\mathrm{Fitness}}_{1,\ldots ,N_{p}}}{{\mathrm{Fitness}}_{worst}-{\mathrm{Fitness}}_{best}}\right),$$

where the best and worst fitness values for a minimization problem at iteration Iter are calculated for the current population as follows:

(25) $${\mathrm{Fitness}}_{best}\left(Iter\right)=\min_{i\in [1,\ldots ,N_{p}]}{\mathrm{Fitness}}_{i}\left(Iter\right),\;{\mathrm{Fitness}}_{worst}\left(Iter\right)=\max_{i\in [1,\ldots ,N_{p}]}{\mathrm{Fitness}}_{i}\left(Iter\right),$$

where ${\mathrm{Fitness}}_{i}\left(Iter\right)$ is the value of the objective function for the *i*th individual in iteration Iter, and $N_{p}$ is the size of the population.

A new guiding position XK(Iter) is determined as the mean values of the selected $K_{best}$ agents based on the mass $M_{i}$ as follows:

(26) $$XK_{i}\left(Iter\right)=\frac{\sum_{m=1}^{K_{best}}X_{m}}{K_{best}},$$

where $m=1,2,\ldots ,K_{\mathrm{best}}$ is the index of the sorting $P_{i}$ (Equation (24)) for each agent. It is sorted descendingly from highest to lowest values ($M_{i}^{\left(1\right)}\geq M_{i}^{\left(2\right)}\geq \cdots \geq M_{i}^{\left(N_{P}\right)}$).

In the next step, for binary or discrete optimization problems, we employ the Hamming distance (hd) [39,67], which measures the dissimilarity between two position vectors $X_{1}$ and $X_{2}$ by counting differing elements as follows:

(27) $$hd\left(X_{1},X_{2}\right)=\sum_{j=1}^{Dim}\left(X_{1}^{j}\neq X_{2}^{j}\right).$$

The Hamming-distance calculation served as a technique to accommodate the discrete nature of the search space. By incorporating the Hamming distance, the algorithm can effectively measure and navigate the differences between potential solutions, thereby improving its ability to find optimal or near-optimal solutions in optimization problems.

From a physical interpretation perspective, the Lennard-Jones potential function conventionally models inter-atomic forces based on continuous spatial coordinates. To bridge this physical phenomenon with the discrete feature selection domain, the proposed framework maps these interactions onto a binary hypercube via the Hamming distance (hd). The ratio $\frac{hd(X_{i},X_{best})}{hd(X_{i},XK_{i})}$ (Equation (30)) acts as a dynamic structural equilibrium indicator, mimicking the ratio between actual atomic distance and characteristic length scale. When binary solutions cluster too tightly (low Hamming distance), they share near-identical feature configurations, leading to a drop in search diversity. In response, the Lennard-Jones formulation triggers a dominant mathematical repulsion force, which physically represents a structural push to enforce diversity. Conversely, when the solutions diverge excessively, a mild attractive potential dominates, gracefully pulling the agents back toward the promising search fields defined by the elite components.

Specifically, the type of interaction force—whether it is attractive or repulsive—between an agent and its neighbors depends on the ratio between their distance and the characteristic length scale $r_{i}$. This length scale, $\sigma_{i}$, indicates how far each agent is from the average position of its top $K_{best}$ nearest neighbors.

In this step, for each agent, the values of $r_{i}$ and $\sigma_{i}$ will be replaced by new values that are calculated based on the Hamming distance (Equation (27)) as follows:

(28) $$\sigma_{i}\left(Iter\right)=hd\left(X_{i},XK_{i}\right)=\sum_{i=1}^{n}\left(X_{i}\left(Iter\right)\neq XK_{i}\left(Iter\right)\right),$$

(29) $$r_{i}\left(Iter\right)=hd\left(X_{i},X_{best}\right)=\sum_{i=1}^{n}\left(X_{i}\left(Iter\right)\neq X_{best}\left(Iter\right)\right).$$

The new values of $h_{i}$ are computed after updating the parameters $\sigma_{i}$ and $r_{i}$, as follows:

(30) $$h_{i}=\left\{\begin{array}{cc} h_{min}, & \mathrm{if}\;\frac{hd(X_{i},X_{best})}{hd(X_{i},XK_{i})}<h_{min}\\ h_{max}, & \mathrm{if}\;\frac{hd(X_{i},X_{best})}{hd(X_{i},XK_{i})}>h_{max}\\ \frac{hd(X_{i},X_{best})}{hd(X_{i},XK_{i})}, & \mathrm{otherwise}, \end{array}\right.$$

where $h_{min}$ and $h_{max}$ are calculated from Equation (20), where $g_{0}$ in this study is equal to (h=1.12) when the attraction reaches the minimum value, and *u* is equal to (h=1.24) when the attraction reaches the maximum value. The value of *g* is calculated by Equation (21).

The ultimate value of the potential represents the interaction force between two distinct agents and is updated based on the new modified $h_{i}$ as follows:

(31) $$\mathrm{DP}=n\left(Iter\right)\left(12{\left(-h_{i}\right)}^{-13}-6{\left(-h_{i}\right)}^{-7}\right),$$

where n(Iter) is the depth function to regulate the attraction or repulsion region, and it can be calculated from Equation (17). See Algorithm 1 for further information and the steps involved in the Dynamic Potential.
**Algorithm 1** Pseudocode for Dynamic Potential**Require:** Iter (iteration), MaxIter (maximum iterations), $H_{0}=1.12$ and u=1.24 **Ensure:** Updated Dynamic Potential   1:Generate $K_{best}$ from Equation (23)  2:Calculate mass of agent (*M*) from Equation (24)  3:Extract new guiding position XK(Iter) based on $K_{best}$ from Equation (26)  4:Calculate the Hamming distances $\sigma =hd_{1}$ between the current solution and the current XK based on Equation (28)  5:Calculate the Hamming distances $r_{i}=hd_{2}$ between the current solution and the best one based on Equation (29)  6:Calculate n(Iter) from Equation (17) and g(Iter) from Equation (21)  7:Calculate $h_{\min}$ and $h_{\max}$ using Equation (20)  8:Calculate $h_{i}$ from Equation (30)  9:Calculate Dynamic Potential DP using Equation (31)10:**return** $DP_{i}$ value


### 3.2. Chaotic Enforcement (CE)

To avoid premature convergence to local optima and effectively guide solutions toward promising regions of the search space, we introduce a new chaotic map-based component named Chaotic Enforcement (CE). $CE_{i}^{j}$ represents the aggregated influence of randomly weighted components based on the chaotic map *w* in the *j*th dimension acting upon the *i*th agent and the best agent, formulated as follows:

(32) $$CE_{i}^{j}=w\times \left(X_{best}^{j}-X_{i}^{j}\right),\;\forall j\in \left[1,\mathrm{Dim}\right],$$

where $X_{best}^{j}$ is the position of the best agent, and the parameter *w* is the chaotic value in each iteration.

Intuitively, while the DP mechanism acts as a macro-diversity engine by evaluating the global feature layout via Hamming distances, the Chaotic Enforcement (CE) component functions as a fine-grained perturbation booster. The mathematical translation of the physical interaction force between binary solutions directly manifests as bit-flipping probabilities during the step-size initialization. Strong repulsion triggers higher mutation tendencies (flipping binary elements from 0→1 or 1→0), which physically forces stagnant agents to leave highly correlated feature paths and inspect unvisited dimensions of the binary hypercube. This programmatic coupling ensures that structural similarity is penalized dynamically, fostering high algorithmic diversity without expanding the runtime footprint.

### 3.3. Chaotic Newton–Raphson Search Rule (CNRSR)

The basic NRSR controls the solutions, which allows for a more accurate exploration of viable regions and better positioning. For more accurate results during the binary search, we aim to enhance both exploration and exploitation techniques during this phase by refining the exploitation through mathematical equation adaptations. The CNRSR basically depends on the Dynamic Potential and Chaotic Enforcement, as shown in Equations (31) and (32) respectively.

In the proposed BCNRBO, a new parameter called cρ is derived from Equation (7). This new parameter is responsible for the exploration and exploitation during the search process, and it is considered the main step of moving the new solution to the promising region. The fundamental factor that guides the population in the right direction is the Dynamic Potential and Chaotic Enforcement. From Equation (7), the parameters *a* and *b*, which are random numbers between (0, 1), have been replaced by the proposed parameters DP and CE respectively. The new chaotic ρ parameter can be referred to as the step size for each solution and is given as follows:

(33) $$c\rho^{i}=\mathrm{DP}\times \left(X_{best}-X_{i}\right)+\mathrm{DP}\times \left(CE_{i}-X_{RS}\right),$$

where DP is the Dynamic Potential from Equation (31), and the CE parameter is calculated from Equation (32). RS is a unique random integer from the range 1 to $N_{p}$. Each agent’s $c\rho_{i}$ value is checked to ensure it remains within the boundary of 0 and 1, preparing it for the transfer function in the next step.

**Figure 3:**
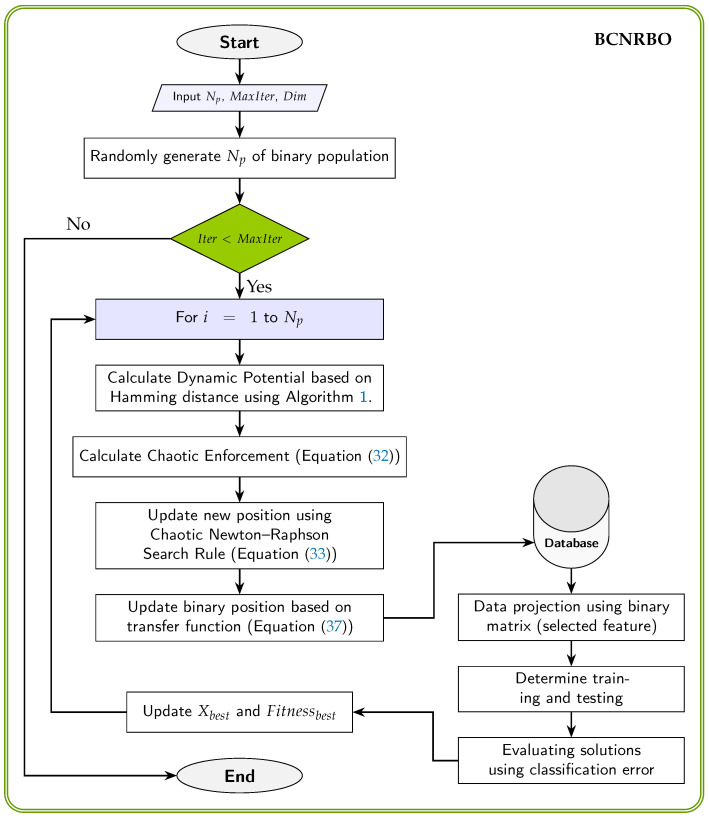
Flowchart of BCNRBO.

The iterative pseudocode steps of the pseudocode for the binary chaotic Newton–Raphson-Based Optimizer can be summarized as shown in Algorithm 2. The visual representation of the flowchart for the proposed algorithm, along with the binary process, is shown in Figure 3. The updating of the binary agents can be found in detail in the following subsection.
**Algorithm 2** Pseudocode of binary chaotic Newton–Raphson-Based Optimizer (BCNRBO)**Require:** $N_{p}$ (number of solutions), MaxIter (maximum iterations), Dim (dimension/number   of features), *w* (chaotic value) **Ensure:** $X_{best}$ and ${\mathrm{Fitness}}_{best}$  1:Randomly initialize binary population (Equation (22))  2:Iter←1  3:Calculate fitness for each binary solution $X_{i}$ ($i=1,2,\ldots ,N_{p}$)  4:**while** Iter≤MaxIter **do**  5:    **for** i=1 to $N_{p}$ **do**  6:        Calculate the Dynamic Potential by applying Algorithm 1  7:        Calculate the Chaotic Enforcement term ($CE_{i}^{j}$) using the chaotic map (Equation (32))  8:        Calculate the agent chaotic size cρ (Equation (33))  9:        Update the current binary agent using the transfer function (Equation (37))10:        Evaluate the new binary solution using the objective function11:        Update the current best $X_{best}$ and the best fitness ${\mathrm{Fitness}}_{best}$12:    **end for**13:    Iter←Iter+114:**end while**15:**return** $X_{best}$ (position of the best binary solution)


### 3.4. The Proposed Transfer Function

Utilizing transfer functions (TFs) is among the most straightforward techniques for converting a continuous form to a binary form. With the exception of a few minor adjustments to certain operators, they maintain the original continuous algorithm’s structure, which contributes to its simplicity. The probability that a continuous value in a solution will be mapped to a binary value is determined using TFs. Each solution is selected from the population and modified by incorporating information as explained previously.

In BCNRBO, the FS is represented as a binary vector with values of zero or one. A binary bit string of length Dim for a Dim-dimensional dataset represents the position of NRBO. While each bit represents a feature, a value of “1” means that feature is selected, and “0” means that the feature is not selected. Each position vector generated is a subset of the features generated from the original dataset.

In order to convert the current continuous solution to binary values bounded in the interval [0,1], the following new transfer function is suggested:

(34) $$S_{i}^{j}\left(Iter\right)=n\delta +\left|\tanh(\frac{\pi }{4}c\rho_{i}^{j}\left(Iter\right))\right|,\;\;S\in \left[0,1\right],$$

where cρ is the new agent chaotic size and is calculated using Equation (33). In the proposed transfer function, we introduce a new parameter nδ, which is derived from Equation (6) as follows:

(35) $$n\delta =1-\exp\left(\frac{-1}{MaxIter}\right).$$

The parameter nδ is a small positive constant (strictly bounded in the range [0,0.2]) introduced explicitly to mitigate the risk of zero-probability stagnation in the S-shaped transfer function. In standard formulations, since |tanh(·)| asymptotically approaches absolute zero when the continuous chaotic step size $c\rho_{i}^{j}$ is very small, the probability of executing a bit-flip freezes completely, causing the feature selection process to stall. By incorporating nδ, we mathematically guarantee a minimum baseline activation floor for $S_{i}^{j}\left(Iter\right)$.

**Figure 4:**
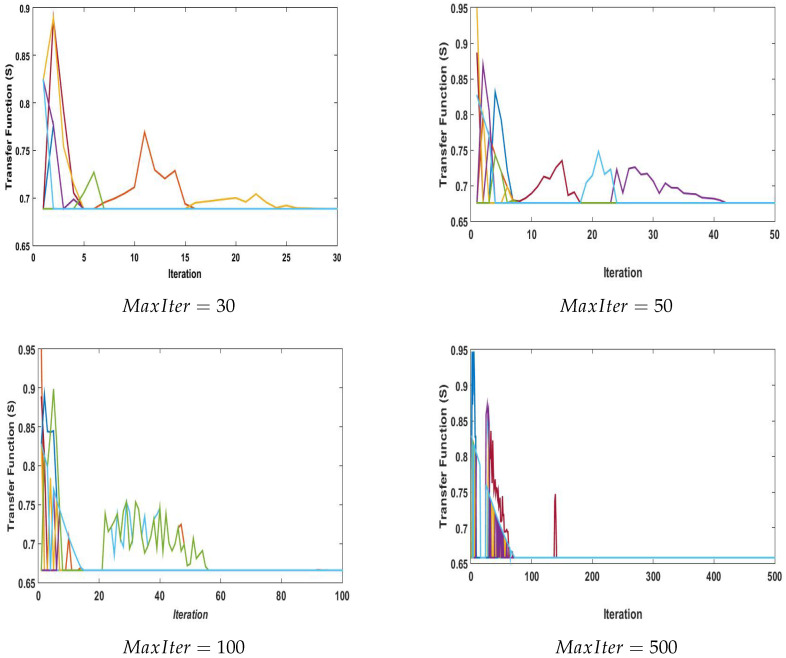
Visualization of the transfer function at different values of maximum iterations.

It is worth noting that the parameter nδ remains constant within a single run since it depends on the predefined maximum number of iterations (MaxIter); thus, its value adaptively scales, reflecting the overall search horizon of the optimization process, as visualized across different settings in Figure 4. The primary role of this threshold is to act as a probability bias that maintains a healthy, non-zero probability of stochastic bit-flipping within the binary hypercube. This effectively sustains exploration during critical search phases and avoids premature convergence without distorting the overall mathematical boundedness of the transfer function. The empirical necessity of this parameter is validated through the comprehensive macro-ablation contrast presented in Section 4.3 (specifically the w/o transfer function variant (omitting this baseline component, equivalent to the original BNRBO where nδ=0) results in a severe performance drop, shifting the accuracy from 96.00% down to 86.30%, thereby confirming that nδ prevents premature stagnation.

The obtained values of *S* are compared to random values, as shown in Equation (36), to update the new binary population.

The update of the binary solution $X_{i}^{\prime j}$ would be as follows:

(36) $$X_{i}^{\prime j}\left(Iter\right)=\left\{\begin{array}{cc} \mathrm{Complement}(X_{i}^{j}\left(Iter\right)), & \mathrm{if}\;rand<S_{i}^{j}\left(Iter\right)\\ X_{i}^{j}\left(Iter\right), & \mathrm{otherwise}. \end{array}\right.$$

After calculating the probabilities using the transfer function *S* and updating the binary solutions based on Equation (36), the final position updating equation is presented based on the current chaotic map value as follows:

(37) $$X_{i}^{\prime j}\left(Iter\right)=\left\{\begin{array}{cc} X_{best}^{j}, & \mathrm{if}\;w<rand\\ X_{i}^{j}\left(Iter\right), & \mathrm{otherwise}, \end{array}\right.$$

where *w* is the current chaotic value in the current iteration and rand is the random value between 0 and 1.

Evaluate the current solution and update the best-known solution if an improvement is found; then assess the current solution and revise the best solution accordingly.

Figure 4 illustrates the dynamic behavior of the transfer function $S_{i}^{j}\left(Iter\right)$ for multiple independent agents (represented by the different colored lines) across various maximum iteration limits (MaxIter=30, 50, 100,and500). Each colored line tracks the trajectory of a specific agent’s transfer function value over time. As visualized, despite the stochastic fluctuations and the chaotic step sizes leading to sharp drops, the curves never drop to absolute zero. Instead, they hit a stable baseline floor at the lower boundary, which is directly maintained by the constant nδ parameter. Furthermore, it is evident that as MaxIter scales up, the density of these exploration steps increases within the longer search horizon, yet the minimum activation floor remains firmly guaranteed, ensuring sustained stochastic bit-flipping and preventing premature stagnation throughout the entire optimization process.

### 3.5. Code Availability

The complete MATLAB implementation of BCNRBO and the custom scripts used to generate the results are publicly available at Zenodo: https://doi.org/10.5281/zenodo.18602047.

### 3.6. Computational Complexity

The computational complexity of the proposed BCNRBO is analyzed based on its core operations and compared to that of the original NRBO.

For the original NRBO, the complexity is calculated as $O(MaxIter\times N\times \left(N\times Dim+T_{fobj}\right))$. This result is obtained because the population mean is calculated inside the agent loop in the trap avoidance operator, requiring O(N×Dim) operations for each of the *N* agents in every iteration.

In the proposed BCNRBO, the computational complexity is derived based on the actual implementation steps. Unlike the original NRBO, the sorting operation is performed inside the main agent loop.

Specifically, for each agent in every iteration, the probability vector *P* is sorted in descending order. Since sorting a population of size *N* requires O(NlogN) operations and this process is repeated for all *N* agents, the total sorting cost per iteration becomes $O(N^{2}\log N)$.

Furthermore, the centroid of the $K_{best}$ individuals is recalculated inside the loop for each agent, which costs O(N×Dim) per agent, leading to $O(N^{2}\times Dim)$ per iteration.

The Hamming-distance computation between binary vectors of length Dim costs O(Dim) per agent. However, this term is dominated by the quadratic terms above.

The evaluation of the objective function for the entire population costs $O(N\times T_{fobj})$, where $T_{fobj}$ denotes the computational cost of evaluating the objective (fitness) function for a single agent.

By combining these steps, the total complexity of BCNRBO becomes

$$O\;\left(MaxIter\times \left(N^{2}\log N+N^{2}\times Dim+N\times T_{fobj}\right)\right).$$

By grouping the dominant quadratic terms, it can be expressed as

$$O\;\left(MaxIter\times \left(N^{2}\left(\log N+Dim\right)+N\times T_{fobj}\right)\right).$$

This analysis shows that the proposed BCNRBO preserves the same quadratic order with respect to the population size as the original NRBO. Although the inclusion of sorting operations introduces an additional $O(N^{2}\log N)$ term, the overall complexity remains dominated by the quadratic components $O(N^{2}\times Dim)$.

Moreover, the incorporation of Hamming-distance and chaotic-mapping mechanisms does not significantly increase the computational burden, since these operations scale linearly with respect to the population size and dimensionality. Regarding the solution time criterion: the computational complexity analysis above demonstrates that the proposed BCNRBO maintains the same quadratic order as the original NRBO. The additional operations (chaotic mapping, Dynamic Potential via the Hamming distance, and sorting) introduce a marginal constant-factor overhead rather than an asymptotic increase. In practice, the enhanced convergence behavior of BCNRBO leads to a reduction in the number of iterations required to reach an optimal or near-optimal solution compared to standard binary optimizers that suffer from premature convergence. Consequently, although each iteration may have a slightly higher constant cost, the overall algorithmic efficiency is significantly improved. This efficiency is empirically validated in the experimental results section through the convergence curves, where BCNRBO achieves better fitness values with fewer iterations, thereby demonstrating a highly effective search process without requiring an excessive number of algorithmic evaluations.

## 4. Computational Experiments

The suggested feature selection approach aims to determine the best feature subset that delivers optimal classification performance with the least number of features. Wrapper-based feature selection is used in this experiment with an objective function to evaluate the fitness of each solution. This framework makes use of the fitness function known as the classification error, which considers the following aspects:

(a) This study focuses on low to medium-dimensional large-scale datasets, including the PD Speech dataset with 753 features, to demonstrate the scalability of the proposed algorithm.
(b) This study simultaneously optimizes classification accuracy (minimum misclassification error) while minimizing selected features.

The BCNRBO technique has been tested on many datasets in the UCI repository [68], including low, medium, and large scales, employing four different classifiers:

▪ KNN classifier [19,69]: The K-nearest neighbor method is a popular classification method in data mining and statistics due to its simplicity and significant classification performance. It uses K-nearest neighbors to determine the class of examples, making it a memory-based classification and a lazy learning technique. The data were classified with k = 5 for KNN classification, and the training set results were obtained by dividing the data into training and testing sets. KNN is still utilized today, proving its accuracy and professionalism.
▪ DT classifier [70]: A decision tree is a tree-based technique used in data mining, where a path starts at the root and ends at the leaf node. They are hierarchical exemplifications of knowledge relationships, with nodes representing purposes. Decision trees are widely used in fields like machine learning, image processing, and pattern identification. They unify basic tests efficiently and cohesively, comparing numeric features to threshold values. They are commonly used for grouping purposes and classification models in data mining. Decision trees have found many implementation fields due to their simple analysis and precision on multiple data forms. In this paper, the decision tree classifier is implemented using the CART framework.
▪ NB classifier [71]: The Naive Bayes classifier is a simple yet effective approach based on the Bayesian theorem that is especially well suited for high-dimensional inputs. It is capable of outperforming more advanced classification algorithms. The prior probability, based on the percentage of Green and Red objects, is used to predict outcomes before they occur, ensuring that new cases are classified accordingly. It was derived from Bayesian Classification, which is a supervised learning and statistical method that uses probabilistic models to capture uncertainty and solve diagnostic and predictive problems.
▪ SVM classifier [72]: The Support Vector Machine (SVM) classifier is a powerful supervised learning algorithm used for classification and regression tasks. It works by finding the optimal hyperplane that maximizes the margin between different classes in a high-dimensional feature space. SVM is particularly effective in handling high-dimensional data and can model complex non-linear decision boundaries using kernel functions such as linear, polynomial, and radial basis function (RBF) kernels. Additionally, it is robust against overfitting, especially in cases where the number of features exceeds the number of samples. Due to its strong generalization ability, SVM has been widely applied in various fields, including bioinformatics, text classification, and medical diagnosis.

### 4.1. Algorithms, Parameters and Experimental Setup

Various datasets are used to assess the efficiency of the proposed algorithms. The proposed algorithm, along with other algorithms, was implemented using MATLAB R2022b on a PC with an Intel(R) Pentium(R) CPU running at 3.2 GHz and 32 GB of RAM. The parameter values of 20 and 50 have been utilized for the population size and maximum iterations, respectively.

The proposed algorithm is compared with state-of-the-art feature selection algorithms using the following criteria:

- Fitness values (using wrapper approach): They are obtained from each approach as reported (classification error: it is obtained by using the selected features on the test dataset). The mean, min, and max fitness values are compared. The average is calculated over 20 independent runs.
- Average selection features: It is the other comparison that has been presented here.
- *p*-value from Wilcoxon’s rank sum test and the mean rank value from the Friedman test (Wilcoxon’s rank sum test and the Friedman test are non-parametric statistical tests with a 5% significance level [73]).

The statistical test is necessary to show that the proposed algorithm provides a significant improvement compared to other algorithms. Here we are using two non-parametric tests: the Wilcoxon and Friedman rank tests [73]. Generally speaking, the best values of *p* are when *p* value <0.05. Therefore, it can be considered sufficient evidence against the null hypothesis.

The values for Wilcoxon’s test indicate that the null hypothesis *R* is rejected for “1”, and the proposed algorithm outperforms the other one.

A value of “−1” means the null hypothesis is rejected, and the proposed algorithm is inferior to the other one. A value of “0” reveals that the null hypothesis is accepted, and the proposed algorithm binds the other one. The tables report on the results obtained from both the Wilcoxon and Friedman rank tests, respectively, with a confidence level of 0.95 (α = 0.05).

The performance of the binary algorithm BCNRBO is compared with other optimization algorithms. These algorithms are proposed in the literature for binary optimization or for solving the feature selection problem. These algorithms are BAOA [40], BBA [41], BFPA [42], BPSO [43], BCCSA [31], jBASO [44] and BDA [32]. We added to the experimental test two more algorithms, the bee colony algorithm [74] and differential evolution [75]. These two algorithms were converted to binary versions (BABC and BDE, respectively) using the sigmoid function to demonstrate the effect of two powerful continuous algorithms on the binary problem. The parameter settings for all adopted optimization algorithms are taken from the original papers, as shown in Table 2.

### 4.2. Dataset Descriptions

The BCNRBO technique has been thoroughly evaluated using 26 benchmark datasets from well-established public repositories, including the UCI Machine Learning Repository [68], KEEL, Kaggle, and Arizona State University [76]. These datasets cover a variety of domains, primarily focusing on medical diagnosis, biological analysis, and general classification tasks.

As summarized in Table 3, the selected benchmarks represent diverse challenges with varying scales in terms of both sample counts and feature spaces. The dataset dimensions span from low-scale problems, such as Olive and Wisconsin, to multi-feature spaces, such as LSVT and PD Speech. This variation allows for a reliable assessment of the efficiency, adaptability, and scalability of the proposed feature selection algorithm within these targeted benchmarks.

For each dataset, the proposed algorithm was applied to select an optimal subset of features, which was then evaluated using four different classifiers: K-nearest neighbors, decision trees, and Naive Bayes (and further validated using the Support Vector Machine classifier). To ensure fair comparison and reduce randomness effects, each experiment was independently repeated multiple times, and the average classification accuracy was reported.

All datasets were preprocessed by normalizing feature values to a common scale before the optimization process. The same experimental settings, including the population size and the maximum number of iterations, were used for all algorithms to guarantee a fair and consistent comparison.

### 4.3. Ablation Study

To analyze the effectiveness of the proposed modifications in BCNRBO, an ablation study was conducted by evaluating the impact of each component individually. The study compares the original BNRBO algorithm with the full BCNRBO model, as well as three reduced variants where one component is removed at a time. Each experiment is conducted on Dataset 16, and the obtained results are reported in terms of fitness and classification accuracy.

From the results in Table 4, it is evident that the proposed BCNRBO outperforms the original BNRBO, achieving the best performance with an accuracy of 96% and the lowest fitness value, which confirms the effectiveness of the proposed enhancements.

A deeper analysis of the ablation variants reveals that the chaotic mechanism has the most significant impact on performance. When this component is removed, the accuracy drops to 81.74%, which is even lower than the original BNRBO. This clearly indicates that the chaotic strategy plays a crucial role in maintaining population diversity and preventing premature convergence.

In contrast, removing the Dynamic Potential leads to a moderate decrease in performance (84.78%), suggesting that it contributes to improving convergence behavior but is not the dominant factor.

Similarly, the transfer function also plays an important role, as its removal reduces the accuracy to 86.30%, highlighting its effectiveness in mapping continuous values into the binary search space.

Overall, the ablation study confirms that all components contribute positively to the performance improvement, with the chaotic mechanism being the most influential. The best results are achieved when all components are integrated together in the BCNRBO framework.

### 4.4. Evaluation and Analysis of the Proposed Algorithm Using Chaotic Maps

In the preliminary testing phase, ten various chaotic maps were tested to show which one of them would improve the convergence speed, local optima avoidance, and scalability of the BCNRBO algorithm. Ten different datasets with different sizes are used in this test. The shared datasets in this test are: Diabetes, Parkinsons, Diagnostic, Dermatology, Chess, Lung Cancer, Sonar, Hillvalley, LSVT, and PD Speech. See Table 3. It is noteworthy that KNN is used in this test with a population size of 20 over 20 runs and a maximum iteration of 50.

To demonstrate the effect of chaotic maps on the proposed method, ten different maps are integrated into the proposed algorithm. Furthermore, BNRBO represents an enhanced version of the proposed algorithm’s phases, but without the integration of chaotic maps.

Table 5, Table 6 and Table 7 present the performance results of BCNRBO utilizing various chaotic maps, alongside the enhanced binary BNRBO without chaotic map integration. Table 5 specifically summarizes the mean and standard deviation values for both variants. Note: Bold values in tables indicate the best (lowest) performance, representing the winning algorithm for each dataset.

The statistical analysis highlights Map10 (Tent map) as the most effective configuration, consistently outperforming others across multiple evaluation metrics. Notably, Map10 and Map7 each achieved top mean fitness value rankings on four datasets of differing dimensions. However, Map10 exhibited superior stability, recording the lowest standard deviation in eight out of ten benchmark datasets.

Furthermore, Map10 delivered the highest precision means for four specific datasets (Diabetes, Dermatology, Lung Cancer, and Sonar) and the most stable results (measured by standard deviation) across eight datasets, including Diabetes, Parkinsons, Diagnostic, Dermatology, Lung Cancer, Sonar, LSVT, and PD Speech. Comparatively, Map8 and the original BNRBO algorithm ranked second in stability, achieving optimal standard deviation values in five out of the ten evaluated datasets.

Similarly, BNRBO outperforms other algorithms when selecting the more significant features from the enormous available feature set.

To improve the reliability of the results, the Friedman test was used. The obtained results of this test are represented in Table 6, which confirms the previous analysis, demonstrating that the chaotic map Map10 outperforms its counterparts.

Based on the results of the Friedman test, Table 7 presents the *p*-values from the Wilcoxon signed-rank test comparing Map10 against its counterparts, along with their corresponding rank values (*R*).

The experimental findings demonstrate that integrating Map10 with the BNRBO algorithm significantly enhances its effectiveness in solving feature selection problems. Additionally, computational times across 10 different datasets were analyzed.

To provide a deeper mathematical insight into why Map10 (the Tent map) consistently outperforms the other chaotic maps across these statistical evaluations, it is essential to analyze their map-specific structural properties and their direct influence on binary optimization efficacy. The Tent map possesses a symmetric, piecewise linear structure that guarantees a perfectly uniform invariant probability density function over the interval (0,1). In a binary optimizer, where continuous chaotic variables are mapped via transfer functions to stochastic bit-flip probabilities, this uniform ergodicity ensures an unbiased, well-distributed exploration of the feature space. In contrast, alternative formulations, such as the Logistic map (Map1) or the Sinusoidal map, suffer from high structural density concentrations near the chaotic boundaries (clumping severely close to zero and one). This mathematical concentration forces the S-shaped transfer function to saturate frequently, biasing the algorithm toward either excessively flipping bits or freezing them entirely, which drastically accelerates premature convergence in complex feature dimensions. Furthermore, maps like the Gauss map can exhibit stabilizing orbits or narrower chaotic bands, depending on the initialization seeds, leading to the inconsistent ranking fluctuations observed across different datasets in Table 6. Consequently, the uniform distribution of the Tent map provides the optimal exploration–exploitation balance required to navigate high-dimensional feature selection masks effectively.

Overall, the results confirm that incorporating chaotic maps into the binary optimizer improves performance, with the original BNRBO (without chaotic maps) showing comparatively lower effectiveness. Among all tested chaotic map variants, the Tent map emerged as the most effective, consistently delivering superior results.

## 5. Experimental Results and Discussion

The effectiveness of the proposed algorithms is evaluated across 26 different datasets, with details provided in Table 3. To obtain statistically meaningful results, every algorithm is run independently 20 times. Moreover, for all proposed algorithms, the size of the population and the number of iterations are set to 20 and 50, respectively, over 20 independent runs.

For the classification and fitness evaluation process, a rigorous Hold-Out validation protocol was systematically enforced to eliminate any risk of data leakage or overfitting (which are common challenges in wrapper-based feature selection). In each independent run, the dataset was randomly partitioned into two mutually exclusive subsets: 80% of the data was assigned via validation masks as the training subset to fit the classification models, while the remaining 20% was strictly reserved as an independent testing subset. This unseen testing partition serves as an external validation set to assess true generalization performance. This random partitioning was dynamically repeated across all 20 independent runs to minimize structural bias, ensure that the wrapper optimization targets genuine generalization boundaries, and guarantee that the reported performance reflects true predictive capacity rather than accidental data pattern exploitation. The multi-faceted evaluation framework initially utilized three diverse learning algorithms: KNN, DT and NB classifiers. In the results presented in tables of the respective classifiers, we conducted rigorous statistical analyses, including: (1) descriptive statistics (the best, worst, mean, standard deviation of fitness values, and the average number of selected features); (2) the non-parametric Friedman test for overall performance ranking across all datasets; and (3) pairwise Wilcoxon signed-rank tests for detailed comparisons with state-of-the-art algorithms. We provide additional results for the Support Vector Machine (SVM) learning method, which is considered a stronger learning algorithm, in a separate Section 5.4 (see tables for SVM results).

The best results across all tables are highlighted in bold to underscore their significance relative to the other values.

### 5.1. Evaluation of BCNRBO with KNN Classifier

In this section, we present the results obtained by the proposed algorithm alongside those from other benchmark algorithms using the KNN classifier, serving as the first experiment in our evaluation. The purpose of this experiment is to assess and compare the classification performance of each algorithm based on selected features.

The primary objective of optimization algorithms is to minimize the fitness function, thereby identifying the most optimal solution to a given problem. As illustrated in Table 8, Table 9, Table 10, Table 11, Table A2 and Table A3, the proposed BCNRBO algorithm consistently achieved better solutions across most evaluated datasets in terms of the min, mean, worst, and std fitness values, respectively.

Table 8 presents the best fitness values obtained using the KNN classifier across 26 datasets. The BBA algorithm shows the best overall performance, achieving the top result on 10 datasets. The BDA algorithm ranks second with nine best results, followed by BCNRBO with eight. The BABC and jBASO algorithms each performed best on seven datasets.

Table 9 presents the average fitness values obtained using the KNN classifier, revealing that the BCNRBO algorithm demonstrates superior consistency by achieving the best average performance on 10 out of 26 datasets. Both BABC and BPSO algorithms follow with seven best average results each. Both BDE and BDA algorithms secured five best averages, while jBASO, BBA, and BCCSA each obtained four. Finally, BAOA and BFPA achieved the best results on three and two datasets, respectively.

As is evident from Table A2 the worst (maximum) fitness values obtained using the KNN classifier are presented. The BCNRBO algorithm demonstrates the best efficiency, achieving the lowest worst-case error on nine datasets. BABC, BPSO, and BDA follow with strong performance, each securing the best worst-case result on eight datasets. BAOA and BDE each performed best on six datasets.

Table A3 presents the standard deviation of fitness values obtained using the KNN classifier. The BPSO algorithm shows the highest consistency, achieving the lowest standard deviation on 12 datasets. Both BCNRBO and BABC follow closely with strong stability, each securing the most consistent results on 10 datasets. The BDA algorithm achieved the best results on eight datasets, followed by BFPA and BDE with seven datasets each, while jBASO and BBA each obtained six. Finally, BAOA and BCCSA demonstrated the lowest standard deviation on five and three datasets, respectively. This indicates that BPSO, BCNRBO, and BABC produce the most reliable and repeatable performance when used with the KNN classifier.

In addition, Table A4 reports the average number of selected features obtained by the proposed binary algorithm BCNRBO and other algorithms. Similar to the classification error and fitness values, the number of selected features is a significant metric to evaluate the effectiveness of feature selection methods tested by the KNN classifier. The classification model was implemented using the KNN classifier in MATLAB (fitcknn) with five nearest neighbors (k = 5).

The BCNRBO algorithm achieves the most compact feature subsets, selecting the fewest features on 14 out of 26 datasets. The jBASO algorithm follows, performing best on seven datasets, while the BAOA algorithm secures the best results on two datasets. Finally, BABC, BPSO, and BDA each achieve the most compact feature count on a single dataset. This indicates that BCNRBO is the most effective at identifying minimal feature subsets while maintaining classification performance with KNN.

#### 5.1.1. Statistical Test for KNN Classifier

The superior performance of BCNRBO comes from its dynamic switching mechanism between 0 s and 1 s (or vice versa), guided by the proposed Dynamic Potential, Chaotic Enforcement and proposed transfer function techniques. This approach effectively compensates for the lack of prior experiential knowledge in the solutions, ensuring robust and adaptive performance.

The Friedman test results presented in Table 10 provide statistically rigorous evidence for performance comparisons among algorithms using the K-nearest neighbors classifier across 26 datasets.

BCNRBO demonstrates statistical superiority by achieving the lowest average rank of 4.227 in Table 10, followed by BDA with an average rank of 4.575 and BBA with 5.083. In contrast, BCCSA shows the highest average rank of 7.048, indicating statistically weaker performance.

Examining the specific datasets where BCNRBO achieved the top rank reveals its consistent performance across diverse data characteristics. BCNRBO secured the first position in 10 out of 26 datasets, including D1, D5, D6, D9, D11, D17, D20, D23, D24, and D26. This distribution represents the highest success rate among all evaluated algorithms, further validating the mathematical significance of the proposed method.

To assess the statistical significance of BCNRBO’s performance, the Wilcoxon signed-rank test results presented in Table 11 provide strong pairwise statistical confirmation of the Friedman test findings for the KNN classifier. The test demonstrates that BCNRBO significantly outperformed most competing algorithms.

Specifically, BCNRBO achieved its most dominant performance against BFPA and BCCSA, significantly outperforming them on 19 and 18 datasets, respectively, followed by BDE (16 wins) and jBASO (15 wins). On the other hand, BBA and BPSO proved to be the most competitive counterparts; BCNRBO recorded its fewest wins against BBA (12 wins) and experienced its lowest number of significant differences with both algorithms, resulting in the highest number of statistical ties (10 equal results each). Moreover, BCNRBO maintained a positive win-to-loss ratio against all evaluated peer algorithms across the 26 datasets, mathematically demonstrating its superior and stable performance under the KNN classifier.

#### 5.1.2. Convergence Graphic for Different Datasets Using KNN Classifier

The convergence characteristic curves of the basic BCNRBO on the different dimensions of 15 datasets are studied using the KNN classifier. The four weakest performing algorithms were removed from the graph to improve explanation, and their weak performance was statistically confirmed based on the Friedman test results (Table 10).

**Figure 5:**
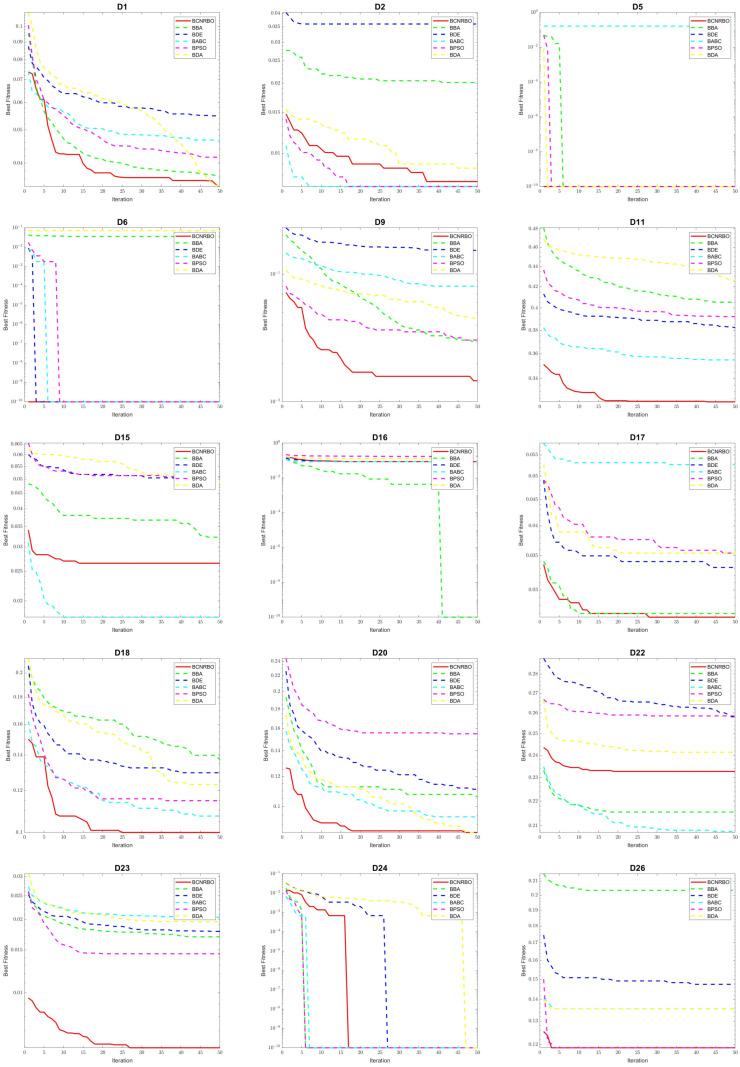
Convergence history for different datasets using KNN test.

According to Figure 5, the convergence behavior of the competing algorithms over 15 benchmark functions with different dimensionalities is clearly illustrated. For clarity and better visualization, the four weakest-performing algorithms were excluded from the comparison.

As shown in the figures, the proposed BCNRBO algorithm achieves the best fitness values on functions D1, D9, D11, D17, D20, D23, and D26, demonstrating its strong capability to balance exploration and exploitation during the search process. In addition, BCNRBO exhibits competitive performance by attaining comparable results on functions D5, D6, and D24, indicating stable and consistent convergence behavior across different problem landscapes.

It is also observed that some competing algorithms, including jBASO, BBA, BFPA, and BABC, achieve lower fitness values on a limited number of functions. This outcome highlights the problem-dependent nature of metaheuristic optimization algorithms. Nevertheless, the overall results confirm the superiority and efficiency of the proposed BCNRBO algorithm when compared with the other binary optimization methods.

### 5.2. Evaluation of BCNRBO with DT Classifier

This section presents the performance of the proposed algorithm against benchmark methods using the DT classifier in our second experiment. The goal is to evaluate and compare the classification error of each algorithm on selected feature subsets. The experimental results demonstrate the superior capability of the proposed BCNRBO algorithm, as evidenced by the comprehensive evaluation in Table 12, Table 13, Table 14, Table 15, Table A6 and Table A6.

The statistical analysis further validates that the proposed algorithm consistently outperforms competing methods in terms of reducing classification error. Table 13 and Table A6 present the mean and standard deviation of the fitness values achieved by the proposed binary chaotic NRBO algorithm. Since lower fitness values reflect better performance, these metrics serve as key indicators of algorithmic effectiveness.

Table 12 presents the best fitness values obtained using the DT classifier across 26 datasets. The bolded values indicate the best performance (lowest fitness value) for each dataset.

From the analysis of the results, the BCNRBO algorithm demonstrates the most superior optimization performance, achieving the best fitness values on 10 datasets (D1, D2, D3, D6, D8, D12, D13, D14, D20, and D24). The BBA algorithm follows as the second most effective competitor, excelling on eight datasets, while the BABC algorithm exhibits a competitive performance by securing the top rank on seven datasets. Additionally, BDE and BPSO achieve the best results on six datasets each, followed closely by jBASO and BDA with five datasets each. Finally, BAOA and BCCSA perform best on four datasets each, while BFPA records the lowest fitness on two datasets. This highlights the high capacity of BCNRBO in finding the optimal or near-optimal solutions when integrated with the DT classifier.

As illustrated in Table 13, the average fitness values obtained using the DT classifier across 26 datasets provide insights into the consistent performance of each algorithm.

The proposed BCNRBO algorithm maintains its leading position, achieving the most stable and superior performance by securing the lowest average fitness values on eight datasets (D2, D3, D6, D13, D14, D15, D24, and D25). Both BBA and BABC emerge as the closest competitors, each achieving the best average results on five datasets. These are closely followed by BDA and BPSO, which record the lowest average fitness on four datasets each. Furthermore, jBASO and BDE demonstrate localized effectiveness, securing the best performance on two datasets each, while BAOA leads in only a single dataset (D13). In contrast, BFPA and BCCSA do not secure the best average fitness on any dataset. These findings indicate that BCNRBO delivers highly competitive and dependable average classification accuracy when coupled with the DT classifier.

Table A5 presents the worst fitness values obtained using the DT classifier across the 26 datasets. This analysis of worst-case performance provides valuable insights into algorithm efficiency and stability.

BCNRBO demonstrates exceptional reliability by achieving the best (lowest) worst-case performance in 13 out of 26 datasets (D2, D3, D6, D8, D9, D13, D14, D15, D20, D22, D23, D24, and D25), substantially outperforming all other algorithms. The BABC algorithm emerges as the second most stable competitor, securing the lowest worst-case fitness on eight datasets, followed closely by BPSO with six datasets and BBA with five datasets. Additionally, both BDE and BDA demonstrate moderate stability by achieving the best results on three datasets each, while BAOA and jBASO each lead in only one dataset. In contrast, BFPA and BCCSA fail to record the best worst-case performance on any dataset.

These results reveal an important dimension of algorithm performance. While several algorithms can achieve good results under optimal conditions, BCNRBO maintains superior performance even in worst-case scenarios, mathematically confirming its resistance to getting trapped in poor local optima and establishing its high algorithmic stability under the DT classifier.

Table A6 presents the standard deviation of fitness values obtained using the DT classifier across 26 datasets. Standard deviation measures the consistency and stability of algorithm performance, with lower values indicating more reliable results.

In this analysis, the BABC algorithm demonstrates the highest stability, achieving the lowest standard deviation across 11 datasets, followed by BDE, which secures the top stability ranking in seven datasets. The proposed BCNRBO algorithm exhibits highly dependable consistency by achieving the lowest standard deviation in six datasets (D2, D4, D6, D13, D19, and D24). Among the remaining benchmark methods, BFPA and BPSO show competitive stability in five datasets each, followed by BDA in four datasets, while both jBASO and BBA achieve the lowest deviations in three datasets each. Finally, BAOA and BCCSA register the lowest values in only two datasets each. It is worth noting that several algorithms recorded standard deviations in the scale of $10^{-17}$ (such as in D5, D6, D16, and D26), which computationally represents absolute stability equivalent to zero due to machine precision limitations.

These results provide an important perspective on algorithm reliability. While BDE and BABC deliver localized superior consistency in terms of standard deviation, BCNRBO offers a more balanced and robust trade-off; it provides the absolute best optimization capability across best, average, and worst fitness values while maintaining a highly competitive and narrow variance profile under the DT classifier.

Table A7 presents the average number of selected features obtained using the DT classifier across 26 datasets. This metric is crucial for evaluating algorithm efficiency in feature selection, where fewer selected features generally indicate better dimensionality reduction while maintaining classification performance.

The experimental results reveal that the proposed BCNRBO algorithm demonstrates remarkable efficiency and superior parsimony, selecting the smallest subset of features in 15 out of 26 datasets (D3, D5, D6, D8, D9, D10, D13, D14, D15, D16, D18, D19, D20, D21, and D24). This performance significantly surpasses all other benchmark methods, confirming its exceptional capability in eliminating redundant and irrelevant data. Following at a distance, the jBASO algorithm achieves the minimal feature count in six datasets, showcasing moderate efficiency in dimensionality reduction. Additionally, both BAOA and BABC exhibit localized advantages by capturing the minimum feature counts in two datasets each, while BBA leads in only a single dataset (D7). Conversely, benchmark algorithms, such as BFPA, BCCSA, BDE, BPSO, and BDA, fail to achieve the best feature reduction on any of the evaluated datasets.

#### 5.2.1. Statistical Test for DT Classifier

The experimental results revealed statistically significant performance improvements over all comparative methods, as quantified through the classification error. These findings validate BCNRBO’s enhanced exploration–exploitation balance and its capability to overcome local optima traps common in high-dimensional search spaces.

To rigorously verify the statistical significance of these improvements, a Friedman alignment test was conducted. Table 14 and Table 15 present the statistical outcomes, including the mean ranks (*R*) and subsequent post hoc *p*-values comparing the proposed BCNRBO against the nine benchmark metaheuristics (BAOA, jBASO, BFPA, BBA, BCCSA, BDE, BABC, BPSO, and BDA) under the DT classifier framework.

The Friedman test results presented in Table 14 provide robust statistical evidence for performance comparisons using the DT classifier across 26 datasets. The empirical analysis presented in Table 14 demonstrates that the proposed BCNRBO algorithm successfully secures the first overall rank with the lowest global average rank of 4.206 and a total summation rank of 109.350. Individually, BCNRBO attains the absolute best rank across eight datasets, namely D2, D3, D6, D13, D14, D15, D24, and D25. Statistically, the BABC algorithm occupies the second position with an average rank of 4.654, followed closely by BDA in third place with an average rank of 4.677 and BBA in fourth place with a mean rank of 4.966. Conversely, the BCCSA algorithm demonstrates the weakest overall optimization capability, landing in the final position with a substantially higher average rank of 8.150. This clear statistical separation firmly substantiates the mathematical superiority of BCNRBO over the competing benchmark methods.

The Wilcoxon signed-rank test results presented in Table 15 provide robust pairwise statistical confirmation of the Friedman test findings for the DT classifier. The significance is evaluated at a standard threshold of α=0.05, where a *p*-value of less than 0.05 indicates a statistically significant performance difference between the two compared algorithms. The test demonstrates that BCNRBO significantly outperformed all competing algorithms across the majority of the 26 datasets. Specifically, BCNRBO achieved its most dominant performance against BCCSA, securing a significant victory in 22 datasets, followed by BAOA and BFPA, where BCNRBO was victorious in 17 datasets each. It also maintained a clear upper hand over jBASO, BDE, and BPSO, registering 16 wins against each. On the other hand, BBA and BDA emerged as the most competitive counterparts. BCNRBO secured 13 and 11 significant wins against BBA and BDA, respectively, while both benchmarks demonstrated high resilience by extracting eight statistical ties each and limiting BCNRBO to its lowest winning margins.

Nevertheless, since the number of significant wins (Won) by BCNRBO vastly outnumbers its losses across all pairwise pillars, these outcomes statistically reject the null hypothesis and confirm the definitive superiority of the proposed BCNRBO algorithm under the DT classifier framework.

#### 5.2.2. Convergence Graphic for Different Datasets Using DT Classifier

**Figure 6:**
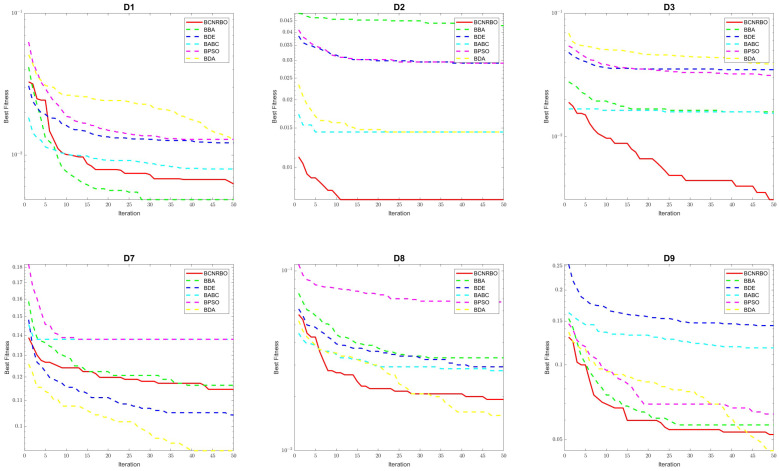

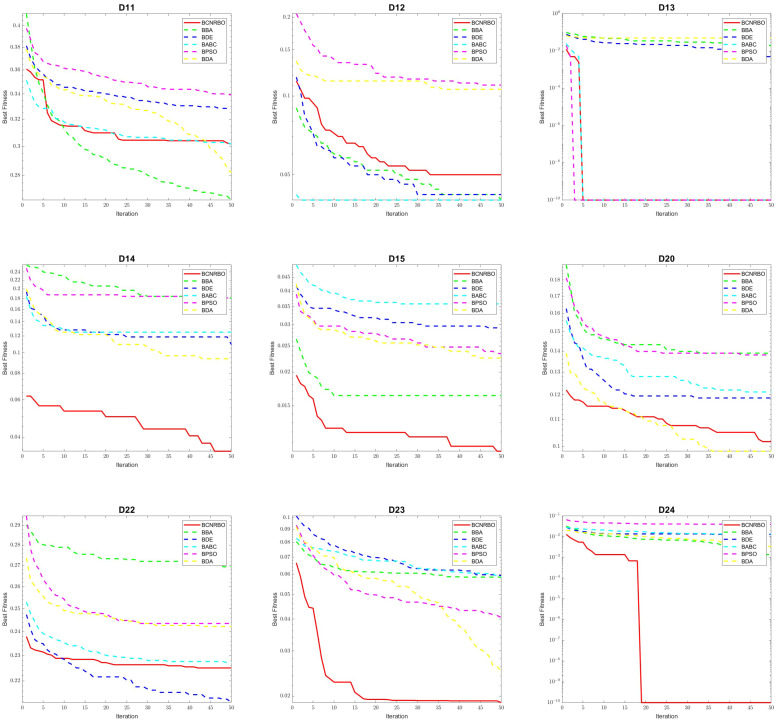
Convergence history for different datasets using DT test.

The convergence behavior of BCNRBO was analyzed using the DT classifier. For clarity, 15 representative datasets were selected from the 26 benchmark datasets. Figure 6 shows that BCNRBO achieves the lowest fitness values in nine cases (F2, F3, F8, F9, F14, F15, F20, F23, and F24), demonstrating its strong optimization capability under the DT framework.

Competing algorithms, such as BBA, BDA, BABC, and BPSO, show competitive performance on select cases, while weaker methods were omitted for clarity. One can see that BCNRBO exhibits stable and consistent convergence, outperforming other binary optimizers in final solution quality.

### 5.3. Evaluation of BCNRBO with NB Classifier

In this section, the third and final experiment compares the proposed BCNRBO algorithm with benchmark methods using the NB classifier. Datasets are also split into 80% training and 20% testing sets, and the quality of selected feature subsets is evaluated using the fitness, mean, standard deviation, and number of selected features (Table 16 and Table A9). The results highlight BCNRBO stability and effectiveness in different dimensional search spaces, demonstrating consistent optimal performance.

Table 16 presents the NB classifier results, showing a distinct performance pattern compared to DT. BCNRBO achieves the best (lowest) fitness values in 13 out of 26 datasets, confirming its strong optimization capability across different classifier settings. Among competitors, BDA ranks second with top performance in nine datasets, followed by jBASO and BPSO with eight datasets each and BBA with seven datasets. BFPA and BDE achieve best results in six datasets each, while BAOA, BCCSA, and BABC each lead in four datasets. In some cases (e.g., D5 and D6), multiple algorithms reach zero fitness, indicating perfect convergence in those search spaces.

Table 17 reports average fitness values under the NB classifier, highlighting algorithm consistency. BCNRBO consistently achieves the lowest mean fitness across 9 out of 26 datasets, demonstrating reliable optimization beyond stochastic effects. BPSO follows with strong secondary stability in six datasets each, while BFPA, BBA, and BDE show moderate consistency in five datasets each. BAOA, BABC, and BDA achieve localized success, and BCCSA and jBASO perform the weakest.

Table A8 evaluates the worst-case performance under the NB classifier. BCNRBO achieves the best worst-case (lowest upper-bound fitness) performance in 10 datasets, demonstrating strong resilience against unfavorable initialization and optimization degradation. Among competitors, BPSO and BDE follow with strong performance, while BFPA, BBA, and BDA show moderate effectiveness. BABC and BAOA achieve limited success, whereas jBASO and BCCSA perform the weakest in this regard. Overall, BCNRBO consistently limits worst-case errors, confirming its strong balance between exploration and exploitation and its reliability in high-dimensional feature selection problems.

Table A9 presents the standard deviation analysis under the NB classifier, reflecting algorithm stability. BDE and BPSO show the highest consistency, followed by BABC. BCNRBO demonstrates strong and reliable stability, matching BFPA by achieving low variance across several datasets, including zero variance in some cases. In contrast, BBA, BAOA, and BDA achieve moderate stability, while jBASO and BCCSA show no top performance.

Table A10 reports the average number of features selected using the NB classifier. Minimizing feature subset size while maintaining classification performance is a key measure of algorithm effectiveness. As shown in the table, BCNRBO demonstrates strong data reduction, achieving the smallest feature sets in over half of the evaluated datasets. The jBASO algorithm ranks second in feature reduction, while BCCSA, BAOA, BFPA, and BPSO each achieve isolated wins. Overall, BCNRBO excels at removing irrelevant and redundant features under the NB classifier, producing compact subsets that improve both computational efficiency and model interpretability.

#### 5.3.1. Statistical Test for NB Classifier

The Friedman test results presented in Table 18 provide robust statistical validation for the performance comparisons under the NB classifier across the 26 datasets. The empirical outcomes demonstrate that the proposed BCNRBO algorithm successfully secures the definitive first overall position, achieving the lowest global average rank of 3.949 and a minimum cumulative summation rank of 102.675.

On individual datasets, BCNRBO consistently attains the top rank, followed by BBA, BPSO, and BDA in descending order. Other competitors trail behind, with BCCSA showing the lowest optimization stability. These results highlight BCNRBO’s superior balance between exploitation and exploration and its efficiency across diverse search spaces.

To establish robust pairwise statistical confirmation of the Friedman test findings, a Wilcoxon signed-rank test was executed under the NB classifier framework. As presented in Table 19, the significance is evaluated at a standard threshold of α=0.05, where a *p*-value <0.05 denotes a statistically meaningful performance deviation between BCNRBO and its competitors.

The empirical metrics confirm that the proposed BCNRBO significantly outperforms all nine benchmark algorithms across the vast majority of the 26 datasets. BCNRBO registers its most dominant behavior against BCCSA with 21 significant wins, followed closely by BAOA and BDE with 19 wins each and BFPA with 18 wins. It maintains a distinct statistical advantage over jBASO, BABC, and BPSO, securing 17 victories against each. Conversely, BBA and BDA emerge as the most resilient counterparts. BCNRBO secures 15 significant wins against BBA, while BDA limits BCNRBO to its lowest winning margin of 11 victories by extracting a notable maximum of eight statistical ties and committing only seven losses. Crucially, BCNRBO achieves substantially more wins than losses across all pairwise comparisons, leading to a clear rejection of the null hypothesis. This confirms its statistical superiority and effectiveness under the NB classifier framework.

#### 5.3.2. Convergence Graphic for Different Datasets Using NB Classifier

This section presents the convergence characteristics of the proposed BCNRBO algorithm across fifteen benchmark datasets with varying dimensionalities, using the NB classifier for performance evaluation.

**Figure 7:**
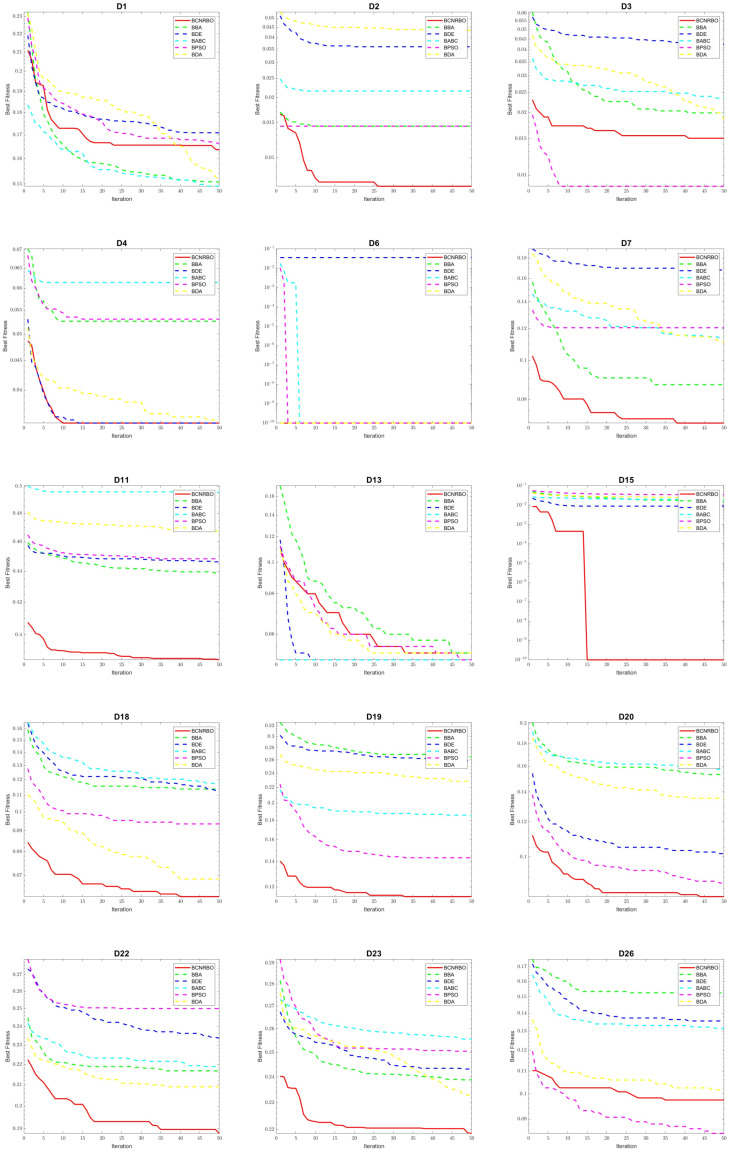
Convergence curves of BCNRBO and competing classifiers on different datasets using the NB test.

Figure 7 illustrates the convergence performance of the competing binary optimization algorithms using the NB classifier. As depicted in these sub-figures, the proposed BCNRBO algorithm successfully achieves the lowest fitness values in 10 out of the 15 benchmark functions. In addition, it is observed that some competing algorithms, including BABC, BBA, and BPSO, are capable of reaching the global minimum in a limited number of functions, highlighting their competitive optimization behaviors in specific cases. Nevertheless, the overall convergence trajectories confirm that BCNRBO consistently outperforms the other counterpart metaheuristics in terms of solution quality and convergence stability when integrated with the NB classifier framework.

### 5.4. Evaluation of BCNRBO with SVM Classifier

This section presents an additional experiment to evaluate the performance of the proposed BCNRBO algorithm using the Support Vector Machine classifier. The primary objective is to compare the effectiveness of the proposed method against selected benchmark optimization algorithms in terms of classification accuracy and feature selection capability. In this experiment, the datasets were randomly divided into training (80%) and testing (20%) subsets.

Unlike previous experiments, only the best-performing benchmark algorithms from the earlier comparisons (KNN, DT, and NB classifiers) were selected for this evaluation rather than including all algorithms. The selection was conducted based on their performance across the benchmark functions listed in Table 3. This strategy ensures a more focused and rigorous comparison, aiming to demonstrate the superiority of the proposed BCNRBO algorithm against the most competitive methods.

To ensure a comprehensive assessment, several performance metrics were considered, including fitness values, the mean, the standard deviation, and the number of selected features. These metrics provide insights into both the accuracy and stability of the optimization process. Furthermore, to rigorously analyze the optimization behavior of BCNRBO, we report the mean fitness values obtained over multiple independent runs, as shown in Table 20. Table 21 shows the obtained results of the Friedman test.

The training and testing indices are fixed and reused across all algorithms to ensure fair and consistent evaluation. A SVM classifier is trained on the training set and evaluated on the test set using the misclassification rate. The fitness function combines the classification error and the proportion of selected features to balance accuracy and feature reduction. Specifically:

$$\mathrm{Fitness}=\omega \cdot \mathrm{Error}\;\mathrm{Rate}+\left(1-\omega \right)\cdot \left(\frac{\mathrm{Number}\;\mathrm{of}\;\mathrm{Selected}\;\mathrm{Features}}{\mathrm{Total}\;\mathrm{Features}}\right),\;\omega =0.99.$$

This formulation prioritizes classification accuracy while still penalizing large feature subsets to encourage dimensionality reduction. By assigning a dominant weight to ω, the fitness function ensures that the optimization process strictly guards against any degradation in predictive performance, which remains the primary objective in most critical classification tasks. Meanwhile, the minor weight given to the feature reduction component (1−ω=0.01) functions effectively as a penalty factor or a tie-breaker, guiding the algorithm to select the most parsimonious feature subset when faced with identical classification error rates. This method provides a reliable framework for evaluating the trade-off between feature reduction and classification performance across different datasets and feature selection algorithms [9,19,77].

The empirical data clearly demonstrates the outstanding superiority of the proposed BCNRBO algorithm (Table 20), which dominates the benchmark suite by securing the best average fitness in 16 out of 26 datasets (specifically on D2, D3, D4, D6, D7, D8, D10, D13, D15, D16, D18, D19, D21, D22, D23, and D26). This extensive dominance underscores the exceptional balance that BCNRBO maintains between exploration and exploitation, ensuring high stability and a consistent ability to escape local optima.

Conversely, the jBASO algorithm shows a noticeable decline in its general performance compared to the best fitness results, securing the lowest average values in nine datasets (D1, D5, D9, D11, D12, D17, D20, D24, and D25). This indicates that while jBASO can find high-quality solutions in its best runs, its convergence behavior is less stable than that of the proposed method over multiple trials.

Table 22 demonstrates that the proposed BCNRBO algorithm achieves competitive feature reduction capabilities, securing the lowest average number of selected features in 12 out of 26 datasets. It stands as the overall best performer, followed closely by jBASO with nine wins, while BAOA (three wins) and BCCSA (one win) exhibit more localized efficacy.

Table 21 outlines the statistical results of the Friedman test. The statistical indicators clearly validate the exceptional efficiency of the proposed BCNRBO algorithm, as it achieves the lowest overall average rank of 1.9538 and the minimum summation of ranks (50.8) across the 26 benchmark datasets. In terms of localized dominance (indicated by bold values), BCNRBO secures the first rank in 17 out of 26 datasets. Statistically, the clear margin by which BCNRBO minimizes both the average rank and rank summation solidifies its position as the most effective and stable optimization method among all evaluated algorithms for tuning the SVM classifier.

## 6. Conclusions and Future Work

In this study, a novel binary chaotic Newton–Raphson-Based Optimizer was proposed for solving the feature selection problem. The algorithm introduces a binary extension of NRBO enhanced with various chaotic maps to improve exploration in binary search spaces.

A Dynamic Potential mechanism inspired by the Lennard-Jones potential was incorporated, where the Hamming distance was integrated into DP to measure interactions between binary solutions. The Tent chaotic map is integrated into the proposed algorithm to enhance exploration and maintain solution diversity. Its chaotic sequences are used in the transfer function and to adaptively update the step size of each agent (Chaotic Enforcement), enabling a more dynamic and effective search in the binary feature selection space.

The performance of BCNRBO was evaluated using 26 benchmark datasets of varying sizes (low, medium, and large scale), including medical datasets. Three classifiers, K-nearest neighbors, decision trees, and Naïve Bayes, were used to assess classification accuracy. Experimental results demonstrated that BCNRBO consistently outperforms several state-of-the-art metaheuristic algorithms, including BAOA, BBA, BFPA, BPSO, BCCSA, jBASO, and BDA, in terms of classification accuracy, fitness value, and feature subset reduction. The superiority of the proposed method was further confirmed through statistical tests.

These results highlight several key contributions and developments:

- The introduction of a binary chaotic NRBO variant capable of effectively handling discrete feature selection problems.
- The incorporation of a Dynamic Potential mechanism to enhance population diversity and improve exploration–exploitation balance.
- The development of a transfer function mechanism to improve the conversion of continuous solutions into binary space.
- Demonstration of the superior performance of BCNRBO across multiple datasets, classifiers, and benchmark algorithms.

Despite the promising results, certain limitations of the proposed BCNRBO method should be acknowledged. First, the inclusion of Chaotic Enforcement alongside the Dynamic Potential mechanism based on the Hamming distance increases the internal computational overhead per iteration, which may affect its scalability when applied to ultra-high-dimensional datasets with hundreds of thousands of features. Second, like most metaheuristic algorithms, the performance of BCNRBO can be sensitive to the selection of initial hyperDs, such as the population size and the choice of the chaotic map, requiring a degree of trial-and-error for specific dataset structures.

For future work, the proposed algorithm can be extended to multi-objective optimization settings and further enhanced through hybridization with other metaheuristic techniques, adaptive parameter control, and application to large-scale real-world feature selection tasks. Additionally, exploring other chaotic maps and dynamic transfer functions could provide further improvements in search efficiency and solution quality.

## Figures and Tables

**Table 1:** Detailed comparison of selected metaheuristic feature selection algorithms.

Algorithm	Dataset(s)	Class	Mechanism	Chaos	Main Notes	Limitations
BPO [53]	Benchmark (Knapsack)	N/A	Sigmoid and probabilistic	No	Strong exploitation via puma hunting.	Limited validation on feature selection datasets.
BAOA [54]	SCP datasets	N/A	S-shaped and V-shaped TFs	No	Analysis of transfer function impact.	Not validated on medical datasets.
CDMO [55]	UCI datasets	KNN	Chaotic map thresholding	Yes	Improves search over standard DMO.	Sensitivity to initial chaotic parameters.
CBBOA [56]	ASD/ classification	NB, KNN	Chaos-based transfer	Yes	Enhances classification accuracy.	Risk of local optima in complex data.
BGOA [57]	UCI, DEAP	KNN	Gaussian TF	No	Strong global search and fast convergence.	Needs careful tuning of parameters.
BHOA [58]	Microarray datasets	SVM	X-shape TF	No	Hybrid MRMR improves gene selection.	Dependency on the filter-based stage.
BWaOA [59]	High-dim UCI	KNN	Crossover update	No	Improves convergence quality and speed.	Increased computational overhead.
BSMO [60]	Medical datasets	KNN, SVM	S-shaped transfer	No	Models collective bird behavior.	Premature convergence risk; needs tuning.
BRSA [61]	Benchmark, UCI	KNN, SVM	S/V-shaped TFs	No	Strong exploration and exploitation.	Performance may degrade in high-dim spaces.

**Table 2:** Parameter Settings of the Compared Algorithms.

Algorithm	Parameter	Value
BAOA [40]	α	5
	Controlling parameter μ	0.01
	The maximum values of MOP	5
	The minimum values of MOP	0.2
BASO [44]	Depth weight, α	50
	Multiplier weight, β	0.2
BFPA [42]	Switch probablity, P	0.8
	Levy coefficient, β	1.5
BBA [41]	Maximum frequency, Fmax	2
	Minimum frequency, Fmin	0
	Two constants, α and γ	0.9
BCCSA [31]	Probability of awareness (AP)	0.2
	Flight length $fl_{\left(min\right)}$, $fl_{\left(max\right)}$	[1, 1.8]
BPSO [43]	Acceleration coefficients, C1 and C2	2
	Inertia weight	0.1
	Maximum inertia weight	0.9
	Minimum inertia weight	0.4
BDE [75]	β	32
	Constant factor F	[0, 2]
	Crossover constant CR	[0, 1]
	Global_minimum	1
	VTR	1.05

**Table 3:** The details of the datasets.

Dataset No.	Dataset Name	No. of Samples	No. of Features
D1	Chess	3196	36
D2	Wisconsin	699	9
D3	Breast	569	30
D4	Olive	572	8
D5	Lung Cancer	32	56
D6	Diabetes	144	7
D7	Heart	294	13
D8	Ionosphere	351	34
D9	Sonar	208	60
D10	Lymphography	148	18
D11	Hillvalley	606	100
D12	LSVT	126	310
D13	Zoo	101	16
D14	Hepatitis	80	19
D15	Diagnostic	569	30
D16	Coimbra	116	9
D17	BreastEW	568	30
D18	HeartEW	568	30
D19	SPECT	267	22
D20	Diabetes	768	8
D21	Cleveland	297	13
D22	ILPD	583	10
D23	Parkinsons	5875	19
D24	Dermatology	366	34
D25	PD Speech	756	753
D26	Heart Failure Clinical Records	299	12

**Table 4:** Ablation study of BCNRBO.

Variant	Fitness	Accuracy (%)
BNRBO (Original)	0.1739	82.61
BCNRBO (Full Model)	0.0435	96.00
w/o Dynamic Potential	0.1522	84.78
w/o Chaotic Mechanism	0.1826	81.74
w/o Transfer Function	0.1369	86.30

**Table 5:** Mean and standard deviation of fitness values for BNRBO and BCNRBO with ten chaotic maps.

Dataset	Algorithms											
		BNRBO	Map1	Map2	Map3	Map4	Map5	Map6	Map7	Map8	Map9	Map10
Chess	Mean	**0.0326**	0.0405	0.0408	0.0373	0.0357	0.0430	0.0463	0.0376	0.0418	0.0555	0.0342
	Std	0.0059	0.0069	0.0064	0.0085	0.0074	0.0088	0.0081	0.0051	0.0051	**0.0047**	0.0070
Lung Cancer	Mean	**0.0000**	**0.0000**	**0.0000**	**0.0000**	0.1250	0.0833	**0.0000**	**0.0000**	**0.0000**	**0.0000**	**0.0000**
	Std	0.0143	0.0275	0.0164	0.0175	0.0277	0.0124	0.0198	0.0182	0.0222	0.0150	**0.0123**
Diabetes	Mean	0.0714	0.0714	**0.0000**	0.0357	0.0357	0.0357	0.0357	**0.0000**	**0.0000**	0.0357	**0.0000**
	Std	**0.0000**	**0.0000**	**0.0000**	**0.0000**	**0.0000**	**0.0000**	**0.0000**	**0.0000**	**0.0000**	**0.0000**	**0.0000**
Sonar	Mean	0.0207	0.0805	0.0573	0.0427	0.0817	0.0354	0.0524	0.0524	0.0671	0.0537	**0.0146**
	Std	0.0134	0.0150	0.0142	0.0156	0.0157	0.0168	0.0160	0.0121	0.0145	0.0101	**0.0089**
Hillvalley	Mean	0.3826	**0.3103**	0.4029	0.4223	0.3413	0.3587	0.3260	0.3715	0.3694	0.3831	0.3223
	Std	0.0179	0.0325	0.0255	0.0799	**0.0000**	0.0398	**0.0000**	0.0325	0.0470	0.0440	0.0147
LSVT	Mean	0.3160	0.2260	0.2700	0.2760	0.2000	**0.1840**	0.2400	0.3460	0.2280	0.2620	0.3140
	Std	**0.0000**	**0.0000**	**0.0000**	**0.0000**	0.0740	0.0855	**0.0000**	**0.0000**	**0.0000**	**0.0000**	**0.0000**
PD Speech	Mean	0.2248	0.2500	0.2179	0.2159	0.1950	0.2056	0.1990	0.2136	0.2182	**0.1878**	0.2252
	Std	0.0391	0.0236	0.0436	0.0404	0.0479	0.0329	0.0070	0.0457	0.0586	0.0049	**0.0000**
Parkinsons	Mean	0.0068	0.0221	0.0184	0.0111	0.0106	0.0179	0.0077	**0.0034**	0.0162	0.0151	0.0060
	Std	**0.0000**	**0.0000**	0.0004	**0.0000**	0.0004	**0.0000**	0.0002	0.0002	**0.0000**	0.0006	**0.0000**
Dermatology	Mean	**0.0000**	**0.0000**	0.0021	0.0041	0.0027	**0.0000**	0.0137	**0.0000**	**0.0000**	**0.0000**	**0.0000**
	Std	**0.0000**	**0.0000**	0.0050	0.0064	0.0056	**0.0000**	**0.0000**	**0.0000**	**0.0000**	**0.0000**	**0.0000**
PD Speech	Mean	0.2248	0.2500	0.2179	0.2159	0.1950	0.2056	0.1990	0.2136	0.2182	**0.1878**	0.2252
	Std	0.0391	0.0236	0.0436	0.0404	0.0479	0.0329	0.0070	0.0457	0.0586	0.0049	**0.0000**

**Table 6:** Friedman test results for BNRBO and ten chaotic maps integrated with BCNRBO.

Dataset	BNRBO	BCNRBO									
		Map1	Map2	Map3	Map4	Map5	Map6	Map7	Map8	Map9	Map10
Chess	**3.1**	6.2	6.475	4.95	4.05	6.95	8.2	5.175	6.775	10.55	3.575
Lung Cancer	**5.375**	**5.375**	**5.375**	**5.375**	9.5	8.125	**5.375**	**5.375**	**5.375**	**5.375**	**5.375**
Diabetes	10.5	10.5	**2.5**	7	7	7	7	**2.5**	**2.5**	7	**2.5**
Sonar	2.5	8.95	6.975	5.15	9.175	4.325	6.2	6.35	7.95	6.475	**1.95**
Hillvalley	8.225	**1.5**	9.725	10.825	3.85	5.6	2.725	6.825	6.575	7.85	2.3
LSVT	9.35	4.225	6.75	6.375	2.375	**2.15**	4.925	10.325	4.275	6.125	9.125
Diagnostic	7.025	8.1	11	1.9	7.475	9.975	**1.1**	4.95	7.025	3.175	4.275
Parkinsons	3	11	9.825	5.75	5.25	9.175	4	**1**	7.95	7.05	2
Dermatology	**5.175**	**5.175**	6	6.825	6.275	**5.175**	10.675	**5.175**	**5.175**	**5.175**	**5.175**
PD Speech	7.225	9.175	6.6	5.9	4.225	5.1	5.25	6.15	5.75	**3.5**	7.125
Sum.	61.475	70.2	71.225	60.05	59.175	63.575	55.45	53.825	59.35	62.275	**43.4**
Avg.	6.148	7.020	7.123	6.005	5.918	6.358	5.545	5.383	5.935	6.228	**4.340**

**Table 7:** Wilcoxon rank-sum test results comparing BCNRBO_Map10 with nine chaotic-map variants of BCNRBO and the original BNRBO.

Dataset	BNRBO		Map10 vs. BCNRBO																	
			Map1		Map2		Map3		Map4		Map5		Map6		Map7		Map8		Map9	
	*p*-Value	R	*p*-Value	R	*p*-Value	R	*p*-Value	R	*p*-Value	R	*p*-Value	R	*p*-Value	R	*p*-Value	R	*p*-Value	R	*p*-Value	R
Chess	0.37118	0	0.03179	1	0.00373	1	0.13751	0	0.33755	0	0.0013	1	0.00029	1	0.04219	1	0.00297	1	0.00013	1
Lung Cancer	1	0	1	0	1	0	1	0	$6.1\times 10^{-5}$	1	0.00195	1	1	0	1	0	1	0	1	0
Diabetes	$7.7\times 10^{-6}$	1	$7.7\times 10^{-6}$	1	1	0	$7.7\times 10^{-6}$	1	$7.7\times 10^{-6}$	1	$7.7\times 10^{-6}$	1	$7.7\times 10^{-6}$	1	1	0	1	0	$7.7\times 10^{-6}$	1
Sonar	0.25781	0	$9.9\times 10^{-5}$	1	0.00018	1	0.00018	1	0.00012	1	0.00012	1	0.00026	1	0.00016	1	0.0001	1	0.00016	1
Hillvalley	$8.5\times 10^{-5}$	1	0.01344	−1	$8.3\times 10^{-5}$	1	$7.9\times 10^{-5}$	1	0.00194	1	0.00013	1	0.2913	0	$7.9\times 10^{-5}$	1	$8.1\times 10^{-5}$	1	$8.1\times 10^{-5}$	1
LSVT	1	0	$3.7\times 10^{-5}$	−1	0.00011	−1	0.00866	−1	$2.3\times 10^{-5}$	−1	$5.7\times 10^{-5}$	−1	$2.3\times 10^{-5}$	−1	0.00452	1	0.00018	−1	0.00012	1
Diagnostic	$7.7\times 10^{-6}$	1	$5.4\times 10^{-5}$	1	$7.7\times 10^{-6}$	1	$2.9\times 10^{-5}$	−1	$2.9\times 10^{-5}$	1	$1.2\times 10^{-5}$	1	$7.7\times 10^{-6}$	−1	0.0625	0	$7.7\times 10^{-6}$	1	$6.3\times 10^{-5}$	−1
Parkinsons	$7.7\times 10^{-6}$	1	$7.7\times 10^{-6}$	1	$4.7\times 10^{-5}$	1	$7.7\times 10^{-6}$	1	$5.4\times 10^{-5}$	1	$7.7\times 10^{-6}$	1	$1.2\times 10^{-5}$	1	$1.2\times 10^{-5}$	−1	$7.7\times 10^{-6}$	1	$6.1\times 10^{-5}$	1
Dermatology	1	0	1	0	0.25	0	0.03125	1	0.125	0	1	0	$7.7\times 10^{-6}$	1	1	0	1	0	1	0
PD Speech	0.83688	0	0.00093	1	1	0	0.33046	0	0.01471	−1	0.10488	0	$6.3\times 10^{-5}$	−1	0.95493	0	0.89558	0	$5.8\times 10^{-5}$	−1
Won		4		6		5		5		6		7		5		4		5		6
Loss		0		2		1		2		2		1		3		1		1		2
Equal		6		2		4		3		2		2		2		5		4		2

**Table 8:** Best obtained fitness values using KNN classifier.

Dataset	BCNRBO	BAOA	jBASO	BFPA	BBA	BCCSA	BDE	BABC	BPSO	BDA
D1	**0.025039**	0.051643	0.032864	0.046948	**0.025039**	0.059468	0.040689	0.039124	0.035994	**0.025039**
D2	**0.007194**	0.035971	0.014388	**0.007194**	0.014388	**0.007194**	0.035971	**0.007194**	**0.007194**	**0.007194**
D3	0.035398	0.026549	0.026549	0.053097	0.053097	0.035398	0.044248	0.035398	0.035398	**0.017699**
D4	0.087719	0.087719	**0.061404**	0.12281	0.087719	**0.061404**	0.10526	0.087719	0.087719	0.070175
D5	**0**	**0**	**0**	**0**	**0**	**0**	**0**	0.16667	**0**	**0**
D6	**0**	0.035714	0.035714	0.035714	0.035714	**0**	**0**	**0**	**0**	0.071429
D7	0.13793	**0.051724**	0.10345	0.068966	0.12069	0.13793	**0.051724**	0.15517	0.13793	0.12069
D8	0.028571	**0.014286**	0.071429	0.1	0.042857	0.12857	0.071429	0.057143	0.042857	**0.014286**
D9	**0**	0.02439	0.073171	0.073171	**0**	0.097561	0.097561	0.04878	0.02439	0.02439
D10	0.10345	0.10345	0.068966	0.13793	0.068966	0.068966	**0.034483**	0.10345	**0.034483**	0.068966
D11	**0.29752**	0.36364	0.35537	0.35537	0.38017	0.40496	0.3719	0.33058	0.3719	0.39669
D12	0.28	0.2	**0.16**	0.28	**0.16**	0.28	0.4	0.2	0.4	0.2
D13	0.05	**0**	0.05	**0**	**0**	**0**	**0**	**0**	**0**	0.05
D14	0.1875	0.125	0.125	0.125	**0**	0.0625	0.125	0.125	0.1875	0.125
D15	0.026549	0.044248	0.026549	0.044248	**0.00885**	0.053097	0.035398	0.017699	0.044248	0.035398
D16	0.086957	0.13043	0.17391	0.13043	**0**	0.043478	0.086957	0.086957	0.17391	0.13043
D17	0.026549	0.035398	**0.017699**	0.035398	0.026549	0.044248	0.026549	0.044248	0.035398	0.035398
D18	0.092593	**0.074074**	**0.074074**	0.092593	0.11111	0.14815	0.12963	0.092593	0.11111	0.11111
D19	0.16981	0.11321	0.15094	0.16981	0.13208	0.22642	0.16981	0.13208	0.18868	**0.09434**
D20	0.084746	0.10169	0.15254	0.15254	0.084746	0.15254	0.084746	**0.067797**	0.15254	**0.067797**
D21	0.22222	0.23529	**0.19608**	0.22876	0.21569	0.20915	0.22222	0.24183	0.20915	0.20915
D22	0.23276	0.21552	0.24138	0.25862	**0.19828**	0.25	0.24138	0.2069	0.25862	0.24138
D23	**0.005957**	0.021277	0.014468	0.024681	0.012766	0.01617	0.017021	0.020426	0.014468	0.019574
D24	**0**	**0**	**0**	**0**	**0**	**0**	**0**	**0**	**0**	**0**
D25	0.22517	0.17219	0.15894	0.25166	0.15894	0.22517	0.23179	0.24503	0.17881	**0.1457**
D26	0.11864	0.13559	0.13559	0.18644	0.20339	**0.10169**	0.13559	0.13559	0.11864	0.13559

**Table 9:** Average fitness values obtained using the KNN classifier.

Dataset	BCNRBO	BAOA	jBASO	BFPA	BBA	BCCSA	BDE	BABC	BPSO	BDA
D1	**0.034194**	0.064319	0.052739	0.057199	0.036385	0.063928	0.054773	0.046479	0.041628	0.034351
D2	0.007554	0.035971	0.016547	**0.007194**	0.020144	0.014748	0.035971	**0.007194**	**0.007194**	0.008633
D3	0.044248	0.026991	0.050442	0.059735	0.069912	0.047788	0.056637	0.043363	0.038496	**0.017699**
D4	0.087719	0.088158	0.069737	0.12281	0.089035	**0.069298**	0.10526	0.087719	0.087719	0.070175
D5	**0**	**0**	0.10833	0.15	**0**	**0**	**0**	0.16667	**0**	**0**
D6	**0**	0.035714	0.035714	0.035714	0.035714	**0**	**0**	**0**	**0**	0.071429
D7	0.14052	0.063793	0.11034	0.073276	0.12069	0.18879	**0.061207**	0.1569	0.14828	0.13017
D8	0.057857	**0.029286**	0.086429	0.105	0.077857	0.15714	0.081429	0.086429	0.054286	0.032857
D9	**0.014634**	0.064634	0.12195	0.10366	0.029268	0.13049	0.15366	0.080488	0.030488	0.045122
D10	0.11034	0.12586	0.096552	0.1569	0.10345	0.12759	0.058621	0.10345	**0.034483**	0.096552
D11	**0.32231**	0.38058	0.41157	0.36157	0.40496	0.43512	0.38223	0.35496	0.39215	0.42562
D12	0.314	0.264	0.202	0.308	0.274	0.288	0.456	**0.2**	0.446	0.206
D13	0.05	0.0275	0.05	0.0075	**0**	0.0275	**0**	**0**	**0**	0.055
D14	0.19375	0.125	0.125	0.15	0.14375	**0.1125**	0.125	0.20938	0.1875	0.125
D15	0.026549	0.04646	0.05354	0.044248	0.032301	0.071239	0.05	**0.017699**	0.050885	0.049115
**D16**	0.086957	0.15217	0.18478	0.13043	**0**	0.10217	0.086957	0.086957	0.17391	0.13043
D17	**0.026549**	0.04292	0.034956	0.043363	0.026991	0.050442	0.033186	0.052655	0.035398	0.035398
D18	0.1	0.098148	**0.074074**	0.10648	0.13704	0.22315	0.12963	0.10741	0.11481	0.12315
D19	0.20566	0.15283	0.18208	0.21038	0.17358	0.28113	0.19906	0.16792	0.2	**0.12736**
D20	**0.085593**	0.13898	0.17458	0.15424	0.10339	0.21186	0.11102	0.09322	0.15508	**0.085593**
D21	0.22222	0.23529	**0.19935**	0.22908	0.2232	0.2317	0.22222	0.24183	0.20915	0.20915
D22	0.23276	0.21595	0.24914	0.26078	0.21552	0.25345	0.25819	**0.20733**	0.25862	0.24138
D23	**0.005957**	0.022553	0.014468	0.025404	0.016979	0.021106	0.017872	0.020426	0.014468	0.019574
D24	**0**	**0**	**0**	**0**	**0**	0.012329	**0**	**0**	**0**	**0**
D25	0.22517	0.2	**0.19503**	0.25166	0.22053	0.2298	0.23742	0.25066	0.22947	0.20695
D26	**0.11864**	0.13559	0.13729	0.18644	0.20339	0.12034	0.14746	0.13559	**0.11864**	0.13559

**Table 10:** Friedman test results for BCNRBO vs. other algorithms with KNN classifier.

Dataset	BCNRBO	BAOA	jBASO	BFPA	BBA	BCCSA	BDE	BABC	BPSO	BDA
D1	**2.175**	9.05	6.55	7.275	2.6	9.3	6.925	5.125	3.7	2.3
D2	2.55	9.5	5.925	**2.425**	6.975	5.325	9.5	7.375	**2.425**	3
D3	5.5	2.1	6.6	8.375	9.825	6.15	7.575	3.625	4.25	**1**
D4	6.3	6.4	2.95	10	6.625	2.975	9	**1.275**	6.3	3.175
D5	**4.25**	**4.25**	7.3	8.725	**4.25**	**4.25**	**4.25**	9.225	**4.25**	**4.25**
D6	**3**	7.5	7.5	7.5	7.5	**3**	**3**	**3**	**3**	10
D7	7.05	1.875	4.375	2.475	5.05	9.875	**1.65**	8.725	7.875	6.05
D8	3.8	**1.575**	6.7	8.725	5.925	10	6.25	6.775	3.475	1.775
D9	**1.625**	5	8.225	7.25	2.575	8.525	9.575	5.85	2.675	3.7
D10	6.225	7.725	5.025	9.5	5.475	7.275	2.15	5.55	**1.15**	4.925
D11	**1**	4.725	7.75	2.85	7.075	9.525	4.925	2.35	6	8.8
D12	7.025	4.85	**2.3**	6.75	4.55	5.775	9.575	2.35	9.25	2.575
D13	8.325	6.1	8.325	4.1	**3.375**	6.1	**3.375**	**3.375**	**3.375**	8.55
D14	8.525	3.725	3.725	5.6	5.65	**3.15**	3.725	8.85	8.325	3.725
D15	2.35	5.825	6.525	5.175	3.625	9.65	7.1	**1.275**	7.15	6.325
D16	3.425	7.75	9.25	6.4	**1**	4.85	3.425	3.425	9.075	6.4
D17	**1.95**	7	5.15	7.2	2.075	8.95	3.925	9.5	4.625	4.625
D18	3.55	3.425	**1.075**	4.525	7.525	9.875	7.95	4.75	5.8	6.525
D19	7.2	2.675	5.05	7.575	4.375	9.925	6.525	3.65	6.65	**1.375**
D20	**2.125**	6.25	8.825	7.45	3.375	9.8	4.35	2.95	7.45	2.425
D21	5.5	8.4	**1.3**	7.25	5.65	6.825	5.5	9.575	2.5	2.5
D22	3.775	2.575	6.6	8.85	2.25	7.275	8.475	**1.475**	8.4	5.325
D23	**1**	8.575	3.05	9.925	4.9	7.475	4.675	6.725	3.05	5.625
D24	**5.175**	**5.175**	**5.175**	**5.175**	**5.175**	8.425	**5.175**	**5.175**	**5.175**	**5.175**
D25	4.45	**2.075**	2.3	9.425	4.75	5.75	7.25	9.275	6.2	3.525
D26	**2.05**	5.3	5.55	9	10	3.225	7.225	5.3	**2.05**	5.3
Summation	**109.900**	139.400	143.100	179.500	132.150	183.250	153.050	136.525	134.175	118.950
Average	**4.227**	5.362	5.504	6.904	5.083	7.048	5.887	5.251	5.161	4.575

**Table 11:** Wilcoxon signed-rank test results for BCNRBO vs. other algorithms with KNN classifier.

Dataset	BAOA		jBASO		BFPA		BBA		BCCSA		BDE		BABC		BPSO		BDA	
	*p*-Value	R	*p*-Value	R	*p*-Value	R	*p*-Value	R	*p*-Value	R	*p*-Value	R	*p*-Value	R	*p*-Value	R	*p*-Value	R
D1	$8.81\times 10^{-5}$	1	$8.73\times 10^{-5}$	1	$8.7\times 10^{-5}$	1	0.421649	0	$8.73\times 10^{-5}$	1	0.000154	1	0.000231	1	0.00088	1	0.982563	0
D2	$1.19\times 10^{-5}$	1	$4.26\times 10^{-5}$	1	1	0	$3.62\times 10^{-5}$	1	0.000105	1	$1.19\times 10^{-5}$	1	$1.19\times 10^{-5}$	1	1	0	0.375	0
D3	$9.43\times 10^{-5}$	−1	0.011963	1	0.000243	1	$9.43\times 10^{-5}$	1	0.391479	0	0.004354	1	0.000977	1	0.004395	1	$6.2\times 10^{-5}$	−1
D4	1	0	0.00029	−1	$7.74\times 10^{-6}$	1	0.25	0	$1.71\times 10^{-5}$	−1	$7.74\times 10^{-6}$	1	$7.74\times 10^{-6}$	−1	1	0	$7.74\times 10^{-6}$	−1
D5	1	0	0.000488	1	$2.21\times 10^{-5}$	1	1	0	1	0	1	0	$7.74\times 10^{-6}$	1	1	0	1	0
D6	$7.74\times 10^{-6}$	1	$7.74\times 10^{-6}$	1	$7.74\times 10^{-6}$	1	$7.74\times 10^{-6}$	1	1	0	1	0	1	0	1	0	$7.74\times 10^{-6}$	1
D7	$6.03\times 10^{-5}$	−1	$6.69\times 10^{-5}$	−1	$5.57\times 10^{-5}$	−1	$2.3\times 10^{-5}$	−1	0.000105	1	$6.39\times 10^{-5}$	−1	$3.38\times 10^{-5}$	1	0.011719	1	0.001953	1
D8	0.00025	−1	0.000392	1	$7.77\times 10^{-5}$	1	0.001496	1	$8.38\times 10^{-5}$	1	0.000407	1	0.000181	1	0.434082	0	0.00027	−1
D9	0.000118	1	$7.68\times 10^{-5}$	1	$6.19\times 10^{-5}$	1	0.003906	1	$6.26\times 10^{-5}$	1	$6.75\times 10^{-5}$	1	$7.19\times 10^{-5}$	1	0.000977	1	0.000327	1
D10	0.011719	1	0.217529	0	0.000142	1	0.125	0	0.010742	1	$5.62\times 10^{-5}$	−1	0.125	0	$2.93\times 10^{-5}$	−1	0.041016	1
D11	$7.75\times 10^{-5}$	1	$8.4\times 10^{-5}$	1	$7.12\times 10^{-5}$	1	$8.56\times 10^{-5}$	1	$8.4\times 10^{-5}$	1	$7.51\times 10^{-5}$	1	$7.88\times 10^{-5}$	1	$7.85\times 10^{-5}$	1	$8.29\times 10^{-5}$	1
D12	0.000194	−1	$7.39\times 10^{-5}$	−1	0.375	0	0.330354	0	0.000244	1	$4.26\times 10^{-5}$	1	$2.3\times 10^{-5}$	−1	$6.06\times 10^{-5}$	1	$2.97\times 10^{-5}$	−1
D13	0.003906	1	1	0	$3.74\times 10^{-5}$	−1	$7.74\times 10^{-6}$	−1	0.003906	1	$7.74\times 10^{-6}$	−1	$7.74\times 10^{-6}$	−1	$7.74\times 10^{-6}$	−1	0.5	0
D14	$1.71\times 10^{-5}$	−1	$1.71\times 10^{-5}$	−1	0.000488	1	0.000488	1	$4.15\times 10^{-5}$	−1	$1.71\times 10^{-5}$	−1	0.125	0	0.5	0	$1.71\times 10^{-5}$	−1
D15	$3.56\times 10^{-5}$	1	0.000179	1	$7.74\times 10^{-6}$	1	0.189651	0	$7.5\times 10^{-5}$	1	$4.26\times 10^{-5}$	1	$7.74\times 10^{-6}$	−1	$3.56\times 10^{-5}$	1	$5.6\times 10^{-5}$	1
D16	$5.4\times 10^{-5}$	1	$2.31\times 10^{-5}$	1	$7.74\times 10^{-6}$	1	$7.74\times 10^{-6}$	−1	0.75493	0	1	0	1	0	$7.74\times 10^{-6}$	1	$7.74\times 10^{-6}$	1
D17	$4.94\times 10^{-5}$	1	0.002827	1	$1.71\times 10^{-5}$	1	1	0	$4.15\times 10^{-5}$	1	$6.1\times 10^{-5}$	1	$1.19\times 10^{-5}$	1	$7.74\times 10^{-6}$	1	$7.74\times 10^{-6}$	1
D18	0.923828	0	$5.06\times 10^{-5}$	−1	0.09375	0	0.000183	1	$8.63\times 10^{-5}$	1	$5.06\times 10^{-5}$	1	0.080078	0	0.000244	1	$6.1\times 10^{-5}$	1
D19	0.000112	−1	0.00621	−1	0.484375	0	0.001468	−1	0.000123	1	0.334473	0	0.00011	−1	0.206328	0	$8.01\times 10^{-5}$	−1
D20	$6.72\times 10^{-5}$	1	$7.19\times 10^{-5}$	1	$2.31\times 10^{-5}$	1	0.001953	1	$7.32\times 10^{-5}$	1	0.000157	1	0.011719	1	$1.71\times 10^{-5}$	1	1	0
D21	$7.74\times 10^{-6}$	1	$3.56\times 10^{-5}$	−1	$1.19\times 10^{-5}$	1	0.826823	0	0.093832	0	1	0	$7.74\times 10^{-6}$	1	$7.74\times 10^{-6}$	−1	$7.74\times 10^{-6}$	−1
D22	$1.19\times 10^{-5}$	−1	$6.06\times 10^{-5}$	1	$3.56\times 10^{-5}$	1	0.000488	1	$4.32\times 10^{-5}$	1	$6.75\times 10^{-5}$	1	$1.19\times 10^{-5}$	−1	$7.74\times 10^{-6}$	1	$7.74\times 10^{-6}$	1
D23	$7.45\times 10^{-5}$	1	$7.74\times 10^{-6}$	1	$2.3\times 10^{-5}$	1	$5.31\times 10^{-5}$	1	$7.28\times 10^{-5}$	1	$2.31\times 10^{-5}$	1	$7.74\times 10^{-6}$	1	$7.74\times 10^{-6}$	1	$7.74\times 10^{-6}$	1
D24	1	0	1	0	1	0	1	0	0.000244	1	1	0	1	0	1	0	1	0
D25	$8.4\times 10^{-5}$	−1	0.000467	−1	$7.74\times 10^{-6}$	1	0.546875	0	0.000122	1	$6.39\times 10^{-5}$	1	$2.3\times 10^{-5}$	1	0.062374	0	0.013672	1
D26	$7.74\times 10^{-6}$	1	$1.71\times 10^{-5}$	1	$7.74\times 10^{-6}$	1	$7.74\times 10^{-6}$	1	0.672214	0	$4.15\times 10^{-5}$	1	$7.74\times 10^{-6}$	1	1	0	$7.74\times 10^{-6}$	1
Won		14		15		19		12		18		16		14		13		13
Loss		8		8		2		4		2		4		6		3		7
Equal		4		3		5		10		6		6		6		10		6

**Table 12:** Best obtained fitness values using DT classifier.

Dataset	BCNRBO	BAOA	jBASO	BFPA	BBA	BCCSA	BDE	BABC	BPSO	BDA
D1	**0.004695**	0.023474	0.00939	**0.004695**	**0.004695**	0.017214	0.007825	0.00626	0.00939	0.00939
D2	**0.007194**	**0.007194**	0.028777	0.028777	0.035971	0.021583	0.028777	0.014388	0.028777	0.014388
D3	**0**	0.044248	0.00885	0.00885	0.00885	0.017699	0.026549	0.00885	0.026549	0.026549
D4	0.078947	0.061404	0.070175	0.04386	0.078947	0.035088	0.061404	**0.026316**	0.052632	0.070175
D5	0.166667	**0**	**0**	0.166667	**0**	**0**	**0**	**0**	**0**	0.166667
D6	**0**	0.107143	0.035714	0.035714	0.035714	**0**	0.035714	0.035714	0.071429	0.035714
D7	0.103448	0.137931	**0.086207**	0.12069	0.103448	0.103448	0.103448	0.137931	0.137931	**0.086207**
D8	**0**	0.028571	0.014286	0.014286	0.028571	0.042857	0.028571	0.014286	0.042857	0.014286
D9	0.02439	0.04878	0.04878	0.097561	0.02439	0.146341	0.121951	0.097561	0.02439	**0**
D10	0.103448	0.068966	0.103448	0.034483	**0**	0.103448	0.103448	0.034483	0.068966	0.103448
D11	0.272727	0.31405	0.305785	0.289256	**0.214876**	0.371901	0.305785	0.272727	0.31405	0.247934
D12	**0.04**	**0.04**	**0.04**	0.08	**0.04**	0.12	**0.04**	**0.04**	**0.04**	0.08
D13	**0**	**0**	**0**	**0**	**0**	**0**	**0**	**0**	**0**	0.05
D14	**0**	0.125	0.125	0.0625	0.125	0.0625	**0**	0.125	0.125	0.0625
D15	0.00885	0.035398	0.017699	0.017699	**0**	**0**	0.026549	0.035398	0.00885	0.017699
D16	0.173913	0.086957	0.086957	0.086957	0.173913	0.217391	0.130435	0.173913	**0**	0.173913
D17	0.00885	0.00885	0.026549	0.017699	0.017699	0.044248	0.026549	0.00885	**0**	**0**
D18	0.12963	0.111111	0.111111	0.148148	0.092593	0.148148	0.092593	0.12963	**0.055556**	0.074074
D19	0.207547	0.132075	0.150943	0.169811	0.132075	0.226415	0.150943	**0.113208**	0.150943	0.150943
D20	**0.084746**	0.101695	0.101695	0.101695	0.135593	0.135593	0.118644	0.118644	0.135593	**0.084746**
D21	0.281046	0.267974	0.228758	0.235294	0.235294	0.228758	0.24183	**0.215686**	0.24183	0.24183
D22	0.224138	0.224138	0.241379	0.25	0.258621	0.224138	**0.206897**	0.224138	0.241379	0.241379
D23	0.01617	0.04	**0.006809**	0.040851	0.012766	0.059574	0.04766	0.036596	0.026383	0.015319
D24	**0**	0.013699	0.013699	0.013699	**0**	0.013699	**0**	0.013699	0.027397	**0**
D25	0.05298	0.099338	0.086093	0.072848	**0.046358**	0.139073	0.099338	0.13245	0.066225	0.059603
D26	0.118644	0.152542	0.101695	0.084746	0.118644	0.118644	0.118644	**0.050847**	0.067797	0.118644

**Table 13:** Average fitness values obtained using the DT classifier.

Dataset	BCNRBO	BAOA	jBASO	BFPA	BBA	BCCSA	BDE	BABC	BPSO	BDA
D1	0.00626	0.035681	0.011581	0.00759	**0.004851**	0.017214	0.012207	0.007981	0.012911	0.012676
D2	**0.007194**	0.007554	0.033813	0.028777	0.042806	0.034532	0.029137	0.014388	0.029137	0.014388
D3	**0.003097**	0.05177	0.019027	0.021681	0.015929	0.030973	0.034956	0.015487	0.031416	0.038938
D4	0.078947	0.065789	0.077632	0.04386	0.079386	0.059211	0.061404	**0.026316**	0.053509	0.070175
D5	0.2	0.141667	0.141667	0.166667	0.15	0.141667	**0**	0.141667	**0**	0.166667
D6	**0**	0.107143	0.05	0.035714	0.035714	0.001786	0.035714	0.035714	0.071429	0.035714
D7	0.114655	0.155172	**0.086207**	0.152586	0.114655	0.113793	0.10431	0.137931	0.137931	0.091379
D8	0.019286	0.046429	0.027143	0.027857	0.032857	0.054286	0.029286	0.027857	0.067143	**0.015714**
D9	0.052439	0.069512	0.084146	0.126829	0.057317	0.192683	0.143902	0.117073	0.063415	**0.045122**
D10	0.113793	0.106897	0.103448	0.037931	**0.027586**	0.155172	0.103448	0.034483	0.124138	0.105172
D11	0.301653	0.34876	0.327686	0.320661	**0.263636**	0.392149	0.328512	0.301653	0.339256	0.281818
D12	0.05	0.07	0.108	0.082	**0.04**	0.18	0.042	**0.04**	0.11	0.106
D13	**0**	**0**	0.01	0.0025	0.02	0.0375	0.005	**0**	**0**	0.05
D14	**0.034375**	0.134375	0.175	0.103125	0.178125	0.175	0.109375	0.125	0.18125	0.09375
D15	**0.010177**	0.038938	0.031416	0.025664	0.016372	0.019912	0.029204	0.035841	0.023451	0.022566
D16	0.173913	0.086957	0.086957	0.086957	0.184783	0.271739	0.130435	0.180435	**0**	0.173913
D17	0.015044	0.023009	0.033186	0.019469	0.025664	0.052655	0.031858	0.017257	0.008407	**0**
D18	0.14537	0.131481	0.116667	0.168519	0.092593	0.173148	0.092593	0.138889	**0.055556**	0.082407
D19	0.220755	0.15566	0.184906	0.2	**0.136792**	0.256604	0.181132	0.150943	0.187736	0.174528
D20	0.101695	0.116949	0.118644	0.114407	0.138136	0.170339	0.118644	0.121186	0.138136	**0.098305**
D21	0.281046	0.269935	0.231699	0.235294	0.236601	0.251634	0.24183	**0.215686**	0.242157	0.242157
D22	0.225	0.228879	0.246983	0.25	0.269397	0.278017	**0.211638**	0.227155	0.243534	0.242241
D23	0.018851	0.06383	**0.018468**	0.050298	0.058468	0.086255	0.059404	0.058681	0.040596	0.025064
D24	**0**	0.013699	0.017808	0.015753	0.000685	0.028767	0.013014	0.013699	0.039726	0.003425
D25	**0.067219**	0.110596	0.111589	0.09404	0.068543	0.168874	0.119205	0.142053	0.074503	0.080795
D26	0.118644	0.152542	0.104237	0.090678	0.120339	0.160169	0.118644	**0.055085**	0.067797	0.118644

**Table 14:** Friedman test results for BCNRBO vs. other algorithms with DT classifier.

Dataset	BCNRBO	BAOA	jBASO	BFPA	BBA	BCCSA	BDE	BABC	BPSO	BDA
D1	2.45	10	5.95	3.275	**1.375**	8.925	6.325	3.4	6.65	6.65
D2	**1.475**	1.575	7.575	6.425	9.7	8.175	6.55	3.475	6.575	3.475
D3	**1.15**	9.875	3.85	4.575	3.125	6.725	7.65	3.025	6.7	8.325
D4	8.975	5.65	8.45	2.175	9.05	4.85	4.725	**1**	3.425	6.7
D5	7.5	6	6.025	6.75	6.25	6.025	**1.85**	6	**1.85**	6.75
D6	**1.475**	10	6.7	5.275	5.275	1.65	5.275	5.275	8.8	5.275
D7	4.8	9.475	**1.35**	9.225	4.8	4.7	3.625	7.475	7.475	2.075
D8	2.925	7.8	4.3	4.6	5.525	8.775	4.925	4.625	9.625	**1.9**
D9	2.775	4.15	5.35	7.8	3.175	9.875	8.625	7.15	3.675	**2.425**
D10	6.95	6.375	6	2.175	**1.775**	9.525	6	2.05	8.025	6.125
D11	3.6	8.4	6.4	5.475	**1.5**	10	6.45	3.375	7.775	2.025
D12	3.375	5.15	7.6	6.125	**2.575**	9.875	2.75	**2.575**	7.375	7.6
D13	**4.25**	**4.25**	5.25	4.5	6.25	8	4.75	**4.25**	**4.25**	9.25
D14	**1.35**	5.5	7.7	3.875	8.025	7.65	4.25	4.925	8.325	3.4
D15	**1.4**	9.175	7.3	5.5	2.875	3.925	6.55	8.675	4.95	4.65
D16	7.3	3	3	3	7.825	9.9	5	7.675	**1**	7.3
D17	3.875	6	8.075	5.1	6.325	9.975	7.975	4.425	2.225	**1.025**
D18	7.575	6.35	5.425	9.275	3.275	9.425	3.275	6.95	**1**	2.45
D19	8.825	3.05	5.725	7.125	**1.6**	9.95	5.375	2.55	5.95	4.85
D20	2.225	4.8	5.1	4.275	8.25	9.85	5.025	5.4	8.25	**1.825**
D21	9.95	9.05	2.55	3.375	3.6	7.025	6.1	**1**	6.175	6.175
D22	2.575	3.35	6.625	7.625	9.2	9.35	**1.375**	2.95	6.125	5.825
D23	2.45	7.25	**2.15**	5.4	7.225	9.825	7.125	6.875	4.075	2.625
D24	**1.875**	5.725	6.625	6.175	2.05	8.6	5.5	5.725	9.775	2.95
D25	**1.9**	6.6	6.7	4.875	1.925	9.95	7.575	9.05	2.825	3.6
D26	6.35	9.375	4.1	3.525	6.575	9.375	6.35	**1.125**	1.875	6.35
Summation	**109.350**	167.925	145.875	137.500	129.125	211.900	140.975	121.000	144.750	121.600
Average	**4.206**	6.459	5.611	5.288	4.966	8.150	5.422	4.654	5.567	4.677

**Table 15:** Wilcoxon signed-rank test results for BCNRBO vs. other algorithms with DT classifier.

Dataset	BAOA		jBASO		BFPA		BBA		BCCSA		BDE		BABC		BPSO		BDA	
	*p*-Value	R	*p*-Value	R	*p*-Value	R	*p*-Value	R	*p*-Value	R	*p*-Value	R	*p*-Value	R	*p*-Value	R	*p*-Value	R
D1	$8.76\times 10^{-5}$	1	0.000184	1	0.080139	0	0.022461	1	$6.59\times 10^{-5}$	1	0.000188	1	0.0183	1	0.000128	1	0.000107	1
D2	1	0	$4.67\times 10^{-5}$	1	$7.74\times 10^{-6}$	1	$6.86\times 10^{-5}$	1	$5.02\times 10^{-5}$	1	$1.19\times 10^{-5}$	1	$7.74\times 10^{-6}$	1	$1.19\times 10^{-5}$	1	$7.74\times 10^{-6}$	1
D3	$6.91\times 10^{-5}$	1	$4.26\times 10^{-5}$	1	$9.84\times 10^{-5}$	1	0.000117	1	$7.03\times 10^{-5}$	1	$4.83\times 10^{-5}$	1	0.000171	1	$7.5\times 10^{-5}$	1	$7.5\times 10^{-5}$	1
D4	$7.93\times 10^{-5}$	−1	0.581055	0	$7.74\times 10^{-6}$	−1	1	0	0.000176	−1	$7.74\times 10^{-6}$	−1	$7.74\times 10^{-6}$	−1	$1.19\times 10^{-5}$	−1	$7.74\times 10^{-6}$	−1
D5	0.015625	1	0.03125	1	0.125	0	0.03125	1	0.03125	1	$2.93\times 10^{-5}$	−1	0.015625	1	$2.93\times 10^{-5}$	−1	0.125	0
D6	$7.74\times 10^{-6}$	1	$5.06\times 10^{-5}$	1	$7.74\times 10^{-6}$	1	$7.74\times 10^{-6}$	1	1	0	$7.74\times 10^{-6}$	1	$7.74\times 10^{-6}$	1	$7.74\times 10^{-6}$	1	$7.74\times 10^{-6}$	1
D7	$6.07\times 10^{-5}$	1	$4.67\times 10^{-5}$	−1	$5.47\times 10^{-5}$	1	1	0	0.818546	0	0.000488	1	$4.67\times 10^{-5}$	1	$4.67\times 10^{-5}$	1	0.000185	−1
D8	0.000177	1	0.159058	0	0.016357	1	0.000122	1	$8.12\times 10^{-5}$	1	0.000488	1	0.001953	1	$7.71\times 10^{-5}$	1	0.039063	1
D9	0.002197	1	$6.1\times 10^{-5}$	1	$7.19\times 10^{-5}$	1	0.283203	0	$8.29\times 10^{-5}$	1	$7.14\times 10^{-5}$	1	$8.12\times 10^{-5}$	1	0.072205	0	0.256348	0
D10	0.289063	0	0.03125	1	$4.26\times 10^{-5}$	−1	$7.7\times 10^{-5}$	−1	0.000768	1	0.03125	1	$4.15\times 10^{-5}$	−1	0.122308	0	0.0625	0
D11	0.000125	1	$9.99\times 10^{-5}$	1	$6.1\times 10^{-5}$	1	0.000279	−1	$8.07\times 10^{-5}$	1	0.000181	1	0.874756	0	0.000123	1	0.001309	−1
D12	0.116333	0	0.000216	1	0.009311	1	0.25	0	0.0001	1	0.375	0	0.25	0	0.000439	1	0.000299	1
D13	1	0	0.125	0	1	0	0.007813	1	$6.1\times 10^{-5}$	1	0.5	0	1	0	1	0	$7.74\times 10^{-6}$	1
D14	$5.79\times 10^{-5}$	1	$6.56\times 10^{-5}$	1	$6.1\times 10^{-5}$	1	$6.53\times 10^{-5}$	1	$5.06\times 10^{-5}$	1	0.000329	1	$5.31\times 10^{-5}$	1	$4.67\times 10^{-5}$	1	$6.1\times 10^{-5}$	1
D15	$6.2\times 10^{-5}$	1	$6.03\times 10^{-5}$	1	$6.53\times 10^{-5}$	1	0.000465	1	0.001099	1	$4.94\times 10^{-5}$	1	$2.96\times 10^{-5}$	1	0.000131	1	$5.06\times 10^{-5}$	1
D16	$7.74\times 10^{-6}$	−1	$7.74\times 10^{-6}$	−1	$7.74\times 10^{-6}$	−1	0.0625	0	$5.62\times 10^{-5}$	1	$7.74\times 10^{-6}$	−1	0.25	0	$7.74\times 10^{-6}$	−1	1	0
D17	0.000244	1	0.000115	1	0.027344	1	0.000732	1	$7.19\times 10^{-5}$	1	$5.57\times 10^{-5}$	1	0.226563	0	0.000488	1	$6.12\times 10^{-5}$	−1
D18	0.010075	−1	$5.62\times 10^{-5}$	−1	0.000192	1	$5.48\times 10^{-5}$	−1	0.000438	1	$5.48\times 10^{-5}$	−1	0.143066	0	$5.48\times 10^{-5}$	−1	$6.6\times 10^{-5}$	−1
D19	$7.48\times 10^{-5}$	−1	0.000167	−1	0.000545	−1	$6.4\times 10^{-5}$	−1	0.000108	1	$7.02\times 10^{-5}$	−1	$7.18\times 10^{-5}$	−1	0.000101	−1	$7.65\times 10^{-5}$	−1
D20	0.000488	1	0.000217	1	0.002686	1	$4.39\times 10^{-5}$	1	$7.94\times 10^{-5}$	1	$4.78\times 10^{-5}$	1	$8\times 10^{-5}$	1	$4.39\times 10^{-5}$	1	0.1875	0
D21	$4.78\times 10^{-5}$	−1	$5.31\times 10^{-5}$	−1	$7.74\times 10^{-6}$	−1	$1.19\times 10^{-5}$	−1	$7.67\times 10^{-5}$	−1	$7.74\times 10^{-6}$	−1	$7.74\times 10^{-6}$	−1	$1.19\times 10^{-5}$	−1	$1.19\times 10^{-5}$	−1
D22	0.044922	1	$6.2\times 10^{-5}$	1	$1.71\times 10^{-5}$	1	$7.06\times 10^{-5}$	1	0.000122	1	0.000218	−1	0.125	0	$3.62\times 10^{-5}$	1	$2.97\times 10^{-5}$	1
D23	$8.83\times 10^{-5}$	1	0.24283	0	$8.72\times 10^{-5}$	1	0.000383	1	$8.82\times 10^{-5}$	1	$8.77\times 10^{-5}$	1	$8.79\times 10^{-5}$	1	0.000103	1	0.05419	0
D24	$7.74\times 10^{-6}$	1	$4.15\times 10^{-5}$	1	$2.3\times 10^{-5}$	1	1	0	$5.57\times 10^{-5}$	1	$1.31\times 10^{-5}$	1	$7.74\times 10^{-6}$	1	$1.71\times 10^{-5}$	1	0.0625	0
D25	$8.31\times 10^{-5}$	1	0.000129	1	$8.49\times 10^{-5}$	1	0.958643	0	$8.75\times 10^{-5}$	1	$8.5\times 10^{-5}$	1	$7.95\times 10^{-5}$	1	0.016987	1	0.001889	1
D26	$7.74\times 10^{-6}$	1	$3.74\times 10^{-5}$	−1	$5.7\times 10^{-5}$	−1	0.5	0	0.000174	1	1	0	$3.56\times 10^{-5}$	−1	$7.74\times 10^{-6}$	−1	1	0
Won		17		16		17		13		22		16		14		16		11
Loss		5		6		6		5		2		7		5		7		7
Equal		4		4		3		8		2		3		7		3		8

**Table 16:** Best obtained fitness values using NB classifier.

Dataset	BCNRBO	BAOA	jBASO	BFPA	BBA	BCCSA	BDE	BABC	BPSO	BDA
D1	0.156495	0.167449	0.156495	0.170579	0.14241	0.203443	0.161189	0.14241	0.156495	**0.137715**
D2	**0.007194**	**0.007194**	**0.007194**	0.021583	0.014388	0.028777	0.035971	0.021583	0.014388	0.043165
D3	0.00885	0.00885	**0**	0.017699	0.017699	0.017699	0.035398	0.00885	0.00885	0.00885
D4	0.035088	**0.026316**	0.035088	0.070175	0.052632	0.04386	0.035088	0.061404	0.052632	0.035088
D5	**0**	**0**	**0**	**0**	**0**	**0**	**0**	0.166667	**0**	0.166667
D6	**0**	**0**	**0**	**0**	**0**	**0**	0.035714	**0**	**0**	**0**
D7	**0.068966**	0.155172	0.137931	0.103448	0.086207	0.103448	0.155172	0.103448	0.12069	0.103448
D8	0.014286	0.028571	0.014286	0.042857	**0**	**0**	0.014286	0.014286	0.014286	**0**
D9	0.073171	0.146341	0.02439	0.073171	**0**	0.097561	0.146341	0.121951	**0**	0.097561
D10	0.068966	0.103448	0.068966	0.034483	0.034483	0.068966	**0**	0.068966	0.103448	0.103448
D11	**0.371901**	0.487603	0.454545	0.404959	0.429752	0.396694	0.446281	0.487603	0.446281	0.454545
D12	0.12	0.08	**0.04**	0.2	0.08	0.2	0.08	0.16	0.08	**0.04**
D13	**0.05**	0.1	**0.05**	**0.05**	**0.05**	0.1	**0.05**	**0.05**	**0.05**	**0.05**
D14	**0**	0.125	0.125	0.125	**0**	0.1875	0.125	**0**	0.1875	0.0625
D15	**0**	0.026549	0.00885	0.026549	0.00885	**0**	0.00885	0.017699	0.026549	0.017699
D16	0.173913	0.173913	0.173913	**0.043478**	0.130435	0.086957	0.217391	0.086957	0.086957	0.173913
D17	0.00885	0.00885	0.026549	0.00885	0.017699	0.035398	**0**	0.017699	**0**	**0**
D18	**0.055556**	0.074074	0.074074	0.12963	0.111111	0.12963	0.111111	0.111111	0.092593	**0.055556**
D19	**0.113208**	0.207547	**0.113208**	0.132075	0.245283	0.226415	0.245283	0.169811	**0.113208**	0.207547
D20	0.067797	0.169492	0.101695	**0.050847**	0.135593	0.118644	0.101695	0.152542	0.084746	0.135593
D21	0.196078	0.24183	0.215686	0.196078	0.176471	0.215686	**0.156863**	0.202614	0.202614	0.176471
D22	**0.181034**	0.232759	0.241379	0.258621	0.206897	0.25	0.224138	0.215517	0.25	0.206897
D23	**0.215319**	0.237447	0.231489	0.241702	0.234894	0.225532	0.233191	0.244255	0.245106	0.228936
D24	0.013699	0.027397	**0**	**0**	**0**	0.027397	**0**	0.013699	**0**	**0**
D25	0.211921	0.198675	0.125828	0.192053	**0.119205**	0.205298	0.18543	0.192053	0.165563	0.198675
D26	**0.084746**	0.169492	0.169492	0.135593	0.152542	0.135593	0.135593	0.118644	**0.084746**	0.101695

**Table 17:** Average fitness values obtained using the NB classifier.

Dataset	BCNRBO	BAOA	jBASO	BFPA	BBA	BCCSA	BDE	BABC	BPSO	BDA
D1	0.163615	0.191628	0.164241	0.178091	0.150782	0.237715	0.170892	**0.149139**	0.166432	0.151487
D2	**0.007194**	0.010432	0.011151	0.021583	0.014388	0.034532	0.035971	0.021583	0.014388	0.043525
D3	0.015044	0.026549	0.009292	0.024336	0.019912	0.026991	0.042478	0.023451	**0.00885**	0.018584
D4	0.035088	**0.02807**	0.041667	0.070175	0.052632	0.057456	0.035088	0.061404	0.05307	0.035526
D5	0.075	**0**	0.133333	**0**	**0**	0.008333	**0**	0.166667	**0**	0.166667
D6	**0**	**0**	0.005357	**0**	**0**	0.001786	0.035714	**0**	**0**	**0**
D7	**0.069828**	0.155172	0.164655	0.133621	0.087069	0.110345	0.168103	0.113793	0.12069	0.111207
D8	0.039286	0.044286	0.022857	0.055714	0.017857	**0.010714**	0.014286	0.03	0.022857	0.013571
D9	0.135366	0.17561	0.078049	0.103659	**0.018293**	0.136585	0.164634	0.162195	0.042683	0.152439
D10	0.086207	0.124138	0.091379	0.043103	0.056897	0.155172	**0**	0.101724	0.131034	0.115517
D11	**0.385124**	0.499587	0.468595	0.409504	0.438843	0.408678	0.446281	0.495455	0.448347	0.467355
D12	0.138	0.122	0.128	0.232	0.12	0.242	0.118	0.198	0.12	**0.088**
D13	0.0525	0.105	0.065	**0.05**	0.0525	0.145	**0.05**	**0.05**	**0.05**	0.0525
D14	0.059375	0.15	0.171875	0.125	**0**	0.2625	0.125	0.059375	0.1875	0.13125
D15	**0**	0.034956	0.018584	0.035398	0.014602	0.012389	0.00885	0.019027	0.034513	0.022124
D16	0.173913	0.182609	0.176087	**0.045652**	0.13913	0.119565	0.217391	0.086957	0.086957	0.176087
D17	0.020796	0.009292	0.030088	0.032743	0.022124	0.050885	0.00708	0.017699	**0.000442**	0.004867
D18	**0.062037**	0.099074	0.077778	0.12963	0.113889	0.155556	0.112963	0.117593	0.093519	0.068519
D19	**0.113208**	0.215094	0.129245	0.170755	0.265094	0.261321	0.258491	0.184906	0.143396	0.227358
D20	0.081356	0.177119	0.107627	**0.066102**	0.15339	0.148305	0.101695	0.157627	0.087288	0.135593
D21	0.196732	0.24183	0.217647	0.196078	0.177451	0.227778	**0.156863**	0.202614	0.202614	0.176471
D22	**0.187931**	0.242241	0.246983	0.270259	0.216379	0.272845	0.233621	0.218966	0.25	0.209052
D23	**0.218553**	0.244383	0.236043	0.254936	0.238894	0.24834	0.243191	0.255574	0.250511	0.232383
D24	0.023973	0.05274	0.003425	0.002055	0.008904	0.076027	0.012329	0.026027	0.00137	**0.000685**
D25	0.231457	0.211258	0.138411	0.204305	**0.135099**	0.218874	0.192715	0.19702	0.176821	0.212583
D26	0.097458	0.172034	0.170339	0.14661	0.152542	0.157627	0.135593	0.131356	**0.084746**	0.101695

**Table 18:** Friedman test results for BCNRBO vs. other algorithms with NB classifier.

Dataset	BCNRBO	BAOA	jBASO	BFPA	BBA	BCCSA	BDE	BABC	BPSO	BDA
D1	4.85	8.8	5.1	7.875	2.3	9.9	6.425	**1.725**	5.525	2.5
D2	**1.5**	2.625	2.875	6.5	4	8.45	8.6	6.5	4	9.95
D3	3.6	6.9	2.4	6.725	5	7.45	9.925	6.45	**1.775**	4.775
D4	3.175	**1.375**	4.55	9.95	6.575	7.65	3.175	8.6	6.675	3.275
D5	6.1	**3.85**	7.85	**3.85**	**3.85**	4.1	**3.85**	8.85	**3.85**	8.85
D6	**4.9**	**4.9**	5.65	**4.9**	**4.9**	5.15	9.9	**4.9**	**4.9**	**4.9**
D7	**1.025**	8.225	9.075	6.525	2.025	4.325	9.4	4.575	5.55	4.275
D8	7.675	8.6	5	9.825	3.925	**2.375**	3.025	6.475	5.05	3.05
D9	6.05	8.875	3.15	4.25	**1.175**	6.075	8.2	8.025	1.95	7.25
D10	4.9	7.925	5.3	2.475	3.075	9	**1**	6.025	8.375	6.925
D11	**1**	9.75	7.55	2.525	4.2	2.475	5.25	9.25	5.575	7.425
D12	5.325	4.3	4.5	9.3	4.075	9.45	3.975	8.05	4.1	**1.925**
D13	4.575	8.875	5.2	**4.35**	4.575	9.8	**4.35**	**4.35**	**4.35**	4.575
D14	2.575	6.575	7.55	5.375	**1.05**	9.875	5.375	2.575	8.325	5.725
D15	**1.05**	8.85	5.325	8.925	4.175	3.65	2.6	5.425	8.8	6.2
D16	7.25	7.775	7.375	**1.075**	5.075	4.025	9.85	2.6	2.6	7.375
D17	6.225	3.475	8.05	8.275	6.675	9.85	2.95	5.675	**1.4**	2.425
D18	**1.5**	5	2.725	8.75	6.775	9.875	6.7	7.3	4.3	2.075
D19	**1.125**	6.325	2.1	4.1	9.05	8.9	8.825	4.825	2.85	6.9
D20	2.275	9.75	4.675	**1.075**	7.975	7.575	4.325	8.325	2.8	6.225
D21	4.625	9.925	8.25	4.425	2.575	8.825	**1**	6.45	6.45	2.475
D22	**1.325**	6.1	6.95	9.325	3.15	9.55	5.15	3.675	7.55	2.225
D23	**1**	5.675	3.625	8.95	4.025	6.875	5.45	9	7.775	2.625
D24	6.95	9.2	3.325	2.925	4.625	9.625	5.4	7.55	2.775	**2.625**
D25	9.85	7.275	1.6	6.25	**1.4**	8.425	4.325	5.125	3.025	7.725
D26	2.25	9.3	9.175	6.3	7.125	7.55	4.925	4.625	**1.125**	2.625
Summation	**102.675**	180.225	138.925	154.8	113.35	190.8	143.95	156.925	121.45	126.9
Average	**3.949038**	6.931731	5.343269	5.953846	4.359615	7.338462	5.536538	6.035577	4.671154	4.880769

**Table 19:** Wilcoxon signed-rank test results for BCNRBO vs. other algorithms with NB classifier.

Dataset	BAOA		jBASO		BFPA		BBA		BCCSA		BDE		BABC		BPSO		BDA	
	*p*-Value	R	*p*-Value	R	*p*-Value	R	*p*-Value	R	*p*-Value	R	*p*-Value	R	*p*-Value	R	*p*-Value	R	*p*-Value	R
D1	$8.79\times 10^{-5}$	1	0.428794	0	$8.66\times 10^{-5}$	1	0.000391	−1	$8.81\times 10^{-5}$	1	0.001323	1	0.000128	−1	0.082975	0	0.000129	−1
D2	0.003906	1	0.000977	1	$7.74\times 10^{-6}$	1	$7.74\times 10^{-6}$	1	$5.47\times 10^{-5}$	1	$7.74\times 10^{-6}$	1	$7.74\times 10^{-6}$	1	$7.74\times 10^{-6}$	1	$1.19\times 10^{-5}$	1
D3	0.000769	1	0.01416	1	$6.32\times 10^{-5}$	1	0.007813	1	0.000113	1	$6.18\times 10^{-5}$	1	0.000829	1	0.000122	1	0.055664	0
D4	$6.33\times 10^{-5}$	−1	0.007813	1	$7.74\times 10^{-6}$	1	$7.74\times 10^{-6}$	1	$6.26\times 10^{-5}$	1	1	0	$7.74\times 10^{-6}$	1	$1.19\times 10^{-5}$	1	1	0
D5	0.003906	1	0.039063	1	0.003906	1	0.003906	1	0.021484	1	0.003906	1	0.000977	1	0.003906	1	0.000977	1
D6	1	0	0.25	0	1	0	1	0	1	0	$7.74\times 10^{-6}$	1	1	0	1	0	1	0
D7	$1.19\times 10^{-5}$	1	$5.69\times 10^{-5}$	1	$7.4\times 10^{-5}$	1	$2.01\times 10^{-5}$	1	$5.62\times 10^{-5}$	1	$4.26\times 10^{-5}$	1	$6.18\times 10^{-5}$	1	$1.19\times 10^{-5}$	1	$4.39\times 10^{-5}$	1
D8	0.15625	0	0.001404	1	0.000156	1	0.000225	−1	$9.95\times 10^{-5}$	−1	$9.7\times 10^{-5}$	−1	0.046647	−1	0.001022	−1	0.000189	−1
D9	0.000363	1	0.000179	−1	0.000488	1	$7.95\times 10^{-5}$	−1	1	0	0.001651	1	0.000854	1	$7.12\times 10^{-5}$	−1	0.131165	0
D10	0.000242	1	0.3125	0	0.00022	−1	0.00077	−1	0.000145	1	$5.4\times 10^{-5}$	−1	0.011719	1	0.000144	1	0.000977	1
D11	$7.78\times 10^{-5}$	1	$8.27\times 10^{-5}$	1	$7.24\times 10^{-5}$	1	$6.74\times 10^{-5}$	1	$7.59\times 10^{-5}$	1	$5.6\times 10^{-5}$	1	$6.19\times 10^{-5}$	1	$6.36\times 10^{-5}$	1	$6.74\times 10^{-5}$	1
D12	0.072266	0	0.154408	0	$6.36\times 10^{-5}$	1	0.017578	1	$6.59\times 10^{-5}$	1	0.001953	1	$7.93\times 10^{-5}$	1	0.019531	1	0.000194	−1
D13	$2.86\times 10^{-5}$	1	0.25	0	1	0	1	0	$2.31\times 10^{-5}$	1	1	0	1	0	1	0	1	0
D14	$5.3\times 10^{-5}$	1	$4.26\times 10^{-5}$	1	$1.19\times 10^{-5}$	1	$1.31\times 10^{-5}$	−1	$5.6\times 10^{-5}$	1	$1.19\times 10^{-5}$	1	1	0	$1.19\times 10^{-5}$	1	0.00029	1
D15	$3.65\times 10^{-5}$	1	$5.05\times 10^{-5}$	1	$5.61\times 10^{-5}$	1	$6.2\times 10^{-5}$	1	0.000124	1	$7.74\times 10^{-6}$	1	$2.3\times 10^{-5}$	1	$5.57\times 10^{-5}$	1	$5.4\times 10^{-5}$	1
D16	0.125	0	1	0	$1.19\times 10^{-5}$	−1	$6.33\times 10^{-5}$	−1	$3.56\times 10^{-5}$	−1	$7.74\times 10^{-6}$	1	$7.74\times 10^{-6}$	−1	$7.74\times 10^{-6}$	−1	1	0
D17	0.0002	−1	0.000122	1	0.002116	1	0.613281	0	0.000113	1	0.000151	−1	0.113281	0	$5.68\times 10^{-5}$	−1	0.000101	−1
D18	0.000114	1	$6.1\times 10^{-5}$	1	$4.67\times 10^{-5}$	1	$5.47\times 10^{-5}$	1	$7.11\times 10^{-5}$	1	$5.87\times 10^{-5}$	1	$5.61\times 10^{-5}$	1	$5.3\times 10^{-5}$	1	0.091797	0
D19	$5.06\times 10^{-5}$	1	$3.74\times 10^{-5}$	1	$2.96\times 10^{-5}$	1	$4.37\times 10^{-5}$	1	$7.25\times 10^{-5}$	1	$4.15\times 10^{-5}$	1	$2.93\times 10^{-5}$	1	0.00011	1	$6.12\times 10^{-5}$	1
D20	$6.36\times 10^{-5}$	1	$5.66\times 10^{-5}$	1	$8.02\times 10^{-5}$	−1	$6.33\times 10^{-5}$	1	$7.5\times 10^{-5}$	1	$2.93\times 10^{-5}$	1	$5.62\times 10^{-5}$	1	0.03125	1	$2.93\times 10^{-5}$	1
D21	$1.71\times 10^{-5}$	1	$3.68\times 10^{-5}$	1	0.5	0	$2.86\times 10^{-5}$	−1	$8.14\times 10^{-5}$	1	$1.71\times 10^{-5}$	−1	$2.21\times 10^{-5}$	1	$2.21\times 10^{-5}$	1	$1.71\times 10^{-5}$	−1
D22	$6.32\times 10^{-5}$	1	$6.6\times 10^{-5}$	1	$7.67\times 10^{-5}$	1	0.000111	1	$8.46\times 10^{-5}$	1	$7.6\times 10^{-5}$	1	0.000136	1	$2.93\times 10^{-5}$	1	0.000219	1
D23	$8.72\times 10^{-5}$	1	$8.66\times 10^{-5}$	1	$8.62\times 10^{-5}$	1	$8.57\times 10^{-5}$	1	$8.77\times 10^{-5}$	1	$8.63\times 10^{-5}$	1	$8.79\times 10^{-5}$	1	$8.68\times 10^{-5}$	1	$8.45\times 10^{-5}$	1
D24	0.000167	1	0.000383	−1	0.000106	−1	0.000488	1	0.000182	1	0.000488	1	0.831055	0	$9.89\times 10^{-5}$	−1	0.000102	−1
D25	0.000172	−1	$8.26\times 10^{-5}$	−1	$7.87\times 10^{-5}$	−1	$8.29\times 10^{-5}$	−1	0.000149	−1	$8.26\times 10^{-5}$	−1	$8.2\times 10^{-5}$	−1	$8.13\times 10^{-5}$	−1	0.000173	−1
D26	$5.06\times 10^{-5}$	1	$4.15\times 10^{-5}$	1	$6.71\times 10^{-5}$	1	$3.56\times 10^{-5}$	1	$8.01\times 10^{-5}$	1	$3.56\times 10^{-5}$	1	$2.97\times 10^{-5}$	1	$6.1\times 10^{-5}$	1	0.0625	0
Won		19		17		18		15		21		19		17		17		11
Loss		3		3		5		8		3		5		4		6		7
Equal		4		6		3		3		2		2		5		3		8

**Table 20:** Average fitness values obtained using the SVM classifier.

Dataset	BCNRBO	BAOA	jBASO	BFPA	BCCSA
D1	0.042087	0.052315	**0.04181**	0.043411	0.051764
D2	**0.026922**	0.029438	0.028046	0.027089	0.038462
D3	**0.003559**	0.008973	0.005374	0.006526	0.018968
D4	**0.103026**	0.132914	0.11047	**0.103026**	0.228997
D5	0.002714	0.00333	**0.000464**	0.004946	0.128446
D6	**0.002857**	0.002929	0.010143	0.002929	0.032714
D7	**0.133878**	0.142605	0.134532	0.137054	0.16867
D8	**0.069131**	0.087015	0.070879	0.071219	0.110232
D9	0.074957	0.059756	**0.041463**	0.096432	0.155856
D10	**0.056664**	0.080616	0.060383	0.064825	0.122443
D11	0.493199	0.493694	**0.481346**	0.494964	0.494899
D12	0.346029	0.353868	**0.260346**	0.36085	0.360927
D13	**0.003344**	0.003875	0.0061	0.004188	0.006156
D14	0.106556	0.119536	0.094234	0.123946	**0.075**
D15	**0.038416**	0.04572	0.039973	0.043477	0.059828
D16	**0.175507**	0.177771	0.175729	0.175674	0.235227
D17	0.011849	0.016792	**0.011032**	0.01563	0.033219
D18	**0.065109**	0.079051	0.071333	0.072218	0.105526
D19	**0.164026**	0.184914	0.167689	0.168646	0.220882
D20	0.08585	0.101137	**0.085557**	0.087112	0.122881
D21	**0.215809**	0.224787	0.216717	**0.215809**	0.223143
D22	**0.244016**	0.245772	0.247879	0.244066	0.264272
D23	**0.259574**	0.266644	0.262705	0.263189	0.269106
D24	0.002838	0.004855	**0.002706**	0.004735	0.016674
D25	0.242086	0.229497	**0.204717**	0.253808	0.253579
D26	**0.086398**	0.087398	0.088112	0.08769	0.11092

**Table 21:** Friedman test results for BCNRBO vs. other algorithms with SVM classifier.

Dataset	BCNRBO	BAOA	jBASO	BFPA	BCCSA
D1	1.725	4.65	**1.675**	2.65	4.3
D2	**1.875**	3.225	2.85	2.05	5
D3	**1.65**	3.3	2.125	3.1	4.825
D4	**2.175**	3.575	2.3	**2.175**	4.775
D5	2.175	2.875	**1**	4.025	4.925
D6	**2.4**	2.525	2.925	2.5	4.65
D7	**1.725**	3.375	2.3	2.75	4.85
D8	**1.65**	3.5	2.35	2.525	4.975
D9	2.85	2.075	**1.275**	3.9	4.9
D10	**1.95**	3.15	2.075	2.925	4.9
D11	2.05	2.95	**1**	4.6	4.4
D12	2.4	2.35	**1.25**	4.325	4.675
D13	**1.725**	2.9	2.125	3.475	4.775
D14	1.9	2.85	**1.4**	4.05	4.8
D15	**1.725**	3.425	1.925	3.225	4.7
D16	**2.3**	2.7	2.5	2.6	4.9
D17	1.925	3.35	**1.6**	3.275	4.85
D18	**1.7**	3.3	2.175	2.825	5
D19	**1.725**	3.55	2.3	2.475	4.95
D20	**1.975**	3.625	2.3	2.2	4.9
D21	**2.1**	4.3	2.35	**2.1**	4.15
D22	**2.15**	2.925	2.9	2.25	4.775
D23	**1**	4.1	2.35	2.8	4.75
D24	1.575	3.175	**1.5**	3.85	4.9
D25	2.8	2.1	**1.2**	4.65	4.25
D26	**1.575**	2.975	2.375	3.4	4.675
Summation	**50.8**	82.825	52.125	80.7	123.55
Average	**1.953846**	3.185577	2.004808	3.103846	4.751923

**Table 22:** Average number of selected features (SVM).

Dataset	BCNRBO	BAOA	jBASO	BFPA	BCCSA
D1	21	**18.5**	28.65	24.65	35.2
D2	5	**4.7**	5.05	5.15	5.45
D3	**8.05**	11.15	9.55	16.95	14.85
D4	6	4.9	6.05	6	**4.65**
D5	15.2	18.65	**2.6**	27.7	26.3
D6	**2**	2.05	2.15	2.05	3.1
D7	**5.4**	5.65	6.25	6.2	6.25
D8	**13.85**	14.55	22.2	20.95	16.55
D9	22.35	23.85	**15.1**	35.3	29.65
D10	**6.75**	6.85	7.3	9.15	8.4
D11	22.9	27.85	**2.55**	40.55	39.9
D12	104.9	105.65	**29.95**	137.95	140.35
D13	**5.35**	6.2	5.8	6.7	9.85
D14	**2.6**	3.75	2.7	6.25	8.4
D15	8.8	11	**6.9**	16.1	13.9
D16	**3**	3.1	3.2	3.15	4.45
D17	7.95	10.95	**5.5**	15.35	15.55
D18	4.8	5.05	**4.55**	5.7	6.1
D19	**9.5**	10.25	13.45	13.5	11.3
D20	6.9	**6.05**	8.7	7.45	7.05
D21	7	**5.9**	6.95	7	7.95
D22	**5.05**	5.1	5.5	5.1	5.25
D23	**8.45**	10.2	11.2	12.2	18.4
D24	9.65	14.2	**9.2**	16.1	17.5
D25	308.2	298.25	**135.5**	351.55	334.3
D26	**3**	4.2	4.05	4.55	6.25

## Data Availability

All datasets used in this study are publicly available and were obtained from well-established repositories, including the UCI Machine Learning Repository, the KEEL Dataset Repository, Kaggle, and the Arizona State University (ASU) Feature Selection Repository. The datasets employed in this work include: Chess, Wisconsin, Breast, Olive, Lung Cancer, Diabetes (two variants), Heart, Ionosphere, Sonar, Lymphography, Hillvalley, LSVT, Zoo, Hepatitis, Diagnostic Breast Cancer, Coimbra, BreastEW, HeartEW, SPECT, Cleveland, ILPD, Parkinsons, Dermatology, PD Speech, and Heart Failure Clinical Records. For transparency and reproducibility, detailed information for each dataset—including the number of samples, number of features, original source repository, official URL, and available version details—has been provided in the public Zenodo repository associated with this work (https://doi.org/10.5281/zenodo.18602047). Readers are directed to the Zenodo archive for consolidated access and documentation of all datasets used in the experimental evaluation.

## Abbreviations

The following abbreviations are used in this manuscript:

ABC	Artificial Bee Colony
ACO	Ant Colony Optimization
BA	Bat Algorithm
BAOA	Binary Arithmetic Optimization Algorithm
BBA	Binary Bat Algorithm
BBO	Biogeography-Based Optimization
BCCSA	Binary Chaotic Crow Search Algorithm
BCNRBO	Binary Chaos-Enhanced Newton–Raphson-Based Optimizer
BDA	Binary Dwarf Mongoose
BDF	Binary Dragonfly Algorithm
BFPA	Binary Flower Pollination Algorithm
BPSO	Binary Particle Swarm Optimization
DT	Decision Tree Classifier
FP	Flower Pollination Algorithm
FS	Feature Selection
FSAs	Feature Selection Algorithms
GA	Genetic Algorithm
GOA	Grasshopper Optimization Algorithm
HSA	Harmony Search Algorithm
jBASO	Binary Atom Search Optimization
KNN	K-Nearest Neighbor Classifier
LSTM	Long Short-Term Memory
NB	Naive Bayes Classifier
NRBO	Newton–Raphson-Based Optimizer
PSO	Particle Swarm Optimization
SVM	Support Vector Machine
WOA	Whale Optimization Algorithm

## Appendix A. Supplementary Results

**Table A1:** Mathematical definitions and properties of chaotic maps.

Map No.	Map Name	Math Formula	Range
Map1	Chebyshev	$w_{i+1}=\cos\left(i\cos^{-1}\left(w_{i}\right)\right)$	(−1,1)
Map2	Circle	$w_{i+1}=\left(w_{i}+b-\left(\frac{a}{2\pi }\right)\sin\left(2\pi w_{k}\right),1\right),\;a=0.5,\;b=0.2$	(0,1)
Map3	Gauss/Mouse	$w_{i+1}=\left\{\begin{array}{cc} 1 & w_{i}=0\\ 1\;\mathrm{mod}\;(w_{i},1) & \mathrm{otherwise} \end{array}\right.$	(0,1)
Map4	Iterative	$w_{i+1}=\sin\left(a\pi w_{i}\right),a=0.7$	(−1,1)
Map5	Logistic	$w_{i+1}=aw_{i}\left(1-w_{i}\right),a=4$	(0,1)
Map6	Piecewise	$w_{i+1}=\left\{\begin{array}{cc} \frac{w_{i}}{p} & 0\leq w_{i}<p\\ \frac{w_{i}-p}{0.5-p} & p\leq w_{i}<0.5\\ \frac{1-p-w_{i}}{0.5-p} & 0.5\leq w_{i}<1-p\\ \frac{1-w_{i}}{p} & 1-p\leq w_{i}<1 \end{array}\right.$	(0,1)
Map7	Sine	$w_{i+1}=\frac{a}{4}\sin\left(\pi w_{i}\right),a=4$	(0,1)
Map8	Singer	$w_{i+1}=\mu \left(7.86w_{i}-23.31w_{i}^{2}+28.75w_{i}^{3}-13.302875w_{i}^{4}\right),\mu =1.07$	(0,1)
Map9	Sinusoidal	$w_{i+1}=aw_{i}^{2}\sin\left(\pi w_{i}\right),a=2.3$	(0,1)
Map10	Tent	$w_{i+1}=\left\{\begin{array}{cc} \frac{w_{i}}{0.7} & w_{i}<0.7\\ \frac{10}{3}\left(1-w_{i}\right) & w_{i}>0.7 \end{array}\right.$	(0,1)

**Table A2:** Worst obtained fitness values using KNN classifier.

Dataset	BCNRBO	BAOA	jBASO	BFPA	BBA	BCCSA	BDE	BABC	BPSO	BDA
D1	0.050078	0.076682	0.065728	0.068858	0.050078	0.064163	0.062598	0.051643	0.050078	**0.048513**
D2	0.014388	0.035971	0.021583	**0.007194**	0.021583	0.021583	0.035971	**0.007194**	**0.007194**	0.014388
D3	0.053097	0.035398	0.061947	0.061947	0.070796	0.053097	0.061947	0.044248	0.044248	**0.017699**
D4	0.087719	0.096491	0.087719	0.12281	0.096491	**0.070175**	0.10526	0.087719	0.087719	**0.070175**
D5	**0**	**0**	0.33333	0.16667	**0**	**0**	**0**	0.16667	**0**	**0**
D6	**0**	0.035714	0.035714	0.035714	0.035714	**0**	**0**	**0**	**0**	0.071429
D7	0.15517	**0.068966**	0.15517	0.086207	0.12069	0.22414	**0.068966**	0.17241	0.15517	0.13793
D8	0.085714	**0.042857**	0.11429	0.11429	0.14286	0.17143	0.085714	0.1	0.071429	0.057143
D9	**0.02439**	0.097561	0.14634	0.12195	0.04878	0.17073	0.19512	0.12195	0.04878	0.073171
D10	0.13793	0.13793	0.13793	0.17241	0.13793	0.2069	0.068966	0.10345	**0.034483**	0.13793
D11	**0.33884**	0.39669	0.43802	0.3719	0.42975	0.46281	0.39669	0.36364	0.40496	0.44628
D12	0.32	0.28	0.28	0.32	0.44	0.32	0.48	**0.2**	0.48	0.24
D13	0.05	0.05	0.05	0.05	**0**	0.05	**0**	**0**	**0**	0.1
D14	0.25	**0.125**	**0.125**	0.1875	0.1875	**0.125**	**0.125**	0.25	0.1875	**0.125**
D15	0.026549	0.053097	0.088496	0.044248	0.053097	0.088496	0.053097	**0.017699**	0.053097	0.061947
D16	0.086957	0.17391	0.26087	0.13043	**0**	0.17391	0.086957	0.086957	0.17391	0.13043
D17	**0.026549**	0.053097	0.044248	0.044248	0.035398	0.053097	0.035398	0.053097	0.035398	0.035398
D18	0.11111	0.12963	**0.074074**	0.12963	0.2037	0.33333	0.12963	0.12963	0.12963	0.14815
D19	0.22642	0.18868	0.22642	0.24528	0.22642	0.32075	0.22642	0.18868	0.22642	**0.15094**
D20	**0.10169**	0.15254	0.20339	0.16949	0.15254	0.25424	0.13559	**0.10169**	0.18644	0.11864
D21	0.22222	0.23529	**0.20915**	0.23529	0.24837	0.26144	0.22222	0.24183	**0.20915**	**0.20915**
D22	0.23276	0.22414	0.27586	0.26724	0.25	0.26724	0.26724	**0.21552**	0.25862	0.24138
D23	**0.005957**	0.026383	0.014468	0.025532	0.022128	0.02383	0.019574	0.020426	0.014468	0.019574
D24	**0**	**0**	**0**	**0**	**0**	0.041096	**0**	**0**	**0**	**0**
D25	0.22517	**0.21854**	0.25166	0.25166	0.2649	0.23179	0.25166	0.25166	0.23841	0.23179
D26	**0.11864**	0.13559	0.15254	0.18644	0.20339	0.15254	0.15254	0.13559	**0.11864**	0.13559

**Table A3:** Standard deviation of fitness values obtained using KNN classifier.

Dataset	BCNRBO	BAOA	jBASO	BFPA	BBA	BCCSA	BDE	BABC	BPSO	BDA
D1	0.006985	0.007478	0.008375	0.005409	0.007698	**0.00105**	0.005767	0.003255	0.003605	0.005619
D2	0.001609	**0**	0.003383	**0**	0.002953	0.003672	**0**	**0**	**0**	0.002953
D3	0.008614	0.001979	0.007651	0.003932	0.003958	0.005294	0.008322	0.002724	0.004331	**0**
D4	**0**	0.001962	0.010052	$7.12\times 10^{-17}$	0.003214	0.0027	$4.27\times 10^{-17}$	**0**	**0**	**0**
D5	**0**	**0**	0.097857	0.051299	**0**	**0**	**0**	$5.7\times 10^{-17}$	**0**	**0**
D6	**0**	**0**	**0**	**0**	**0**	**0**	**0**	**0**	**0**	**0**
D7	0.006316	0.008106	0.012999	0.00766	** $7.12\times 10^{-17}$ **	0.020534	0.0088	0.005307	0.008666	0.0088
D8	0.015001	0.00864	0.015701	0.006991	0.020454	0.010364	**0.006717**	0.011794	0.008794	0.010467
D9	0.012259	0.021344	0.020936	0.017472	0.01276	0.019823	0.021085	0.021085	**0.010836**	0.018175
D10	0.014151	0.016874	0.023995	0.017601	0.011188	0.035555	0.016212	**0**	**0**	0.01804
D11	0.008893	0.009843	0.018693	**0.00592**	0.014439	0.014003	0.007988	0.007806	0.009843	0.014314
D12	0.014654	0.02393	0.045837	0.018806	0.1126	0.016416	0.035303	** $8.54\times 10^{-17}$ **	0.026833	0.014654
D13	**0**	0.025521	**0**	0.018317	**0**	0.025521	**0**	**0**	**0**	0.01539
D14	0.019237	**0**	**0**	0.031414	0.057711	0.025649	**0**	0.036696	**0**	**0**
D15	**0**	0.003932	0.018931	**0**	0.016562	0.012671	0.005196	**0**	0.003932	0.008359
D16	$4.27\times 10^{-17}$	0.022304	0.027768	$5.7\times 10^{-17}$	**0**	0.051396	$4.27\times 10^{-17}$	$4.27\times 10^{-17}$	$5.7\times 10^{-17}$	$5.7\times 10^{-17}$
D17	**0**	0.005196	0.012992	0.002724	0.001979	0.004161	0.003932	0.001979	**0**	**0**
D18	0.009308	0.014839	** $2.85\times 10^{-17}$ **	0.011827	0.032019	0.045944	$5.7\times 10^{-17}$	0.014218	0.0076	0.017283
D19	0.017206	0.021117	0.023129	0.019622	0.025681	0.031154	0.017821	0.01608	**0.011288**	0.018237
D20	**0.00379**	0.016126	0.014653	0.005217	0.020502	0.027219	0.015035	0.010288	0.008294	0.018628
D21	$5.7\times 10^{-17}$	**0**	0.005807	0.001462	0.011473	0.019258	$5.7\times 10^{-17}$	**0**	$5.7\times 10^{-17}$	$5.7\times 10^{-17}$
D22	** $2.85\times 10^{-17}$ **	0.001928	0.010046	0.00383	0.017013	0.005867	0.009475	0.001928	$1.14\times 10^{-16}$	$1.42\times 10^{-16}$
D23	**0**	0.001598	**0**	0.000312	0.004778	0.001802	0.000478	**0**	**0**	**0**
D24	**0**	**0**	**0**	**0**	**0**	0.011675	**0**	**0**	**0**	**0**
D25	$1.42\times 10^{-16}$	0.014509	0.021322	**0**	0.027775	0.003114	0.005382	0.002426	0.015683	0.027756
D26	$7.12\times 10^{-17}$	$5.7\times 10^{-17}$	0.005217	**0**	$5.7\times 10^{-17}$	0.021227	0.007969	$5.7\times 10^{-17}$	$7.12\times 10^{-17}$	$5.7\times 10^{-17}$

**Table A4:** Average number of selected features using KNN classifier.

Dataset	BCNRBO	BAOA	jBASO	BFPA	BBA	BCCSA	BDE	BABC	BPSO	BDA
D1	18.35	**16.55**	18.2	23.45	19.8	35.35	22.05	23.7	20.45	21.95
D2	4.95	5.9	5.85	6.6	6	5.4	6.2	5.45	**3.65**	5.05
D3	**6.3**	12.9	10.8	18.9	14.1	20.9	16.45	17.9	13	13
D4	**4.05**	5.6	5.4	5.65	4.75	7.55	6	5.6	5.75	6
D5	**8.35**	27.65	20.8	34.8	26.85	27.85	35	28.4	27.25	27.95
D6	**2**	2.75	5.3	3.35	2.75	4.85	3.95	2.45	3	3.35
D7	**4.9**	5.05	5.7	6.2	6.45	5.8	6.45	6.25	5.45	5.05
D8	9.5	13.85	**4.35**	20.6	13.05	14.8	19.65	15.8	13.7	13.2
D9	23.2	24.75	**12.95**	37	25.65	29.35	37.45	34.75	29.55	27.6
D10	**5**	8.15	6.55	12.35	8.8	9.5	10.3	11.9	9.8	9.25
D11	43.2	38.75	**16.9**	60.6	47.3	49.65	63.35	64.6	46.3	45.25
D12	116.2	141.1	**59.75**	197.25	144.6	164.6	198.25	189.25	151.05	132.65
D13	**4.75**	7.95	9.4	10.15	8.45	9.95	10.35	9.45	9.05	10.1
D14	**4.2**	9.25	8.5	9.75	7.9	8.35	11.15	9.5	9.6	10.15
D15	**5.85**	13.15	8.85	18.85	14.05	16.85	17.6	17.5	13.2	13.65
D16	4	5.65	**3.55**	4.6	5.1	4.05	5.5	7.55	5.25	5.6
D17	**3.95**	13.9	13.55	20.5	14.25	20.85	18.5	15.55	15.15	15.25
D18	5.45	4.8	7.95	7.1	5.8	5.45	7.75	7.25	5	**4.7**
D19	9.5	**6.5**	12.05	13.75	9.1	10.6	12.2	12.3	11.55	10.65
D20	**3.4**	6.6	4.45	7.5	6.3	6.1	6.45	6.25	4.85	5.45
D21	4	4.9	4.4	3.05	5.35	4.75	5.1	**2.55**	5.15	5.45
D22	3	4.2	**2.7**	4.4	5.55	5.55	3.1	4.05	5.35	3.95
D23	**3.05**	6.3	8.8	11.15	6.5	8.95	10.2	9	5.75	6.85
D24	**11.5**	17.05	16.8	22.25	19.6	18.25	21.4	21.6	18.5	19.5
D25	302.15	310.1	**111.9**	491.35	374.55	372.45	482.1	398.55	379.7	368.35
D26	**2**	5.65	4.3	5.5	6.45	5.85	6.35	8	6	5.35

**Table A5:** Worst obtained fitness values using DT classifier.

Dataset	BCNRBO	BAOA	jBASO	BFPA	BBA	BCCSA	BDE	BABC	BPSO	BDA
D1	0.010955	0.050078	0.015649	0.010955	**0.007825**	0.017214	0.014085	0.00939	0.017214	0.018779
D2	**0.007194**	0.014388	0.043165	0.028777	0.05036	0.043165	0.035971	0.014388	0.035971	0.014388
D3	**0.00885**	0.061947	0.026549	0.026549	0.017699	0.044248	0.035398	0.017699	0.044248	0.044248
D4	0.078947	0.078947	0.087719	0.04386	0.087719	0.078947	0.061404	**0.026316**	0.070175	0.070175
D5	0.333333	0.166667	0.166667	0.166667	0.166667	0.166667	**0**	0.166667	**0**	0.166667
D6	**0**	0.107143	0.071429	0.035714	0.035714	0.035714	0.035714	0.035714	0.071429	0.035714
D7	0.12069	0.172414	**0.086207**	0.155172	0.137931	0.137931	0.12069	0.137931	0.137931	0.103448
D8	**0.028571**	0.057143	0.057143	0.042857	0.057143	0.071429	0.042857	**0.028571**	0.085714	**0.028571**
D9	**0.073171**	0.097561	0.146341	0.146341	0.097561	0.243902	0.170732	0.146341	0.097561	0.097561
D10	0.137931	0.137931	0.103448	0.068966	0.068966	0.206897	0.103448	**0.034483**	0.137931	0.137931
D11	0.322314	0.371901	0.355372	0.338843	**0.297521**	0.396694	0.347107	0.322314	0.355372	0.31405
D12	0.12	0.08	0.12	0.12	**0.04**	0.24	0.08	**0.04**	0.16	0.12
D13	**0**	**0**	0.05	0.05	0.05	0.05	0.05	**0**	**0**	0.05
D14	**0.0625**	0.1875	0.25	0.125	0.25	0.25	0.125	0.125	0.1875	0.125
D15	**0.017699**	0.053097	0.035398	0.035398	**0.017699**	0.035398	0.035398	0.044248	0.035398	0.026549
D16	0.173913	0.086957	0.086957	0.086957	0.217391	0.347826	0.130435	0.217391	**0**	0.173913
D17	0.026549	0.026549	0.044248	0.026549	0.035398	0.070796	0.035398	0.017699	0.00885	**0**
D18	0.185185	0.166667	0.12963	0.185185	0.092593	0.185185	0.092593	0.148148	**0.055556**	0.092593
D19	0.245283	0.188679	0.226415	0.226415	0.188679	0.283019	0.188679	**0.169811**	0.226415	0.207547
D20	**0.118644**	0.135593	0.135593	0.135593	0.152542	0.186441	**0.118644**	0.135593	0.152542	**0.118644**
D21	0.281046	0.281046	0.235294	0.235294	0.261438	0.261438	0.24183	**0.215686**	0.248366	0.248366
D22	**0.232759**	0.241379	0.267241	0.25	0.301724	0.310345	**0.232759**	**0.232759**	0.25	0.25
D23	**0.035745**	0.084255	0.085106	0.058723	0.075745	0.098723	0.07234	0.065532	0.051064	0.051064
D24	**0**	0.013699	0.027397	0.027397	0.013699	0.041096	0.013699	0.013699	0.041096	0.013699
D25	**0.086093**	0.119205	0.125828	0.10596	**0.086093**	0.198675	0.13245	0.152318	**0.086093**	0.099338
D26	0.118644	0.152542	0.118644	0.118644	0.135593	0.186441	0.118644	**0.067797**	**0.067797**	0.118644

**Table A6:** Standard deviation fitness value Std (DT).

Dataset	BCNRBO	BAOA	jBASO	BFPA	BBA	BCCSA	BDE	BABC	BPSO	BDA
D1	0.002154	0.007421	0.001788	0.001984	0.0007	**0**	0.00194	0.001124	0.00232	0.001893
D2	**0**	0.001609	0.007041	**0**	0.005462	0.005006	0.001609	**0**	0.001609	**0**
D3	0.004331	0.006594	0.004331	0.006074	0.003632	0.006734	**0.001979**	0.003932	0.006718	0.006023
D4	**0**	0.005324	0.007129	**0**	0.001961	0.013311	**0**	**0**	0.003923	**0**
D5	0.068399	0.061058	0.061058	$5.7\times 10^{-17}$	0.051299	0.061058	**0**	0.061058	**0**	$5.7\times 10^{-17}$
D6	**0**	$4.27\times 10^{-17}$	0.017951	**0**	**0**	0.007986	**0**	**0**	**0**	**0**
D7	0.008437	0.007911	** $4.27\times 10^{-17}$ **	0.008437	0.014013	0.011734	0.003855	$5.7\times 10^{-17}$	$5.7\times 10^{-17}$	0.008106
D8	0.009583	0.010234	0.013027	0.00864	0.008161	0.010968	**0.003194**	0.003194	0.009385	0.004397
D9	0.014321	0.018175	0.029048	0.015014	0.018175	0.027298	**0.010908**	0.018726	0.024262	0.028832
D10	0.016212	0.015421	**0**	0.010614	0.021227	0.026237	**0**	**0**	0.025998	0.007711
D11	0.013272	0.013567	0.012655	0.009888	0.020578	**0.006823**	0.009988	0.011533	0.010548	0.01584
D12	0.025547	0.01777	0.02285	0.008944	**0**	0.030435	0.008944	**0**	0.036419	0.019574
D13	**0**	**0**	0.02052	0.01118	0.025131	0.022213	0.01539	**0**	**0**	**0**
D14	0.031901	0.022897	0.038474	0.030585	0.030585	0.047986	0.034382	**0**	0.019237	0.032062
D15	0.003242	0.005294	0.006074	0.006974	0.004331	0.009894	0.004161	**0.001979**	0.006594	0.004517
D16	$5.7\times 10^{-17}$	$4.27\times 10^{-17}$	$4.27\times 10^{-17}$	$4.27\times 10^{-17}$	0.019316	0.046518	$5.7\times 10^{-17}$	0.015928	**0**	$5.7\times 10^{-17}$
D17	0.005814	0.005294	0.006959	0.003632	0.008566	0.006718	0.004448	0.001979	0.001979	**0**
D18	0.013799	0.018904	0.008707	0.008282	**0**	0.013799	**0**	0.0095	**0**	0.009452
D19	**0.010778**	0.014838	0.018967	0.014225	0.013516	0.012841	0.011288	0.016196	0.017821	0.016051
D20	0.007777	0.014445	0.009525	0.012142	0.006209	0.017798	** $7.12\times 10^{-17}$ **	0.006209	0.006209	0.008867
D21	$5.7\times 10^{-17}$	0.004294	0.003336	**0**	0.005846	0.01112	**0**	$5.7\times 10^{-17}$	0.001461	0.001461
D22	0.002653	0.005917	0.008518	**0**	0.012151	0.018736	0.008609	0.004219	0.00383	0.002653
D23	0.004463	0.012468	0.023872	**0.004434**	0.023522	0.009328	0.005743	0.006664	0.006399	0.010421
D24	**0**	**0**	0.006441	0.005018	0.003063	0.008777	0.003063	**0**	0.004216	0.006086
D25	0.009433	0.007474	0.010802	0.008754	0.011625	0.01615	0.007126	**0.005028**	0.00603	0.010438
D26	$7.12\times 10^{-17}$	**0**	0.006209	0.01263	0.005217	0.022988	$7.12\times 10^{-17}$	0.00753	**0**	$7.12\times 10^{-17}$

**Table A7:** Average number of selected features (DT).

Dataset	BCNRBO	BAOA	jBASO	BFPA	BBA	BCCSA	BDE	BABC	BPSO	BDA
D1	22.2	**19.95**	28.65	28.3	27.55	36	27.9	28.8	25.25	26.1
D2	4	5.45	**3.35**	4.05	4.4	4.6	4.8	5.15	5.25	4.6
D3	**7.85**	11.6	10.4	16.75	14.75	14.6	18.6	17.1	13.7	11.4
D4	5	**4.9**	5.6	5.55	5	5.3	6	6.65	6	6.15
D5	**12.5**	27.7	26.1	37.45	28.7	27.9	34	26.75	27.15	26.45
D6	**2**	3.7	3.8	4	4.25	4.05	5	2.7	2.75	3.65
D7	6.1	5.8	6.15	8.4	**5.15**	5.45	7.7	9.7	5.45	7.15
D8	**11.25**	13.9	14.95	20.85	16.4	17.85	22.2	19.7	14.4	18
D9	**20**	27	23.8	39.05	28.85	30.05	39.6	36.65	30.3	26.45
D10	**6**	7.45	9.5	8.4	8.65	9.15	11.65	11.95	8.15	8.45
D11	45.9	40.7	**26.2**	62.65	45.75	74.85	64.25	62.2	48.55	50.85
D12	124.65	139.25	**105**	196.7	152.25	151.95	198.8	173.95	150.6	155.05
D13	**3.45**	7.5	8.5	9.5	8.05	7.95	9.85	10.35	8.2	8.45
D14	**4.15**	7.05	5.25	9.35	6.9	9.45	11.15	10.6	8.6	7.6
D15	**7.9**	11.6	12.25	17.1	14.5	15.15	17.7	16.9	12.55	13.45
D16	**3**	6.75	4.7	5.8	4.25	5.5	4.45	3.05	5.6	4.75
D17	8.15	12.9	**7.75**	18.05	12.4	14.8	18	16.4	15.65	14.6
D18	**5.35**	5.85	6.3	6.45	6.7	9.45	8.1	5.5	5.6	5.6
D19	**5.15**	10.4	12.25	10.4	10.5	11.7	14.2	11.25	7.85	10.05
D20	**5.4**	5.65	6.55	7.05	5.5	9	7.5	7.05	6.45	5.5
D21	**3**	3.45	5.45	4	6.7	6.35	6	5	3.15	5.05
D22	4.15	4.1	4.05	6.55	4.4	4.85	4.55	**3.7**	4.75	4.1
D23	4.5	7.5	**3.95**	10.7	8.9	11.3	6	4	6.8	4.4
D24	**9.25**	19.5	16.65	19.9	16.85	21.75	17	18	17.5	18.3
D25	353.5	311.7	**278.05**	487	377.75	375.85	472	402	380.3	380.6
D26	4.15	5.8	5.05	5.8	4.85	6.2	5	**2**	6.85	7.1

**Table A8:** Worst obtained fitness values using NB classifier.

Dataset	BCNRBO	BAOA	jBASO	BFPA	BBA	BCCSA	BDE	BABC	BPSO	BDA
D1	0.172144	0.211268	0.175274	0.186228	0.165884	0.29108	0.179969	**0.158059**	0.175274	0.162754
D2	**0.007194**	0.014388	0.014388	0.021583	0.014388	0.043165	0.035971	0.021583	0.014388	0.05036
D3	0.017699	0.044248	0.017699	0.026549	0.035398	0.035398	0.044248	0.026549	**0.00885**	0.026549
D4	**0.035088**	**0.035088**	0.052632	0.070175	0.052632	0.070175	**0.035088**	0.061404	0.061404	0.04386
D5	0.166667	**0**	0.166667	**0**	**0**	0.166667	**0**	0.166667	**0**	0.166667
D6	**0**	**0**	0.035714	**0**	**0**	0.035714	0.035714	**0**	**0**	**0**
D7	**0.086207**	0.155172	0.189655	0.155172	0.103448	0.12069	0.172414	0.137931	0.12069	0.155172
D8	0.057143	0.057143	0.042857	0.057143	0.028571	**0.014286**	**0.014286**	0.042857	0.028571	0.028571
D9	0.170732	0.195122	0.121951	0.121951	**0.04878**	0.170732	0.195122	0.195122	**0.04878**	0.219512
D10	0.103448	0.137931	0.137931	0.068966	0.103448	0.241379	**0**	0.103448	0.137931	0.172414
D11	**0.396694**	0.504132	0.487603	0.413223	0.446281	0.421488	0.446281	0.495868	0.454545	0.471074
D12	0.16	0.16	0.2	0.24	0.2	0.28	**0.12**	0.2	0.16	**0.12**
D13	0.1	0.15	0.15	**0.05**	0.1	0.15	**0.05**	**0.05**	**0.05**	0.1
D14	0.0625	0.1875	0.1875	0.125	**0**	0.3125	0.125	0.0625	0.1875	0.1875
D15	**0**	0.044248	0.035398	0.044248	0.026549	0.017699	0.00885	0.026549	0.044248	0.026549
D16	0.173913	0.217391	0.217391	**0.086957**	0.173913	0.130435	0.217391	**0.086957**	**0.086957**	0.217391
D17	0.035398	0.017699	0.035398	0.044248	0.026549	0.061947	**0.00885**	0.017699	**0.00885**	**0.00885**
D18	**0.074074**	0.111111	0.092593	0.12963	0.148148	0.185185	0.12963	0.12963	0.111111	0.092593
D19	**0.113208**	0.226415	0.132075	0.188679	0.301887	0.301887	0.264151	0.188679	0.169811	0.245283
D20	0.084746	0.186441	0.135593	**0.067797**	0.186441	0.169492	0.101695	0.169492	0.118644	0.135593
D21	0.202614	0.24183	0.235294	0.196078	0.196078	0.261438	**0.156863**	0.202614	0.202614	0.176471
D22	**0.215517**	0.25	0.258621	0.284483	0.25	0.293103	0.25	0.224138	0.25	**0.215517**
D23	**0.224681**	0.251915	0.250213	0.261277	0.248511	0.258723	0.250213	0.26383	0.257872	0.241702
D24	0.054795	0.068493	**0.013699**	**0.013699**	**0.013699**	0.123288	**0.013699**	0.027397	**0.013699**	**0.013699**
D25	0.258278	0.225166	0.15894	0.211921	**0.145695**	0.231788	0.205298	0.205298	0.192053	0.231788
D26	0.101695	0.186441	0.186441	0.152542	0.152542	0.186441	0.135593	0.135593	**0.084746**	0.101695

**Table A9:** Standard deviation of fitness values obtained using NB classifier.

Dataset	BCNRBO	BAOA	jBASO	BFPA	BBA	BCCSA	BDE	BABC	BPSO	BDA
D1	**0.004694**	0.012759	0.005573	0.004725	0.007239	0.025584	0.005869	0.005105	0.00566	0.00771
D2	**0**	0.003672	0.003672	**0**	**0**	0.004429	**0**	**0**	**0**	0.001609
D3	0.004161	0.009523	0.006718	0.003932	0.004868	0.004517	0.003632	0.005196	**0**	0.00567
D4	**0**	0.0036	0.008478	**0**	**0**	0.008285	**0**	**0**	0.001961	0.001961
D5	0.08507	**0**	0.068399	**0**	**0**	0.037268	**0**	$5.7\times 10^{-17}$	**0**	$5.7\times 10^{-17}$
D6	**0**	**0**	0.013084	**0**	**0**	0.007986	**0**	**0**	**0**	**0**
D7	0.003855	$8.54\times 10^{-17}$	0.015294	0.015698	0.003855	0.008666	0.00766	0.012999	** $7.12\times 10^{-17}$ **	0.015294
D8	0.011234	0.006389	0.008546	0.004397	0.007859	0.006347	**0**	0.006389	0.00718	0.007292
D9	0.025611	0.016972	0.023206	0.015577	0.017472	0.020019	0.015577	0.016361	**0.013418**	0.033456
D10	0.017689	0.017332	0.020246	0.015319	0.023132	0.039554	**0**	0.007711	0.014151	0.020246
D11	0.006231	0.005672	0.009327	0.004218	0.005295	0.006823	** $5.7\times 10^{-17}$ **	0.001848	0.003672	0.004998
D12	0.020417	0.024192	0.042252	0.016416	0.029019	0.024192	0.008944	**0.008944**	0.018353	0.020926
D13	0.01118	0.01539	0.036635	**0**	0.01118	0.01539	**0**	**0**	**0**	0.01118
D14	0.013975	0.031414	0.027766	**0**	**0**	0.038474	**0**	0.013975	**0**	0.044887
D15	**0**	0.004517	0.006356	0.005742	0.005936	0.006023	**0**	0.003242	0.00567	0.00454
D16	$5.7\times 10^{-17}$	0.017843	0.009722	0.009722	0.017843	0.019316	$5.7\times 10^{-17}$	** $4.27\times 10^{-17}$ **	** $4.27\times 10^{-17}$ **	0.009722
D17	0.007192	0.001979	0.004448	0.008172	0.00454	0.008554	0.003632	**0**	0.001979	0.004517
D18	0.009062	0.012423	0.0076	** $5.7\times 10^{-17}$ **	0.009062	0.0152	0.0057	0.009062	0.004141	0.012166
D19	**0**	0.009483	0.006912	0.011411	0.01295	0.020555	0.008871	0.007743	0.014225	0.01295
D20	0.006956	0.008651	0.01263	0.005217	0.012867	0.013329	**0**	0.007969	0.008294	$5.7\times 10^{-17}$
D21	0.002012	**0**	0.005237	$8.54\times 10^{-17}$	0.004384	0.011276	$5.7\times 10^{-17}$	$8.54\times 10^{-17}$	$8.54\times 10^{-17}$	$8.54\times 10^{-17}$
D22	0.014151	0.004763	0.005062	0.008518	0.013089	0.014061	0.007861	0.004333	**0**	0.00383
D23	**0.002864**	0.004181	0.005335	0.00455	0.00447	0.007509	0.004923	0.00507	0.003888	0.004183
D24	0.012472	0.011988	0.006086	0.005018	0.006704	0.027924	0.004216	0.004216	0.004216	**0.003063**
D25	0.010186	0.006039	0.007717	0.006887	0.007865	0.008452	0.005644	**0.00423**	0.006115	0.00909
D26	0.00753	0.006209	0.00379	0.008294	**0**	0.018321	$5.7\times 10^{-17}$	0.00753	**0**	**0**

**Table A10:** Average number of selected features (NB).

Dataset	BCNRBO	BAOA	jBASO	BFPA	BBA	BCCSA	BDE	BABC	BPSO	BDA
D1	16.95	15.45	**15.3**	22.25	17.8	17.9	23.05	22	18.65	18.8
D2	**3**	5.8	4.65	8	5.8	4.9	5.3	4.2	5.85	4.35
D3	**6.65**	11.35	7.4	16.85	11.8	15.65	17.7	18	14	12.75
D4	5.05	6.2	5.55	6.25	5.8	**4.95**	6	5.35	5.85	6.05
D5	**11.05**	27.3	24.5	36.65	28.2	25.1	35.45	27.55	26.65	28.3
D6	**2**	4.45	3.35	3.55	3.95	3.5	5.3	2.7	2.5	3.9
D7	**3.2**	5.6	6.3	6.05	5.65	6.6	7.05	7.65	7.35	3.5
D8	**10.6**	12.8	13.45	20.95	13.1	24.1	20	18.05	15.1	16.35
D9	22.45	24	**11.9**	36.4	26.4	30.4	36.05	35.3	27.15	26.2
D10	**6.7**	8.25	9.6	11.5	8.4	8.05	11.95	10.9	8.2	8.7
D11	46.25	41.05	**8.7**	63.95	46.8	49.1	61.15	57.35	44.9	43.3
D12	122.25	130.05	**44.4**	191.8	148.8	152.9	196.75	196.15	154.65	139.95
D13	**8.3**	10	12	11.35	11.05	12.75	12.45	10.65	9.05	9.1
D14	**3.3**	6.35	9.4	9.9	11	9.35	10.2	10.75	9.05	6.45
D15	7.2	10.05	**6.1**	16.25	10.75	17.7	18.15	16.15	11.25	14.85
D16	**2**	3.05	4.15	4.05	4.9	4.5	6.05	6.1	5.45	3.85
D17	**8.35**	11.3	9.55	15.9	13.25	14.3	18.55	17.45	13.55	12.85
D18	6.9	6.6	6.65	9.25	8.95	7.6	7.8	6.5	**6.05**	6.85
D19	**3.5**	8.45	8.05	12.7	10.4	10.65	11.75	11.85	8.5	11.95
D20	**5.7**	7.45	8.15	8.6	6.5	8.45	7.75	7.9	6.65	6.7
D21	4.7	**2.5**	3.45	5.6	5	4.9	5	4.75	4	4.5
D22	3	3.6	4.75	**2.75**	5.2	4.6	3.4	3.15	3.35	5.7
D23	5.85	5.85	**4.15**	7.25	4.95	8.8	7.65	6.5	4.3	4.25
D24	**15.05**	17.5	18	22.95	18.8	18.6	23.85	22.7	19.05	21.7
D25	339.6	318.8	**114.3**	480.75	370.7	369.65	476.65	487	369.75	366.2
D26	4.85	6.3	**2.85**	4.85	4.75	4.65	3.8	4.55	3	6.6
